# Multivariant Transcriptome Analysis Identifies Modules and Hub Genes Associated with Poor Outcomes in Newly Diagnosed Multiple Myeloma Patients

**DOI:** 10.3390/cancers14092228

**Published:** 2022-04-29

**Authors:** Olayinka O. Adebayo, Eric B. Dammer, Courtney D. Dill, Adeyinka O. Adebayo, Saheed O. Oseni, Ti’ara L. Griffen, Adaugo Q. Ohandjo, Fengxia Yan, Sanjay Jain, Benjamin G. Barwick, Rajesh Singh, Lawrence H. Boise, James W. Lillard, Jr.

**Affiliations:** 1 Department of Microbiology, Biochemistry, and Immunology, Morehouse School of Medicine, Atlanta, GA 30310, USA; oadebayo@msm.edu (O.O.A.); cdill@msm.edu (C.D.D.); tgriffen@msm.edu (T.L.G.); sjain@msm.edu (S.J.); rsingh@msm.edu (R.S.); 2 Center for Neurodegenerative Disease, Emory University School of Medicine, Atlanta, GA 30322, USA; edammer@emory.edu; 3 Georgia Institute of Technology, Atlanta, GA 30332, USA; aadebayo51@gmail.com; 4 Department of Immunology, Moffitt Cancer Center, Tampa, FL 33612, USA; saheed.oseni@moffitt.org; 5 East-West Collaborative Research, Marietta, GA 30060, USA; adanmaqueen@gmail.com; 6 Department of Community Health and Preventive Medicine, Morehouse School of Medicine, Atlanta, GA 30310, USA; fyan@msm.edu; 7 Winship Cancer Institute, 1365 Clifton Road NE, Atlanta, GA 30322, USA; bbarwic@emory.edu (B.G.B.); lboise@emory.edu (L.H.B.)

**Keywords:** multiple myeloma, chemoresistance, ROC, log-rank, Kaplan–Meier, WGCNA

## Abstract

**Simple Summary:**

Multiple myeloma (MM) is a cancer of plasma cells with a five-year survival rate of 53%. MM is a heterogeneous disease with diverse clinical courses, consistent with the variable efficacy of therapeutic strategies and the development of chemoresistance. We used bioinformatic tools to better understand the molecular mechanisms that underlie failures in the standard treatment of MM with RVD (revlimid, velcade, and dexamethasone). Using an RNA-seq dataset from the MMRF CoMMpass study downloaded from the GDC portal, we identified modules positively correlated to MM vital status. Hub genes from these modules were further grouped based on their biological function and evaluated for association to patient survival.

**Abstract:**

The molecular mechanisms underlying chemoresistance in some newly diagnosed multiple myeloma (MM) patients receiving standard therapies (lenalidomide, bortezomib, and dexamethasone) are poorly understood. Identifying clinically relevant gene networks associated with death due to MM may uncover novel mechanisms, drug targets, and prognostic biomarkers to improve the treatment of the disease. This study used data from the MMRF CoMMpass RNA-seq dataset (N = 270) for weighted gene co-expression network analysis (WGCNA), which identified 21 modules of co-expressed genes. Genes differentially expressed in patients with poor outcomes were assessed using two independent sample *t*-tests (dead and alive MM patients). The clinical performance of biomarker candidates was evaluated using overall survival via a log-rank Kaplan–Meier and ROC test. Four distinct modules (M10, M13, M15, and M20) were significantly correlated with MM vital status and differentially expressed between the dead (poor outcomes) and the alive MM patients within two years. The biological functions of modules positively correlated with death (M10, M13, and M20) were G-protein coupled receptor protein, cell–cell adhesion, cell cycle regulation genes, and cellular membrane fusion genes. In contrast, a negatively correlated module to MM mortality (M15) was the regulation of B-cell activation and lymphocyte differentiation. MM biomarkers *CTAG2*, *MAGEA6*, *CCND2*, *NEK2*, and *E2F2* were co-expressed in positively correlated modules to MM vital status, which was associated with MM’s lower overall survival.

## 1. Introduction

Multiple myeloma (MM) is an incurable hematological malignancy with an unlimited proliferation of abnormal plasma cells in the bone marrow (BM) and high levels of monoclonal protein in the blood and urine [1]. MM slightly affects more males than females, with an average age of diagnosis of 65 years. African Americans have the highest incidence, while Native American/Pacific Islander and Asian persons have the lowest. The American Cancer Society estimates that 34,920 new cases of MM will be diagnosed in [2] 2021, with about 12,410 deaths. The advancement of novel chemotherapeutic agents and cell therapy has greatly improved for MM patients, and the five-year relative survival rate after MM diagnosis is 53% [1,2,3]. However, MM remains incurable mainly due to the development of resistance and disease relapse [4,5]. Therefore, continued exploration of novel prognostic biomarkers and therapeutic targets is crucial for MM patients.

Although the molecular mechanisms of MM pathogenesis are not well understood, many biomarkers for MM progression have been described, including *CTAG2*, *MAGEA6*, *CCND2*, *NEK2*, and *E2F2*, some of which could predict clinical prognosis and therapeutic effectiveness [6]. Stem cell transplant is the only known potential cure for MM in young patients with few comorbidities. Patients with MM diagnosis may be evaluated for either autologous stem cell transplantation [7,8,9].

In general, FDA-approved treatments for MM have increased in the last two decades. MM patients are now treated with triple-drug combinations to prevent the emergence of resistant clones. RVD (lenalidomide/revlimid, bortezomib/velcade, and dexamethasone) are now standard treatments for newly diagnosed MM patients who are either eligible for the autologous stem cell transplant or not [2,4]. These medications increase the survival rate of MM patients by targeting several mechanisms. For instance, dexamethasone induces apoptosis of Plasma Cell Myeloma (PCM) and reduces mitochondrial transmembrane potential. Lenalidomide has direct antiproliferative effects on plasma cells and activation of the immune cells within the bone marrow microenvironment. In addition, the binding of lenalidomide to cereblon (CRBN) triggers a change in CRBN targets initiating their therapeutic activity [10]. Bortezomib inhibits the action of the 26S proteasome leading to the inhibition of NF-KB activity and targeting protein homestasis, an important dependency in normal and malignant plasma cells, and downregulation of adhesion molecule expression on PCM cells [11]. Each class of agents has evolved as a second- or third-generation therapy with refined potency and safety compared to their predecessors [12], extending the median MM survival over 60 months from 24% to 54% [2].

Newer agents improve survival primarily by maintaining the stability of MM [13]. Immunotherapies include antibodies to CD38 and SLAMF7 and an antibody–drug conjugate that targets BCMA. In addition, the FDA approved chimeric antigen receptor (CAR) T cells for MM patients who have relapsed under conventional therapies. Nevertheless, the cure for MM remains elusive.

In this study, patient sample RNA-seq data provided by the Multiple Myeloma Research Foundation (MMRF) CoMMpass study (NCT01454297) was analyzed using WGCNA and other methods including gene set enrichment, Kaplan–Meier (KM) survival analysis, and ROC analysis to discover the relationships between clinical traits (vital status: “death” or “alive”) and transcript abundance, thereby identifying hub genes associated with early deaths due to MM (deaths within the first two years of diagnosis and treatment with RVD).This study intended to identify the gene expression patterns associated with poor survival. This study uses WGCNA to identify molecular signatures across transcriptomic networks of MM patients treated with RVD, while simultaneously testing associations to MM clinical traits used during the diagnosis and prognosis of the disease. Ultimately, this study aims to shed more light on understanding the molecular underpinnings of MM.

## 2. Materials and Methods

### 2.1. Data Curation and Normalization

**Figure 1:**
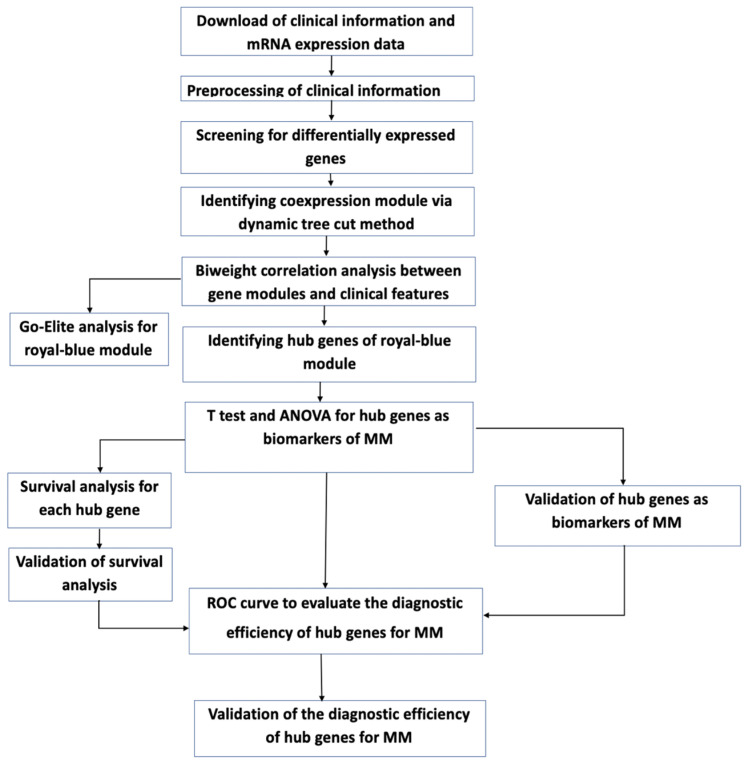
Workflow for the analysis of gene expression and clinical data.

Level 3 deidentified, RNA-seq fragments per kilobase per million mapped reads (FPKM) data were obtained from The Cancer Genome Atlas (TCGA) MMRF CoMMpass study, in which bone marrow samples were collected from newly diagnosed MM patients with informed consent and IRB approval [14]. An overview of the clinical trait details for the newly diagnosed MM patients, who were initially treated with RVD combination therapy (Table S1) and expression data availability (*n* = 270), is provided in (Table 1)**.** The RNA-seq and clinical data were downloaded on or before 27 September 2019, and analyzed using a bioinformatics pipeline (Figure 1).

### 2.2. Detecting Low Counts, Batch Effect Correction, and Removal of Outliers

The sequencing, alignment, transcript counting, and FPKM for each patient was performed using RNA-seq data as previously described [15,16]. MMRF CoMMpass MM RNA-seq FPKM data from multiple research centers (baseline case samples) with 60,478 gene-wise short read-based quantifications were curated to address potential technical or site-based variance. Batch effect control, normalization, and quality assessment tests were performed using a Tunable Approach for Median Polish of Ratio (TAMPOR): https://github.com/edammer/TAMPOR, accessed on 7 April 2022 [17,18]. All samples were processed together in the sample-gene transcript matrix to capture MM biological variance and preserve it through normalization. TAMPOR maintained the integrity of the data through robust batch effect correction by removing batch artifacts manifesting as batch-wise variance, genes with ≥50% missing or zero values. After this, samples with ≥50% zero values, technical replicates, and cluster outliers (26) were removed.

### 2.3. Coding Clinical Metadata for Biological Network Analysis

The clinical traits dataset contains phenotypes for each sample. The non-numeric variables were converted into numeric values. For instance, Gender (“1” = “Male”, “2” = “Female”, “N.A.” = “Unknown”), Race (“1” = “European American”, “2” = “African American”, “3 = “Others”), Ethnicity (“1” = “Hispanic or Latino”, “2” = “Not Hispanic or not Latino”, “3” = “Others”), and MM vital status (“0” = “Alive”, “1” = “Dead”).

### 2.4. Gene Clustering and Network Analysis

WGCNA is an R package used to identify gene co-expression networks in the MMRF CoMMpass study by robustly calculating the eigengene, bicor rho, and *p*-values for each module and then correlating the first principal component of each module (module eigengenes) with clinical traits or phenotypes of interest. The WGCNA package (WGCNA_1.70-3) was installed from the Comprehensive R Archive Network (CRAN), and all analyses were carried out in R version 3.6, with some system calls to Python v2.7. To reduce RNA-seq data dimensionality from thousands of genes (60,478 to 30,598 genes, *n* = 270 for RVD therapy-receiving patients) to a few modules, WGCNA was used to assess gene co-expression profiles across all MM samples.

A sample dissimilarity matrix (1-topology overlap) was constructed by WGCNA and genes that have similar expression patterns were grouped within the sample cohort. The network was constructed using the WGCNA blockwiseModules function [19], with parameters as follows: WGCNA dynamic tree-cutting algorithm, CutreeHybrid, power = 7, deepsplit = 2, minModuleSize = 180, mergeCutHeight = 0.15, TOMDenom = “mean”, corType = “bicor”, networkType = “signed”, pamStage = TRUE, pamRespectsDendro = TRUE, reassignThresh = 0.05, verbose = 3, saveTOMs = FALSE, maxBlockSize larger than the number of genes being clustered (30,598), and reassignThresh = 0.05. To limit the impact of high technical variation within RNA-seq data representing differences in transcript abundances across samples, biweight midcorrelation (bicor) was used instead of Pearson correlation to provide robust correlations with less weight given to outliers [19]. The WGCNA R-script and outputs for this study can be downloaded from: https://github.com/pog240/MMRF-WGCNA-ANALYSIS/ (accessed on 26 June 2021).

### 2.5. Gene Ontology (GO) Enrichment and Upstream Regulator Analysis

Gene Ontology Elite (GO Elite) (version 1.2.5) was used to perform gene set enrichment analysis on the biologically significant modules (M10, M13, M15, and M20) to identify overall module enrichment of biological functions, molecular processes, and cellular locations http://www.genmapp.org/go_elite/help_main.htm accessed on 7 April 2022. GO enrichment analysis used the Ensembl database (Version 62) of pre-defined gene lists organized by biological process, molecular function, and cellular component. Fisher’s exact test, adjusted for false discovery, was used to determine overrepresentation or significant overlap between WGCNA modules of interest members and pre-defined gene lists. The reference background list was the subset of 30,598 genes with symbols in the final cleaned-up abundance matrix (22,456 symbols). Additionally, gene set enrichment analysis (GSEA) was performed using the GSEA molecular signature C2 database (version 6.2) to identify associations between network modules and curated lists of genes related to various diseases, particularly cancer. The GSEA C3 database was also used to identify upstream regulators among the genes of interest in each module [20].

### 2.6. Differential Gene Expression

Differential expression via two independent sample *t*-tests, with equal variance, was conducted to identify gene candidates within significant modules (upregulated and downregulated modules) correlated with MM vital status (poor outcomes). An independent *t*-test was used to compare differential gene expression among patients who died (*n* = 58) versus those who survived within two years (*n* = 212) on RVD treatments. Data for bone marrow samples from patients who missed treatment or lacked vital status information were excluded from differential expression analysis. False discovery rate (FDR) adjustment was performed using the Benjamini–Hochberg method, with the threshold set at FDR <0.01 for the comparison. A total of 22,515 genes were visualized using the EnhancedVolcano R package and used for further analysis.

### 2.7. Protein-Protein Interaction within Modules of Interest

To determine known and predicted Protein–Protein Interaction (PPI) of significant DEGs in our modules of interest (M10, M13, M15, and M20), a Protein–Protein Interaction functional clustering enrichment analysis was performed using genes with a log fold change of 1.9 and above. This analysis was performed using the String database (version 11.0 https://string-db.org/ (accessed on 5 January 2022) Genes clustered based on their biological function (k-means) were further analyzed using Enrichr, a web tool, to visualize their collective functions. This PPI enhanced insight into the biological functions of genes in the modules of interest [21].

### 2.8. Geneset Enrichment Analysis

Enrichr, a comprehensive gene set enrichment server: https://maayanlab.cloud/Enrichr/ (accessed on 7 January 2022) was used to visualize clustergrams and to understand the overall biological knowledge for further biological discovery on the gene symbol list within modules of interest [22].

### 2.9. ROC Analyses

A Receiver Operator Curve (ROC) analysis was performed using the top over-expressed genes in modules positively correlated with MM vital status to determine if the differentially expressed genes could serve as predictive indicators of MM vital status (M10, M13, and M20). An EasyROC web tool on the default non-parametric test setting was used to perform ROC.

### 2.10. Survival Analyses

The prognostic value of MM patients treated with RVD for two years (*n* = 270) was evaluated using a KM plotter (www.kmplot.com (accessed on 10 January 2022)). MM expression data and their survival information for the samples were uploaded onto the KM plotter web-based tool. To analyze OS of MM patients, patient’s samples were split into two groups by median expression (high versus low expression) and assessed by a Kaplan–Meier survival plot, with the hazard ratio (HR) with 95% confidence intervals (CIs) and a log-rank (Mantel–Cox) test was used to determine *p*-values for both sets of KM analyses.

## 3. Results

### 3.1. Transcriptomic Analysis Defines a Network of MM Co-Expression Modules

**Figure 2:**
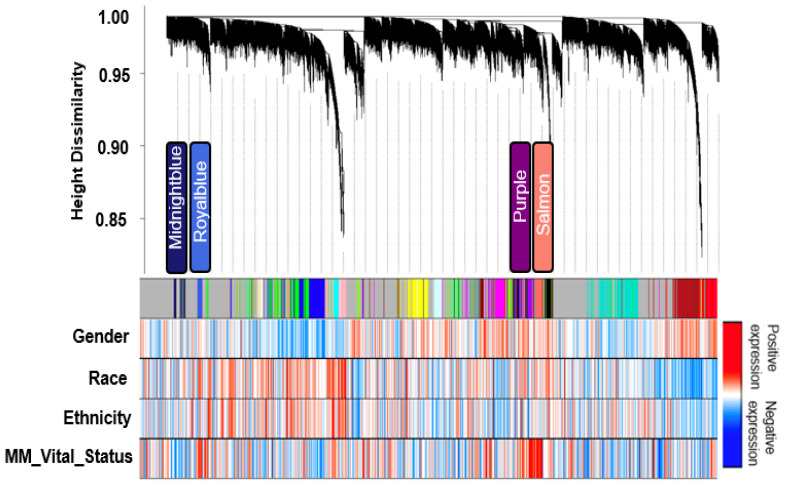
Clustering dendrogram of all expressed genes based on consensus topological overlap with the corresponding module colors and associated clinical traits. The top panel represents the gene dendrogram obtained by clustering the dissimilarity based on consensus topological overlap with the corresponding module colors indicated by the color row. Each colored row (module colors) represents a color-coded module containing a group of highly connected genes. Four biologically significant modules were identified from the 21 modules output from WGCNA. The relationship between each relevant clinical trait was assessed for each color-coded module. Bypassing the default Pearson’s correlation method in WGCNA, we applied biweight midcorrelation as a robust alternative implemented in its WGCNA function (*bicor*).

**Figure 3:**
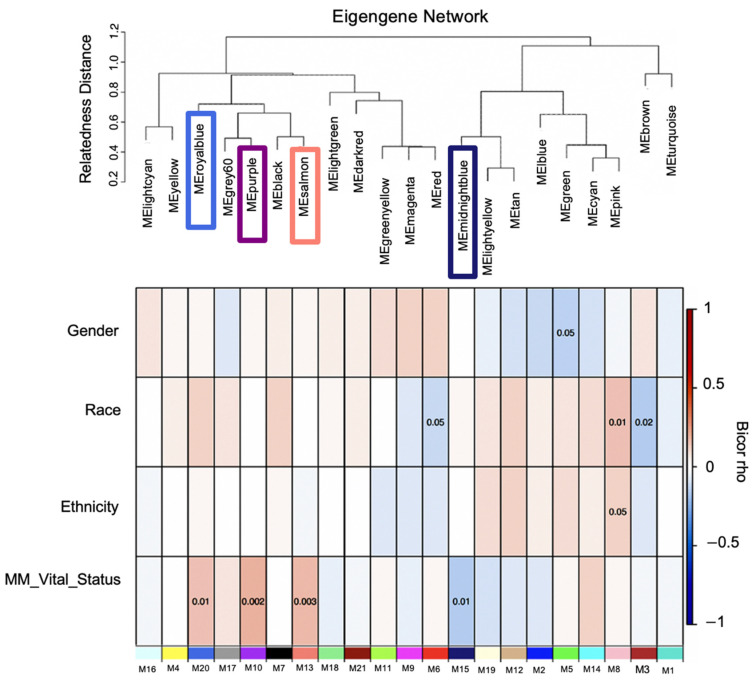
Module-relatedness clustering and module-trait correlations reveal modules associated with multiple myeloma clinical traits. The upper panel shows the dendrogram of consensus module eigengenes obtained by WGCNA on the consensus correlation. The lower panel shows the heatmap of module–trait relationships. Each row in the heatmap corresponds to a specific clinical trait and each column to a module. The module colors are shown at the bottom of each column. The boxes shaded orange are intended to highlight module–trait correlations with a significant *p*-value of < 0.05. Boxes shaded blue and orange denote negative and positive correlations to MM’s vital status, respectively. While the boxes are shaded, white depicts no correlation. Module–trait bicor color scale: blue = negative correlation white = no correlation and red = positive correlation indicates modules with a significant Student *t*-test *p*-value that cluster together.

The transcriptome comprising 30,598 genes across 270 MM case samples was examined for co-expression modules of gene transcripts and the network biology of MM poor outcomes was assessed. The distribution of reported clinical traits among MM patients and their therapy distribution is depicted in (Table S1), respectively. These clinical traits were obtained from the MMRF CoMMpass dataset. WGCNA identified twenty-one module eigengenes (MEs), numbered by their rank from the largest number of genes to the smallest, M1 to M21 (Figure 2; Table 2), expression correlation metric was determined by their relatedness and plotted as a dendrogram (Figure 3, upper panel). The relatedness dendrogram shows that M16 is closely related to M4. M20 is closely related to M17, M10, M7, and M13. M18 and M21 are separated but are closely related to M11, M9, and M6. Out of these modules, M10, M13 and M20 are positively associated with poor survival, while M15 is negatively associated with poor survival.

### 3.2. Identification of Transcript Significant Modules Associated with Mortality and Functional Annotation

**Figure 4:**
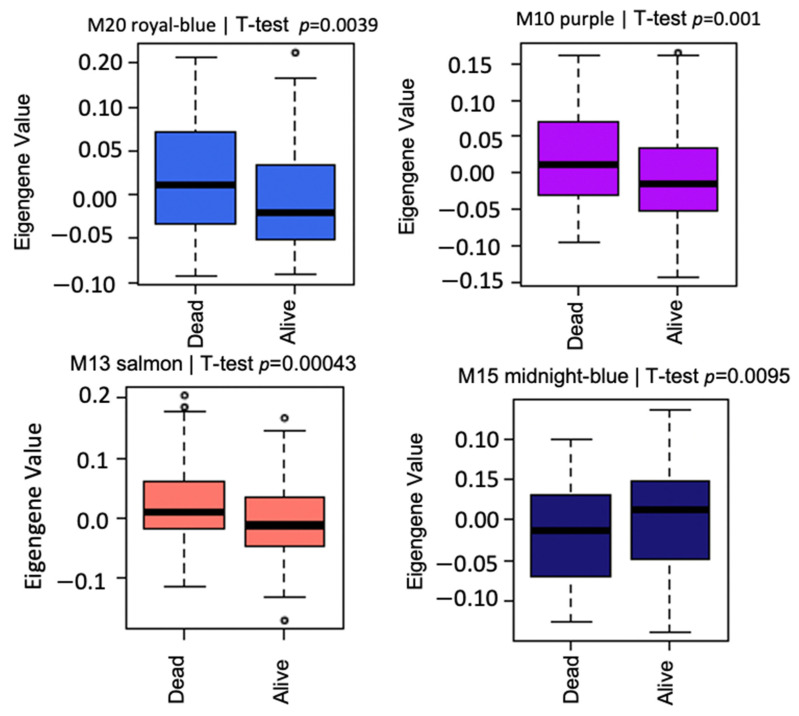
Box plots of differential expression patterns of module eigengenes across MM patient samples. Each color of the box represents the module and the associated *p*-values. Royalblue, salmon, and purple modules are positively correlated with vital status of MM patients on standard RVD therapy. However, the midnight blue module is negatively correlated with MM vital status. The small circles in the figure depict the outliers in the data.

The association of Module Eigengenes (MEs) to MM vital status was assessed by correlation. This helps determine which modules are candidates for molecular causality of the trait of interest in MM patients treated with RVD for two years (Table 1). A total of 21 modules and corresponding module eigengenes were constructed to determine the module networks of interest associated with vital status. The robust correlation of the 21 modules to vital status helped determine the transcriptome networks of interest. The red color represents positive gene expression, while the blue color represents negative gene expression (Figure 2 heatmap). These analyses identified positive correlations with vital status in the M20 (*r* = 0.150, *p* = 0.0039) with 307 genes, the M13 (*r* = 0.18, *p* = 0.00043) with 568 genes, and the M10 (*r* = 0.18, *p* = 0.001) with 690 genes, but a negative correlation with vital status for the M15 (r = −0.15, *p* = 0.0095) with 534 genes. Additionally, we performed a *t*-test to compare the expression of MM patients who died and those alive for two years on RVD treatments (Figure 4).

### 3.3. Modules Differentiating between Alive and Dead Patients Represent Known Biological Groundworks of MM

**Figure 5:**
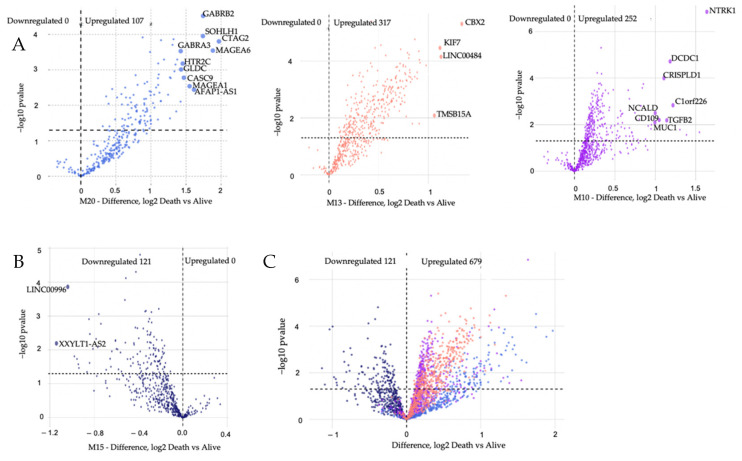
Volcano plots of differential gene expression identify gene transcripts that contain upregulated genes, panel (**A**), downregulated genes, panel (**B**), and both upregulated and downregulated genes, panel (**C**), based on MM vital status for biologically significant modules. The number of downregulated genestranscripts is denoted on the left and the number of upregulated gene transcripts is denoted on the right. The total number of upregulated genes is 676, while the total number of downregulated genes is 121. The log2 fold change is plotted on the *X*-axis, and the negative log10 *p*-value is plotted on the *y*-axis. Gene transcripts are colored by module membership.

We hypothesized that modules with positive clinical correlations would have high gene expression based on the vital status trait correlation. In contrast, the negatively correlated modules would have low expression in MM patients who died while on RVD treatment. *t*-test analysis was used to test this hypothesis. A Tukey post hoc test confirms elevated expression of modules (M10, M13, M20) and lower expression (M15), which were viewed via Volcano plots (Figure 5). A total of 121 genes were downregulated, and 676 genes were upregulated when we compared the dead and alive MM patients. DEGs from positively correlated significant modules with the highest log2 fold change values were selected as genes of interest, and these genes were in the royal blue module (Table S2). The biological functions associated with upregulated genes in the royal blue module are skeletal system morphogenesis [23], G protein-coupled receptor (GPCR) protein signaling pathways [24], multicellular organismal development, synaptic transmission [25], and cell–cell adhesion [26]. We observed that some of the top differentially expressed genes are known MM biomarkers: *CTAG2*, *MAGEA6*, *MAGEA1*, and *SSX1*, whereas some genes within the M20 module were differentially expressed, but not well known in the context of MM biology (*SOHLH1*, *GABRA3*, *GABRB2*, *HTR2C*, and *GLDC*). We also noted the top differentially expressed genes in each vital status-associated module based on (−log10 (*p*-value) = 2 and Diff log2 = 1) (Figure 5): *NTKR1*, *MUC1*, *C1orf226*, *DCDC1*, *TGFB2*, *CRISPLD1*, *CD109* and *NCALD* (M10). *CBX2*, *LINC00484*, *KIF7* and *TMSB158 (M13). CTAG2*, *MAGEA6*, *GABRB2*, *SOHLH1*, *AFAP1-AS1*, *MAGEA1*, *CASC9*, *HTR2C*, *GLDC* and *GABRA3* (M20).

**Figure 6:**
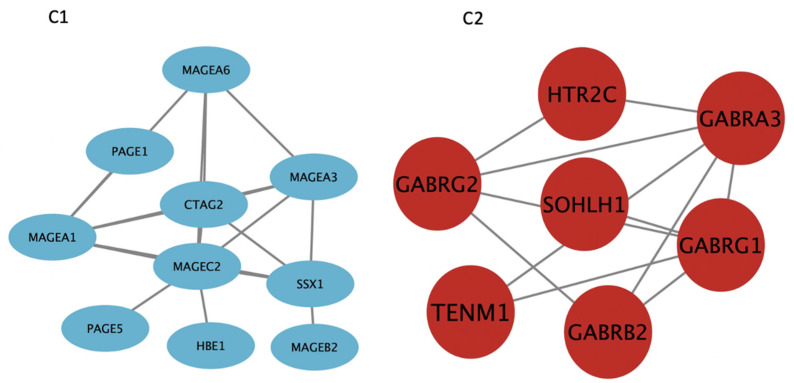
String was used to detect predictive interactions between the top fold change genes from the royal blue module. K-means clustering algorithm was used to cluster the genes based on the biological functions. Genes denoted with aqua blue (**C1**) nodes are mainly from the *MAGE* (Melanoma Antigen Gene) family of genes. Genes clustered with red nodes (**C2**) are mainly from the Gamma-aminobutyric acid receptor genes, which are major inhibitory neurotransmitters in the mammalian brain. To change the size and color of each node, we uploaded the clustered genes into Cytoscape.

To understand how the genes in M20 are biologically connected in terms of their function, we used the String database. Using the K-means clustering algorithm, differentiated genes in M20 were clustered into two groups (aqua and red nodes) (Figure 6). Genes denoted with aqua nodes mostly form the *MAGE* (*Melanoma Antigen Gene*) family of genes. On the other hand, the gene cluster denoted with red nodes is mostly Gamma-aminobutyric acid receptor genes, which are ligand-gated chloride channels activated by major inhibitory neurotransmitters in the mammalian brain. GABA genes interact with other genes in the M20, such as *TENM1*. *MAGE* also interacts with *HTR2C*, a G-protein coupled receptor and Serotonin receptor. Additionally, *MAGE* interacts with *SOHLH1*, a male and female germline differentiation transcription regulator. (Figure 6).

### 3.4. Gene Set Enrichment, ROC Curve, and Kaplan–Meier Analyses

To summarize our differentially expressed genes’ biological function and enable further downstream analysis between the blue and the red gene sets cluster from the string database, we used Enrichr. Enrichr can assign the genes their GO terms, which it retrieves from more than 192 gene set libraries (https://maayanlab.cloud/Enrichr/ (accessed on 15 January 2022)). The tool mapped most of our significant differentially expressed genes (*GABRB2*, *SOHLH1*, *MAGEA1*, *GABRA3*, *HTR2C*, *GABRG1*, and *GABRG2*) to mobilized *CD34* primary cells with a *p*-value of 0.02 and odds ratio of 3.5 (Table S3). *CD34* is expressed on hematopoietic stem cells and non-hematopoietic cells (mesenchymal stem cells, endothelial cells, etc.,) [27,28,29,30]. It has been shown previously that CD34 (+) cells frequently underwent cellular division and formed rapid colonies [31] Next, we hypothesized that genes associated with CD34 (hematopoietic stem cell) mobilization could be used to predict poor outcomes in MM (Figure S1).

To identify specific genes associated with poor outcomes in MM, we conducted Receiver Operating Characteristic (ROC) analysis. Among our gene list, *NTRK1* (0.71), *GABRB2* (0.67), *SOHLH1* (0.65), *GABRA3* (0.64), *DCDC1* (0.64), *MAGEA1* (0.63), and *HTR2C* (0.63) have the highest AUC scores of predicting poor outcomes in MM (Table 3). At the same time, *GABRG1* (0.57) and *GABRG2* (0.60) have the lowest AUC scores for predicting poor outcomes in MM. To improve the predictive ability of AUC, we combined the AUCs of the highest predictive scores, and there were no significant differences. The ROC result from the MMRF dataset shows that our genes of interest from the royalblue module are not promising MM biomarkers due to their low AUC values, but they have significant ***p***-values. To validate this result, we downloaded a microarray dataset of 602 individuals with MM disease from the Gene Expression Omnibus (GEO) database (GSE83503) [32]. The dataset was log2 transformed before the ROC analysis and processed similarly as the MMRF dataset. The AUC scores from GEO dataset aligned with that of the MMRF CoMMpass database (Table S6A,B).

**Figure 7:**
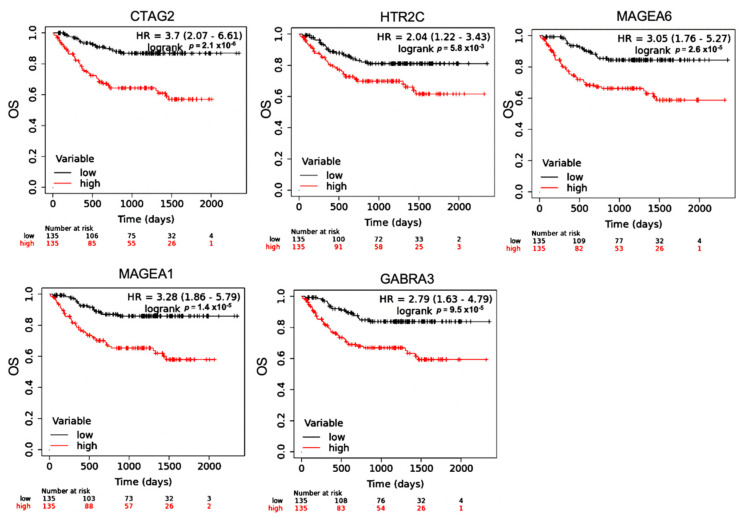
Overall survival of differentially expressed hub genes with *p*-values < 0.05, kME > 0.7 and Log Fold Change (LFC) ≥ 0.5 from the M20 module (*CTAG2*, *GABRA3*, *MAGEA1*, *MAGEA6*, and *HTR2C*) were analyzed using KMplotter. The patient samples were split based on the median expression. The red lines denote samples with high gene expression, and the black lines refers to samples with low expression. Hazard ratios (HR) are reported for low expression of the genes.

To determine the effect of differentially expressed hub genes on MM overall survival outcomes, we performed a log-rank Kaplan–Meier (KM) analysis using a KM plotter (Figure 7). Hub genes were selected for this analysis as they would make good therapeutic candidates due to their ability to regulate multiple gene expressions. Differentially expressed hub genes (*p*-value < 0.05, kME > 0.7, Log Fold Change (LFC) ≥ 0.5) from the modules positively correlated with MM vital status (M10, M13 and M20) were evaluated (Table S4, Figure S2). KM analysis showed significant difference in the OS of patients with low vs. high expression of our genes of interest. However, M10 did not satisfy the cutoff points. Additionally, a KM plotter was used to calculate the Pearson correlation between the genes in M20 and M13, respectively. *MAGEA6* and *GABRA3* in M20 have a positive linear correlation of 90%, while KIF14 and CENPF in M13 have a positive linear correlation of 94% (Table S5A,B).

## 4. Discussion

MM is a heterogeneous hematological malignancy, so it is crucial to explore the molecular mechanisms to better understand the disease. The complexity of molecular mechanisms of cancer can be deciphered using a network biology approach [33]. A network biology approach was applied in our study to identify gene transcript co-expression networks driven by MM gene expressions and correlate them to our clinical outcome of interest (vital status). The modules that are highly correlated with the clinical traits have both known and novel mechanisms of MM.

WGCNA, a novel transcriptomic analysis tool, was used to identify transcript expression signatures among MM patient samples derived from a RNAseq dataset. Co-expression networks are calculated without supervision and display clusters of genes controlled by the same transcription factors, have the same function, and therefore are co-regulated. Genes of the same signaling pathway can enrich module members [34]. WGCNA identified twenty-one modules, and their expression was correlated with our clinical trait of interest (MM vital status).

Out of the 21 modules, four modules were correlated with MM vital status. Three had positive correlations (M10, M13, and M20), and one demonstrated negative correlations (M15). We selected hub genes from the positive correlated modules based on a combined cutoff point (*p*-value < 0.05, kME ≥ 0.7 and LFC ≥ 0.5). Genes in M10 did not satisfy the specified criteria (*p*-value < 0.05, kME ≥ 0.7 and LFC ≥ 0.5) to be nominated as hub genes of interest. Therefore, genes from this module were not considered as part of the predictive analysis. The remaining two modules (M13 and M20) provide insights on the molecular signatures of MM vital status (mortality): cell proliferation, GCPR, and cell–cell adhesion. M13 contained genes involved with cell cycle regulation (proliferation genes), and M20 modules included genes involved in inhibitory synaptic transmission and *MAGE*. Previously, it has been shown that MM patients with high expression of cell cycle regulatory genes (proliferation genes) is associated with a central, independent prognostic factor) [35] and melanoma-associated antigen genes [36] have lower overall survival by mediating myeloma cell survival and drug resistance. In this study, we observed higher expression of cell cycle regulator genes (proliferation genes) (M13) in patients who experienced death, consistent with the poor clinical outcomes reported in the study mentioned above. Our study agrees with previous findings that detailed MM with higher expression of cell cycle regulation genes may be associated with poor outcomes [37]. The M13 and M20 modules contain both hub genes (kME ≥ 0.7) and differentially significant genes (*p* < 0.05 and fold change > 1.5) of MM (*NEK2*, *KIF14*, *CENPF*, *GABRA3*, *RRM2*, *MAGEA6*, *MAGEA1*, *HTR2C*, and *CTAG2*) that can be strongly associated with lower overall survival in newly diagnosed MM on RVD treatments.

In this study, we performed K-means clustering on the highly differentially expressed genes from the M20 module into two groups; C1 comprises mainly the *MAGE* genes, while C2 comprises mostly inhibitory neurotransmitter receptors. Enrichr mapped most of the genes in C1 and C2 to CD34 (+) cells that is primarily known for being a surface marker for hematopoietic stem cells (HSCs) and progenitor cells. Additionally, CD34+ is also expressed in some cancer stem cells [29]. Although, the MM stem cells remain a point of controversy. Our data suggest that CD34 expression by MM cases correlates with poor survival.

Pearson correlation between the genes in M20 shows that *MAGEA6* and *GABRA3* have a positive linear correlation of 90%. *MAGEA6* is a known targeted anti-cancer therapy for MM and has predicted poor outcomes [38]. *GABRA3*, on the other hand, is a novel gene that does not support the known biology of MM. Therefore, additional functional studies of these genes (*MAGEA6* and *GABRA3*) are needed to confirm whether they can serve as druggable targets to reverse poor outcomes in MM.

Gamma-aminobutyric acid (*GABA*) receptors, including *GABA* type A receptor alpha3 subunit (*GABRA3)*, are GPCRs and major signal transducers in the vertebrate brain. *GABRA3* may be located on the membrane of MM cells [39]. *GABA* is produced by immune cells such as CD4+, CD8+ T cells, macrophages, and dendritic cells [40]. *GABRA3* induces calcium flux via membrane depolarization [41]. *GABRA3* increases intracellular calcium signaling following *GABA* interactions with *GABRA3* [42]. Increased intracellular calcium activates cyclic AMP and *MAPK* (*ERK1/2*), leading to the eventual phosphorylation of the cAMP-responsive element-binding protein (*CREB*) [43]. The activation of *CREB* and *NF-kB* has been shown to initiate the cell growth, proliferation, survival, and progression of solid and hematogenous cancers. Ling Yan and colleagues showed that overexpression of *GABRA2*, *GABRA3*, *GABRB3*, *GABRG2*, *GABRG3*, *GABRD*, and *GABRE* might be diagnostic for colon adenocarcinoma [44]. Functional studies to elucidate the role of *GABRA3* in the GABA receptor complex in association with poor prognosis will further provide insight into the mechanisms involved.

Interestingly, *GABA* levels in cancer patients are altered due to pain management medication gabapentin, which increases *GABA* synthesis [45]. Gabapentin may have been prescribed to the MM patients of the CoMMpass study, with neuropathy caused by chemotherapies such as bortezomib [46]. Hence, we cannot exclude the possibility that GABA-modulating treatments may be upstream modulators of not only *GABRA3* but also the other functional *GABA* channel members co-expressed in MM patients. However, importantly, GABA-mediated signaling in the modulation of the immune system is well established [47], with unexplored roles in plasma (B) cell function [47]. Moreover, bioelectric conductance of drug-modifiable ion channels regulating cellular organization, including that of GABA receptors, is an emerging theme in developmental, cancer, and aging biology, with the potential to treat developmental defects, to address limited regenerative capacity, and importantly, to modify distorted signaling in cancers like MM.

Other genes from the M20 module are *MAGEA1* (which encodes melanoma-associated antigens), also from M20, are known MM signature genes [48] that have been previously shown to promote the survival of clonogenic precursors’ survival MM cells by reducing the rate of spontaneous and chemotherapy-induced apoptosis. Therefore, these genes may represent attractive targets for novel myeloma-specific therapies [6]. *CTAG2* (encodes cancer-testis antigen 2) and *MAGEA1* were shown to be expressed consistently in myeloid and round-cell liposarcomas [48]; hence, these genes are used as immunotherapy targets in the treatment of these cancers. The two remaining M20 hub genes, *SOHLH1*, a transcription factor involved in spermatogenesis and folliculogenesis [49], and *AFAP1-AS1*, have also been demonstrated to promote triple-negative breast cancer cell proliferation and invasion [50]. Of note, there is the simultaneous expression of T-cell antigens on the myeloma cells from some groups of patients with MM. These T-cell antigens expressed are CD4, CD3, and CD2 [51]. However, the CD3E in the M20 module could be as a result of impurity that was not removed.

The M13 module’s top differentially expressed genes are mostly cell cycle regulator genes: *CBX2*, *LINC00484*, *KIF7*, and *TMSB15.* To identify genes that are differentially expressed and are hub genes, for more inclusion of genes from other positively correlated modules, we set another criterion for *p*-value (<0.05) and kME (>0.7; *CENPF*, *KIF14*, *RRM2*, and *NEK2*) from the M13 module which became the top differentially expressed genes as previously described. These genes are mostly cell cycle regulators and are associated with lower overall survival in MM patients. A recent study showed that NEK2 is a serine/threonine kinase whose expression is correlated with drug resistance in MM patients [52]. However, the M10 module did not meet our criteria set for differentially expressed genes.

The M15 module is negatively correlated with MM vital status whose module members improved MM overall survival (*XXYLT1-AS2*, *LINC00996*, *KCNMB2*, *MIR320D1*, and *CHST3*). Interestingly, a previous study showed overexpression of the *XXYLT1-AS2* gene exerted a protective role against inflammatory response by blocking NF-κB activity [53]. The *XXYLT1-AS2* gene inhibited cell proliferation and migration, reduced adhesion molecule expression (VCAM-1), and restrained the adhesion of monocytes to endothelial cells [53]. *NF-κB* activity, cell to cell adhesion, and proliferation genes cause poor outcomes or lower overall survival in MM. Blocking these activities could serve as a protective mechanism and therapeutic target for promoting MM overall survival.

This study has its limitations. Firstly, it would be more reliable to validate the significance of prognostic model in real world clinical MM cohorts. Secondly, the biological functions and the underlying molecular mechanism of key hub genes in MM still need to be explored in future in vitro and in vivo studies.

## 5. Conclusions

This study utilized WGCNA to identify both known and unknown molecular signatures in newly diagnosed MM patients. The molecular mechanisms responsible for poor outcomes in MM have not been thoroughly characterized. Our study identified three networks associated with MM vital status (poor outcomes). The modules represent known and novel genes associated with MM poor outcomes and can be a resource for the MM research community. The hub genes of these modules, for example *CTAG2*, *MAGEA6*, *MAGEA1*, *GABRA3*, *HTR2C*, *NEK2*, *KIF14*, *CENPF*, and RRM2 could be explored further as new therapeutic targets or predictive clinical markers of MM outcomes.

## Figures and Tables

**Table 1:** Distribution of reported clinical traits among MM patients who received RVD therapy (some died within two years). After data normalization and outlier removal *n* = 270.

Clinical Traits	Classification	Number of Patients
Gender	Male	156/270
	Female	114/270
Race	European American	184/270
	African American	34/270
	Others	52/270
Tumor stages	I	83/270
	II	87/270
	III	93/270
	NA	7/270
MM vital status	Alive	212/270
	Dead	58/270

**Table 2:** Gene networks and their number of genes. The networks are displayed from the largest to small size of genes.

Table of Modules		
Module Name	Module Number	Number of Cluster Gene per Module
turquoise	M1	1861
blue	M2	1295
brown	M3	1274
yellow	M4	1246
green	M5	1216
red	M6	1037
black	M7	938
pink	M8	931
magenta	M9	840
purple	M10	690
greenyellow	M11	659
tan	M12	635
salmon	M13	568
cyan	M14	560
midnightblue	M15	534
lightcyan	M16	531
grey60	M17	528
lightgreen	M18	494
lightyellow	M19	435
royalblue	M20	307
darkred	M21	216

**Table 3:** ROC AUC scores and *p*-values for the top 10 genes based on their log fold change values.

Module Name	Gene	AUC	z	*p*-Value
Royalblue	CTAG2	0.64854	3.5529	3.8 × 10^−4^
	MAGEA6	0.63382	3.0658	2.17 × 10^−3^
	GABRB2	0.66859	4.19179	3.00 × 10^−5^
	SOHLH1	0.64846	3.47324	5.4 × 10^−4^
	AFAP1_AS1	0.62386	3.05481	2.30 × 10^−3^
	MAGEA1	0.62911	3.14134	1.68 × 10^−3^
	CASC9	0.61675	2.63932	8.31 × 10^−3^
	HTR2C	0.6261	2.87452	4.05 × 10^−3^
	GLDC	0.63476	3.31424	9.2 × 10^−4^
	GABRA3	0.63484	3.15689	2.60 × 10^−3^

The input genes had AUC scores above 0.6. *GABRB2* (0.669), *CTAG2* (0.649), *SOHLH1* (0.648), *GLDC* (0.635) and *GABRA3* (0.635) have the highest AUC scores.

## Data Availability

The MMRF CoMMpass datasets downloaded and analyzed for the current study are available through the TCGA portal or dbGAP (https://portal.gdc.cancer.gov/repository?facetTab=cases&filters=%7B%22op%22%3A%22and%22%2C%22content%22%3A%5B%7B%22op%22%3A%22in%22%2C%22content%22%3A%7B%22field%22%3A%22cases.project.project_id%22%2C%22value%22%3A%5B%22MMRF-COMMPASS%22%5D%7D%7D%2C%7B%22op%22%3A%22in%22%2C%22content%22%3A%7B%22field%22%3A%22files.experimental_strategy%22%2C%22value%22%3A%5B%22RNA-Seq%22%5D%7D%7D%5D%7D, accessed on 7 April 2022).

## Supplementary Materials

The following supporting information can be downloaded at: https://www.mdpi.com/article/10.3390/cancers14092228/s1, Figure S1: Enrichr Analysis of Differentially Significant Genes; Figure S2: Overall survival of the hub genes; Table S1A–C: Treatment and rate of response; Table S2: ANOVA differential analysis statistic result; Table S3: Enrichr Overlapping Genes statistical Result; Table S4: Differential and Hub Genes from positively correlated modules; Table S5A,B: Pearson correlation Analysis for Module 13 and 20; Table S6A,B: Receiver Operative Curve for validation of hub genes.
