# Supplementary material for: Multivariant Transcriptome Analysis Identifies Modules and Hub Genes Associated with Poor Outcomes in Newly Diagnosed Multiple Myeloma Patients
Source: Cancers (Basel). 2022 Apr 29;14(9):2228. doi: 10.3390/cancers14092228 (PMC9104534; doi:10.3390/cancers14092228)
Supplement: Supplementary file 1 [file cancers-14-02228-s001.zip › Figure S1.pdf]

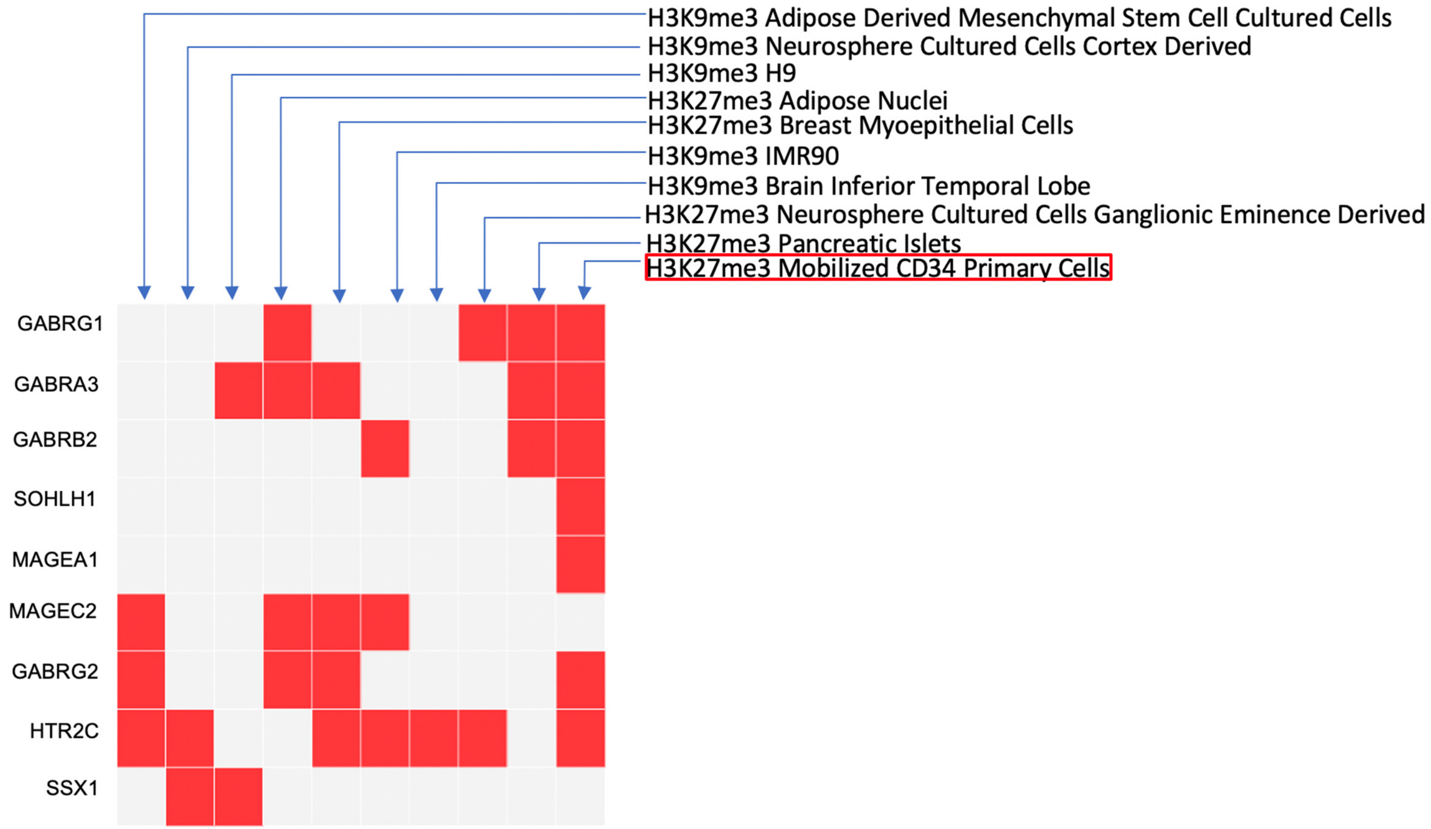


**Figure S1**. Enrichment analysis of differentially significant genes from the royalblue module. Genes with common biological enriched terms are clustered together. Terms with red box have significant fisher exact p-value.  Most of our genes were associated with H3K27me3 Mobilized CD34 Primary Cells which is known to be cancer stem cell.
