# Supplementary material for: Multivariant Transcriptome Analysis Identifies Modules and Hub Genes Associated with Poor Outcomes in Newly Diagnosed Multiple Myeloma Patients
Source: Cancers (Basel). 2022 Apr 29;14(9):2228. doi: 10.3390/cancers14092228 (PMC9104534; doi:10.3390/cancers14092228)
Supplement: Supplementary file 1 [file cancers-14-02228-s001.zip › Figure S2.pdf]

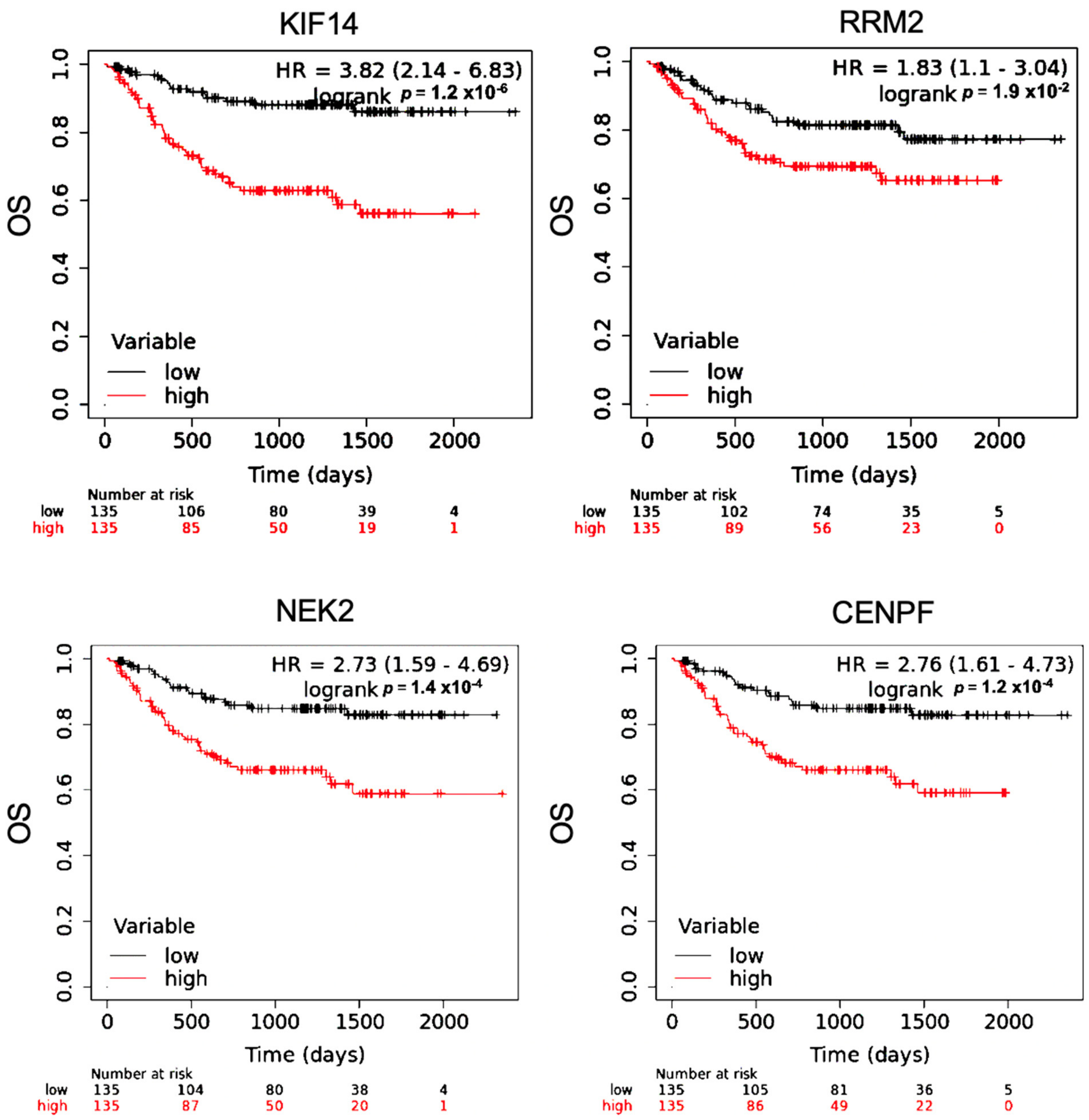

Figure S2. Overall survival of differentially expressed hub genes (p-values < 0.05, kME ≥ 0.7, and Log Fold Change (LFC) ≥ 0.5) from the salmon module (NEK2, CENPF, KIF14, and RRM2). The red line denotes samples with high gene expression and the blue line refer to samples with low expression. Pearson correlations depicts the correlation of the hub genes. Hazard ratios (HR) are reported for low expression of the genes.
