# Supplementary material for: Multivariant Transcriptome Analysis Identifies Modules and Hub Genes Associated with Poor Outcomes in Newly Diagnosed Multiple Myeloma Patients
Source: Cancers (Basel). 2022 Apr 29;14(9):2228. doi: 10.3390/cancers14092228 (PMC9104534; doi:10.3390/cancers14092228)
Supplement: Supplementary file 1 [file cancers-14-02228-s001.zip › Table S1.pdf]

Table S1A. Multiple Myeloma patients' treatment type and response tables.

Treatment Name	Number of Patients
Bortezomib	31
Bortezomib-Carfilzomib-Lenenalidomide-Cyclophosphomide-Dexamethasone	4
Bortezomib-Carfilzomib-Lenenalidomide-Dexamethasone	1
Bordezomib-Cyclophosphomide-Dexamethasone	136
Bortezomib-Dexamethasone	72
Bortezomib-Lenalidomide	4
Bortezomib-Lenalidomide-Cyclophosphomide-Dexamethasone	35
Bortezomib-Lenalidomide-Dexathmetasone	289
Bortezomib-Lenalidomide-Melphalan-Dexamethasone	0
Bortezomib-Melphalan	2
Bortezomib-Melphalan-Prednisolone	28
Bortezomib-Thalidomide-Dexamethasone	14
Carfilzomib-Cyclophosphomide-Dexamethasone	42
Carfilzomib-Dexamethasone	20
Carfilzomib-Lenalidomide	7
Carfilzomib-Lenalidomide-Cyclophosphomide-Dexamethasone	80
Carfilzomib-Lenalidomide-Dexamethasone	103
Cyclophosphomide-Dexamethasone	18
Lenalidomide	81
Lenalidomide-Clardribine-Dexamethasone	9
Lenalidomide-Dexamethasone	59

Table S1B. Number of Multiple Myeloma patient with best response

Responses	Number Patient VS Best Response
Complete Response	145
Partial Response	119
Progressive Disease	8
Stable Disease	38
Stringent Complete Response	43
Very Good Partial Response	368
N/A	47

Table S1C. Number of Multiple Myeloma patient with false response

Responses	Number Patient VS False Response
Complete Response	9
Partial Response	376
Progressive Disease	0
Stable Disease	0
Stringent Complete Response	6
Very Good Partial Response	284
N/A	93
