# Supplementary material for: Multivariant Transcriptome Analysis Identifies Modules and Hub Genes Associated with Poor Outcomes in Newly Diagnosed Multiple Myeloma Patients
Source: Cancers (Basel). 2022 Apr 29;14(9):2228. doi: 10.3390/cancers14092228 (PMC9104534; doi:10.3390/cancers14092228)
Supplement: Supplementary file 1 [file cancers-14-02228-s001.zip › Table S2.pdf]

Table. S2 Differentially expressed genes from positively and negatively correlated significant modules (M10, M13,M15 and M20) ordered by fold change values from highest to lowest.

**MM cleanDat ANOVA pairwise comparison results**		**ANOVA**		**Tukey *p* Values**	**log2(FPKM) differences**	**WCGNA**	
**UniqueID**	**ENSID**	**F-Value**	**FDR (BH)**	**Death-Alive**	**diff Death-Alive**	**NETcolors**	**Fold Change**
CTAG2	ENSG00000126890	14.6939923	0.06518483	1.58E-04	1.969112125	royalblue	3.91527088
MAGEA6	ENSG00000197172	13.5133196	0.07513653	2.86E-04	1.88310824	royalblue	3.68868921
GABRB2	ENSG00000145864	18.0265463	0.03832796	3.01E-05	1.743579786	royalblue	3.34865045
SOHLH1	ENSG00000165643	15.3802021	0.06331129	1.12E-04	1.741157299	royalblue	3.34303231
NTRK1	ENSG00000198400	29.2401024	0.00433238	1.42E-07	1.634923963	purple	3.10571181
AFAP1-AS1	ENSG00000272620	8.61147695	0.16403257	3.63E-03	1.614253562	royalblue	3.06153158
MAGEA1	ENSG00000198681	9.03799594	0.15379295	2.90E-03	1.55100075	royalblue	2.93020327
CCND2	ENSG00000118971	5.37464946	0.32312977	0.021184336	1.544469098	purple	2.91696707
CASC9	ENSG00000249395	10.0888844	0.13216723	1.67E-03	1.469280973	royalblue	2.76883863
HTR2C	ENSG00000147246	11.8591168	0.09386565	6.66E-04	1.452338658	royalblue	2.7365129
GLDC	ENSG00000178445	11.1151975	0.10359968	9.77E-04	1.429787007	royalblue	2.69406938
GABRA3	ENSG00000011677	13.4449923	0.07513653	2.96E-04	1.424029304	royalblue	2.68333895
MAGEA3	ENSG00000221867	7.17251629	0.21903534	7.86E-03	1.421920852	royalblue	2.6794202
NES	ENSG00000132688	4.91043512	0.35533606	0.027535207	1.383793001	purple	2.60953545
SLCO1A2	ENSG00000084453	12.3732287	0.08323217	5.11E-04	1.374807279	royalblue	2.59333266
BCHE	ENSG00000114200	6.00914384	0.27697497	1.49E-02	1.360671677	royalblue	2.56804713
MYO18B	ENSG00000133454	11.7433598	0.09517333	7.07E-04	1.349971765	royalblue	2.54907137
CBX2	ENSG00000173894	21.7114638	0.01529222	5.00E-06	1.337036695	salmon	2.52631878
SEMA3A	ENSG00000075213	10.8854256	0.110404	1.10E-03	1.334233499	royalblue	2.52141484
STK32A	ENSG00000169302	14.9728776	0.06518483	1.37E-04	1.322126261	royalblue	2.50034342
SEMA3D	ENSG00000153993	14.2531278	0.07215924	1.97E-04	1.307103234	royalblue	2.47444201
PKP2	ENSG00000057294	5.82230782	0.28915181	0.016495723	1.302882453	purple	2.46721331

CD3E	ENSG00000198851	10.0395157	0.1322138	1.71E-03	1.28093573	royalblue	2.42996533
PAGE1	ENSG00000068985	5.20329166	0.33528552	2.33E-02	1.270833973	royalblue	2.41301013
DPY19L2	ENSG00000177990	10.1336826	0.13036996	1.63E-03	1.270674332	royalblue	2.41274314
LINC01287	ENSG00000234722	8.00170281	0.18657923	5.03E-03	1.243924014	royalblue	2.36841848
C1orf226	ENSG00000239887	10.3334308	0.12808308	0.001466409	1.216897327	purple	2.32446278
AACSP1	ENSG00000250420	12.3702421	0.08323217	5.12E-04	1.186247681	royalblue	2.2756011
DCDC1	ENSG00000170959	18.9452947	0.03447327	1.92E-05	1.181925251	purple	2.26879342
POU6F2	ENSG00000106536	14.8486415	0.06518483	1.46E-04	1.143021578	royalblue	2.20843073
TGFB2	ENSG00000092969	7.51592064	0.205261	0.00652719	1.140399046	purple	2.20441989
HBE1	ENSG00000213931	7.22348271	0.21641921	7.65E-03	1.139142963	royalblue	2.20250144
LINC00484	ENSG00000235641	16.2865821	0.05058777	7.11E-05	1.127926714	salmon	2.18544446
FABP6	ENSG00000170231	8.25059144	0.17949484	4.40E-03	1.120300389	royalblue	2.17392232
KIF7	ENSG00000166813	17.7334367	0.0392808	3.47E-05	1.117453403	salmon	2.16963657
ADAMTS20	ENSG00000173157	10.4970771	0.12447751	1.35E-03	1.110295367	royalblue	2.15889843
CRISPLD1	ENSG00000121005	15.5875783	0.0616416	0.000100728	1.105431134	purple	2.15163168
ARHGAP28	ENSG00000088756	11.8783828	0.09386565	6.59E-04	1.103264559	royalblue	2.14840288
TRPM2-AS	ENSG00000230061	9.19678763	0.15000907	2.66E-03	1.102249798	royalblue	2.14689227
PI15	ENSG00000137558	10.0254779	0.1322138	1.72E-03	1.100399235	royalblue	2.14414019
SSX1	ENSG00000126752	5.06159901	0.34633803	2.53E-02	1.098740647	royalblue	2.1416766
TMSB15A	ENSG00000158164	7.086524	0.2232087	0.008235918	1.060926501	salmon	2.0862709
CD109	ENSG00000156535	7.5785211	0.20228779	0.006310432	1.046701378	purple	2.06580113
DDX53	ENSG00000184735	8.01071295	0.18653743	5.00E-03	1.045453642	royalblue	2.06401527
FAM3B	ENSG00000183844	6.99302374	0.22639126	8.67E-03	1.029822996	royalblue	2.04177373
TSPEAR-AS2	ENSG00000182912	8.36069559	0.17564517	4.15E-03	1.00026296	royalblue	2.00036457
PAGE5	ENSG00000158639	4.5755248	0.38046323	3.33E-02	0.998738792	royalblue	1.99825236
CHRM3	ENSG00000133019	5.0411149	0.34785755	2.56E-02	0.997917783	royalblue	1.99711552
NCALD	ENSG00000104490	8.87001082	0.1596802	0.003164407	0.997543795	purple	1.99659787
MAGEC2	ENSG00000046774	4.926251	0.35440889	2.73E-02	0.994748622	royalblue	1.99273328

MUC1	ENSG00000185499	7.13933874	0.22029895	0.008003027	0.994502389	purple	1.9923932
LINC01681	ENSG00000233985	7.93214924	0.18856183	5.22E-03	0.994272465	royalblue	1.99207569
GABRG2	ENSG00000113327	5.45085872	0.31751671	2.03E-02	0.988929902	royalblue	1.98471231
CACNA2D3	ENSG00000157445	13.3338282	0.07513653	3.13E-04	0.987051699	royalblue	1.98213016
LINC02163	ENSG00000251026	4.06246625	0.42573756	4.48E-02	0.979084408	royalblue	1.971214
NEK2	ENSG00000117650	14.8821512	0.06518483	0.000143414	0.978111463	salmon	1.96988507
TENM1	ENSG00000009694	4.85397412	0.35980363	2.84E-02	0.949153946	royalblue	1.93074006
NOS1AP	ENSG00000198929	7.74728807	0.19611824	0.005762151	0.946467841	purple	1.92714863
NELL2	ENSG00000184613	6.17870809	0.26998775	1.35E-02	0.945989795	royalblue	1.92651016
ADAMTS14	ENSG00000138316	9.5706597	0.14338504	2.19E-03	0.945784959	royalblue	1.92623665
PLAAT2	ENSG00000133328	5.98398647	0.27794837	0.015079569	0.945707033	purple	1.92613261
GABRG1	ENSG00000163285	5.9979134	0.27764471	1.50E-02	0.94313331	royalblue	1.92269951
BDKRB1	ENSG00000100739	6.37812113	0.25749474	1.21E-02	0.938779641	royalblue	1.91690606
GTSF1	ENSG00000170627	3.4971089	0.4742135	6.26E-02	0.937643398	royalblue	1.91539694
LINC01684	ENSG00000237484	7.51782536	0.205261	6.52E-03	0.929000268	royalblue	1.90395617
SPINK5	ENSG00000133710	5.95275638	0.27957208	1.53E-02	0.920148792	royalblue	1.89231045
MAGEB2	ENSG00000099399	4.62830192	0.37696325	3.23E-02	0.916660644	royalblue	1.88774075
RRM2	ENSG00000171848	15.3072578	0.06332009	0.000115887	0.916650848	salmon	1.88772793
TSPAN12	ENSG00000106025	6.50158594	0.25213718	0.011335003	0.910867078	purple	1.88017517
CENPF	ENSG00000117724	15.7973229	0.05782571	9.07E-05	0.910600406	salmon	1.87982766
LINC02444	ENSG00000258123	9.36521915	0.14585328	2.44E-03	0.90961794	royalblue	1.87854795
RNU6-583P	ENSG00000251821	5.8954236	0.28371926	0.015838946	0.908672732	salmon	1.87731759
AVPR1A	ENSG00000166148	5.52026167	0.31194726	1.95E-02	0.907287836	royalblue	1.87551635
BAGE2	ENSG00000187172	4.92683117	0.35440889	2.73E-02	0.907214642	royalblue	1.8754212
LINC01801	ENSG00000267767	6.79686759	0.2358645	9.64E-03	0.906329514	royalblue	1.87427093
KIF14	ENSG00000118193	17.1945878	0.04201811	4.53E-05	0.904552719	salmon	1.87196404
WSCD1	ENSG00000179314	2.41632932	0.57705962	0.121256085	0.904216182	purple	1.87152742
HMCN2	ENSG00000148357	3.921131	0.43729162	4.87E-02	0.891041554	royalblue	1.85451451

FAM72C	ENSG00000263513	13.782331	0.07410552	0.000249661	0.88835873	salmon	1.85106907
E2F8	ENSG00000129173	12.7834631	0.07881413	0.000414703	0.887540784	salmon	1.85001989
LINC02542	ENSG00000226453	6.10811257	0.27406192	1.41E-02	0.887455201	royalblue	1.84991015
OR56B4	ENSG00000180919	15.214924	0.06380519	1.21E-04	0.880467033	royalblue	1.84097117
EVC	ENSG00000072840	3.03125057	0.51181025	0.082822621	0.879431855	purple	1.83965069
LGALS14	ENSG00000006659	4.17744942	0.41372241	0.041942837	0.878677047	purple	1.83868845
NUF2	ENSG00000143228	19.1437184	0.03447327	1.74E-05	0.864942719	salmon	1.82126735
KIF20A	ENSG00000112984	13.0781495	0.07584465	0.000356894	0.863334375	salmon	1.8192381
INSRR	ENSG00000027644	14.436482	0.06791442	0.000179441	0.852174304	purple	1.80521955
WNT9A	ENSG00000143816	5.45115704	0.31751671	2.03E-02	0.851675929	royalblue	1.80459605
INTU	ENSG00000164066	4.60387035	0.37943977	3.28E-02	0.840563241	royalblue	1.79074913
RNA5SP323	ENSG00000212396	5.88443346	0.28465087	0.015935909	0.839726179	salmon	1.78971043
LINC00922	ENSG00000261742	4.86830802	0.35896137	2.82E-02	0.839304057	royalblue	1.78918685
MDH1B	ENSG00000138400	13.684772	0.07410552	0.000262301	0.838052378	purple	1.78763523
IQGAP3	ENSG00000183856	12.6928379	0.08162035	0.000434329	0.836077426	salmon	1.78518975
LINC02616	ENSG00000261761	4.7817274	0.36567639	2.96E-02	0.830127625	royalblue	1.77784263
LINC01518	ENSG00000233515	2.72490146	0.54539776	1.00E-01	0.825435739	royalblue	1.77207018
KIF4A	ENSG00000090889	12.9936981	0.07702631	0.00037257	0.822066046	salmon	1.767936
TSPEAR	ENSG00000175894	10.2245985	0.12940243	0.001552085	0.821832743	purple	1.76765012
DRP2	ENSG00000102385	12.2290967	0.08558391	0.000550571	0.821080752	salmon	1.76672899
TLCD3B	ENSG00000149926	13.7418667	0.07410552	0.000254827	0.814858906	salmon	1.7591261
E2F2	ENSG00000007968	14.1666358	0.07400801	0.000205591	0.812478829	salmon	1.75622639
LINC00958	ENSG00000251381	3.82625556	0.44471242	5.15E-02	0.811384275	royalblue	1.75489447
RNF2P1	ENSG00000231381	9.20768659	0.14946792	2.65E-03	0.809141159	royalblue	1.75216806
FAM72D	ENSG00000215784	16.6585933	0.04546232	5.91E-05	0.807384339	salmon	1.75003568
TICRR	ENSG00000140534	13.1211535	0.07577244	0.00034917	0.806559783	salmon	1.74903576
SKA1	ENSG00000154839	16.0748453	0.05371157	7.90E-05	0.803547089	salmon	1.74538716
LRP2	ENSG00000081479	7.67908342	0.19645782	0.005977588	0.802960308	purple	1.74467741

CROCC2	ENSG00000226321	6.13134785	0.27248632	1.39E-02	0.797787291	royalblue	1.73843279
LINC01234	ENSG00000249550	5.99311432	0.27764471	1.50E-02	0.796657068	royalblue	1.73707142
C3orf67	ENSG00000163689	6.78128352	0.23731303	9.73E-03	0.79480269	royalblue	1.73484009
VN1R76P	ENSG00000273762	3.56734859	0.46826473	0.060006079	0.791900261	purple	1.73135343
MKRN9P	ENSG00000258128	6.04133613	0.27608214	1.46E-02	0.791132146	royalblue	1.73043188
PRR11	ENSG00000068489	16.9268204	0.04523111	5.17E-05	0.790283622	salmon	1.72941442
KIF23	ENSG00000137807	12.4563387	0.08323217	0.000490111	0.78426853	salmon	1.72221891
ADGRL3	ENSG00000150471	3.42484011	0.48151005	6.53E-02	0.783566144	royalblue	1.72138064
CYP21A1P	ENSG00000204338	4.08290963	0.42345518	0.044313468	0.783430317	purple	1.72121858
ANKFN1	ENSG00000153930	10.4024652	0.12805696	1.41E-03	0.780919653	royalblue	1.71822582
FAM238B	ENSG00000231976	5.07149734	0.34561474	2.51E-02	0.780793527	royalblue	1.71807561
PKHD1	ENSG00000170927	3.53493359	0.4709717	6.12E-02	0.780591399	royalblue	1.71783492
KIF18B	ENSG00000186185	12.5333335	0.08323217	0.000471193	0.778495468	salmon	1.71534108
C12orf75	ENSG00000235162	6.94286786	0.2284458	0.008905351	0.777130243	salmon	1.71371861
HJURP	ENSG00000123485	11.5753502	0.09780062	0.00077037	0.775155854	salmon	1.71137492
DUXAP8	ENSG00000206195	3.30868662	0.48966923	7.00E-02	0.774116244	royalblue	1.71014214
PTGFR	ENSG00000122420	3.96853251	0.43297298	4.74E-02	0.772809791	royalblue	1.7085942
TRPC4	ENSG00000133107	2.85557694	0.52980847	9.22E-02	0.771283916	royalblue	1.70678805
EXO1	ENSG00000174371	14.6944594	0.06518483	0.000157596	0.771236902	salmon	1.70673243
FAM83D	ENSG00000101447	13.6790869	0.07410552	0.000263058	0.763929904	salmon	1.69810999
MKI67	ENSG00000148773	10.157526	0.13036996	0.001607412	0.760526582	salmon	1.69410886
EVC2	ENSG00000173040	2.67166879	0.55097149	0.10332291	0.757067732	purple	1.69005211
SPC25	ENSG00000152253	11.2239347	0.10214637	0.000923571	0.751818956	salmon	1.68391458
XAGE3	ENSG00000171402	2.58981395	0.55860094	1.09E-01	0.749892296	royalblue	1.68166728
CCDC150	ENSG00000144395	14.9792889	0.06518483	0.00013659	0.74837964	salmon	1.67990499
SUN3	ENSG00000164744	8.91426032	0.15895934	0.003091078	0.747534651	purple	1.67892135
MELK	ENSG00000165304	14.7316138	0.06518483	0.00015468	0.746488986	salmon	1.67770491
ASPM	ENSG00000066279	12.3860091	0.08323217	0.000508065	0.740745814	salmon	1.67103947

SEPTIN14P12	ENSG00000235748	9.25104669	0.14823117	0.002586948	0.737201765	salmon	1.66693952
SCN9A	ENSG00000169432	4.19723979	0.41098438	0.041463782	0.730436105	purple	1.65914055
ARC	ENSG00000198576	4.50896217	0.3872477	3.46E-02	0.729625156	royalblue	1.6582082
DCC	ENSG00000187323	2.43564903	0.57411919	0.119784853	0.727847006	purple	1.65616568
TPX2	ENSG00000088325	11.4599708	0.09972885	0.000817588	0.727315947	salmon	1.65555616
MIR4435-2HG	ENSG00000172965	8.77909953	0.16182112	0.003320697	0.723260605	salmon	1.65090901
FAM72A	ENSG00000196550	17.5238792	0.0392808	3.85E-05	0.722080482	salmon	1.64955912
TNXA	ENSG00000248290	4.70297082	0.37243719	0.030990205	0.72126602	purple	1.64862813
TOP2A	ENSG00000131747	8.79421101	0.16182112	0.003294181	0.719746988	salmon	1.64689319
BUB1B	ENSG00000156970	12.4964363	0.08323217	0.000480164	0.716895295	salmon	1.64364108
DTL	ENSG00000143476	12.3925696	0.08323217	0.000506362	0.71669502	salmon	1.64341293
HECW2	ENSG00000138411	7.08182308	0.22323356	8.26E-03	0.716414811	royalblue	1.64309376
CENPI	ENSG00000102384	10.526273	0.12297165	0.001326251	0.714913836	salmon	1.64138518
CDCA8	ENSG00000134690	15.1878143	0.06380519	0.000123031	0.714808859	salmon	1.64126575
PURPL	ENSG00000250337	5.29556354	0.32800927	2.21E-02	0.710644977	royalblue	1.63653559
STEAP1	ENSG00000164647	3.35247881	0.48566885	0.068214101	0.70938613	purple	1.63510823
CALHM3	ENSG00000183128	5.39771104	0.32202262	0.02091179	0.709274998	purple	1.63498228
CENPE	ENSG00000138778	14.0372848	0.07410552	0.000219466	0.708847954	salmon	1.63449839
WEE1	ENSG00000166483	7.76203613	0.19522017	0.005716624	0.708476311	salmon	1.63407739
LIPH	ENSG00000163898	3.23183422	0.49466258	0.073345278	0.70742346	purple	1.63288531
FAM111B	ENSG00000189057	9.88513972	0.13477394	0.001853585	0.705672931	salmon	1.63090521
TROAP	ENSG00000135451	11.3130191	0.10132658	0.000882019	0.702402333	salmon	1.62721212
POLD2P1	ENSG00000213730	7.62302661	0.19937718	6.16E-03	0.700611874	royalblue	1.62519392
CCNB2	ENSG00000157456	11.4122997	0.10065508	0.000837947	0.696444385	salmon	1.62050603
DNAH14	ENSG00000185842	3.85092582	0.44332724	0.050753098	0.69166422	purple	1.6151456
ELFN1-AS1	ENSG00000236081	2.01771724	0.61809639	1.57E-01	0.691104063	royalblue	1.6145186
UBE2C	ENSG00000175063	11.2647405	0.10214637	0.000904296	0.690420614	salmon	1.61375394
CCNA2	ENSG00000145386	14.6671202	0.06518483	0.000159777	0.687605571	salmon	1.61060819

LINC01202	ENSG00000280776	3.66159216	0.4610232	5.67E-02	0.685413673	royalblue	1.60816303
TRIM31	ENSG00000204616	4.67631578	0.37370286	0.031465997	0.680671174	purple	1.60288528
11-Mar	ENSG00000183654	5.20903396	0.33451581	2.33E-02	0.680470075	royalblue	1.60266187
CEP55	ENSG00000138180	9.32190605	0.14607658	0.002492057	0.680465418	salmon	1.60265669
DSCC1	ENSG00000136982	12.2014702	0.08629569	0.00055842	0.679643015	salmon	1.60174337
STARD6	ENSG00000174448	11.5674657	0.09780062	0.000773506	0.678752057	purple	1.60075449
NME5	ENSG00000112981	6.13926802	0.27193999	0.013837851	0.678040995	purple	1.59996572
KIF2C	ENSG00000142945	13.2159785	0.07513653	0.000332734	0.677494141	salmon	1.59935937
LINC02477	ENSG00000249425	4.28937629	0.40360227	3.93E-02	0.676767271	royalblue	1.59855377
ESPL1	ENSG00000135476	14.6034665	0.06641984	0.000164975	0.675697525	salmon	1.59736889
CNBD1	ENSG00000176571	5.70272684	0.29658473	1.76E-02	0.675134731	royalblue	1.59674588
SPC24	ENSG00000161888	10.2020122	0.12973798	0.001570496	0.673053043	salmon	1.59444357
UBE2T	ENSG00000077152	12.8497965	0.07841223	0.000400911	0.672001276	salmon	1.5932816
NUSAP1	ENSG00000137804	12.8057045	0.07841223	0.000410025	0.6701918	salmon	1.59128451
GTSE1	ENSG00000075218	13.01499	0.07671416	0.000368553	0.669530754	salmon	1.59055554
ESCO2	ENSG00000171320	10.5702356	0.12129327	0.001296258	0.667945785	salmon	1.58880909
CLSTN2	ENSG00000158258	1.86648656	0.63716042	0.173023268	0.665152375	purple	1.58573575
LINC02466	ENSG00000246876	3.26905661	0.49256303	7.17E-02	0.664690818	royalblue	1.58522851
ZNF112	ENSG00000062370	4.31851992	0.40158156	3.87E-02	0.659131967	royalblue	1.57913221
DLGAP5	ENSG00000126787	8.3933944	0.17520046	0.004076826	0.658283313	salmon	1.57820358
ASTN1	ENSG00000152092	2.29758228	0.59112937	1.31E-01	0.654680935	royalblue	1.57426775
ZNF300	ENSG00000145908	3.61018813	0.46432664	5.85E-02	0.650077914	royalblue	1.56925294
BIRC5	ENSG00000089685	9.68349597	0.14072336	0.002060352	0.647412205	salmon	1.56635607
TTK	ENSG00000112742	10.790256	0.11412167	0.00115621	0.645133689	salmon	1.5638842
PHF19	ENSG00000119403	11.5059781	0.09811222	0.000798416	0.642041437	salmon	1.56053578
OVAAL	ENSG00000236719	2.23510702	0.59710429	1.36E-01	0.639968177	royalblue	1.55829479
NANOS1	ENSG00000188613	3.43810227	0.48061954	0.064807356	0.638282561	purple	1.55647517
HIST1H3G	ENSG00000273983	11.3612643	0.10132658	0.000860315	0.637015523	salmon	1.5551088

CENPA	ENSG00000115163	9.21707316	0.14946792	0.002633746	0.634897474	salmon	1.55282739
HMGB3	ENSG00000029993	9.32676899	0.14598224	0.002485677	0.633955504	salmon	1.55181385
TSSC2	ENSG00000223756	2.31927	0.58960117	1.29E-01	0.632197895	royalblue	1.54992445
CCDC192	ENSG00000230561	8.8725752	0.1596802	3.16E-03	0.632034659	royalblue	1.54974909
TYMS	ENSG00000176890	12.5464162	0.08323217	0.000468053	0.630536229	salmon	1.54814031
PKMYT1	ENSG00000127564	11.258347	0.10214637	0.000907289	0.629801598	salmon	1.54735218
NALCN-AS1	ENSG00000233009	5.02932236	0.34808795	2.57E-02	0.629202524	royalblue	1.54670979
MCM10	ENSG00000065328	10.4629707	0.1255697	0.001370687	0.626612581	salmon	1.54393561
CLSPN	ENSG00000092853	12.5564842	0.08323217	0.000465651	0.625063098	salmon	1.54227828
DIAPH3	ENSG00000139734	9.1113626	0.15297416	0.002784968	0.624999998	salmon	1.54221082
ANLN	ENSG00000011426	9.25788222	0.14823117	0.002577636	0.624665774	salmon	1.54185359
ZWINT	ENSG00000122952	11.0145711	0.10536653	0.001029252	0.624085035	salmon	1.54123306
TSPEAR-AS1	ENSG00000235890	3.72772816	0.45398241	5.46E-02	0.620259359	royalblue	1.5371515
FOXM1	ENSG00000111206	11.4587781	0.09972885	0.000818091	0.617435076	salmon	1.53414525
KIFC1	ENSG00000237649	10.6734422	0.11799054	0.001228531	0.615256775	salmon	1.53183061
GPSM1	ENSG00000160360	4.4041939	0.39508432	3.68E-02	0.614683285	royalblue	1.53122181
CPA5	ENSG00000158525	4.05688925	0.4259788	4.50E-02	0.613760588	royalblue	1.53024281
NCAPH	ENSG00000121152	11.5130843	0.09811222	0.000795496	0.608962799	salmon	1.52516233
CIT	ENSG00000122966	8.89257338	0.1596802	0.003126797	0.607309996	salmon	1.52341605
KLK2	ENSG00000167751	3.38896933	0.4848947	6.67E-02	0.603563514	royalblue	1.51946507
SCN3A	ENSG00000153253	1.55124054	0.68149868	2.14E-01	0.601191704	royalblue	1.51696911
FAM72B	ENSG00000188610	11.2592861	0.10214637	0.000906849	0.600864103	salmon	1.51662468
DEPDC1	ENSG00000024526	7.00760824	0.22577747	0.008596984	0.598112974	salmon	1.51373533
CDCA5	ENSG00000146670	10.9996435	0.10579175	0.001037242	0.595580643	salmon	1.51108063
GHET1	ENSG00000281189	8.78040891	0.16182112	0.00331839	0.595354532	purple	1.51084382
ANKRD45	ENSG00000183831	1.98978934	0.6208715	1.60E-01	0.59383409	royalblue	1.50925239
CDK1	ENSG00000170312	7.28240164	0.21356959	0.007405626	0.593272144	salmon	1.50866464
IL6R	ENSG00000160712	7.06740862	0.22336871	0.008321919	0.592039393	purple	1.50737607

AURKB	ENSG00000178999	10.1885801	0.12973798	0.00158155	0.591134913	salmon	1.50643133
NEURL1B	ENSG00000214357	3.54016653	0.47045154	0.060983253	0.590559478	salmon	1.5058306
NDC80	ENSG00000080986	9.88756735	0.13477394	0.001851229	0.589873764	salmon	1.50511504
ARMH4	ENSG00000139971	4.28824507	0.40360321	3.93E-02	0.588040731	royalblue	1.50320392
NCAPG	ENSG00000109805	12.6847575	0.08162035	0.000436124	0.587526668	salmon	1.50266839
GINS1	ENSG00000101003	9.82473744	0.13582378	0.001913199	0.5856246	salmon	1.50068856
DRAIC	ENSG00000245750	6.83361038	0.2333461	9.45E-03	0.585330068	royalblue	1.50038222
PPP1R1C	ENSG00000150722	3.73428035	0.45366765	5.44E-02	0.584281965	royalblue	1.4992926
SPA17	ENSG00000064199	8.65899068	0.16386161	0.003539373	0.581857817	purple	1.49677547
LILRB4	ENSG00000186818	1.40521225	0.70096324	2.37E-01	0.58169319	royalblue	1.49660468
CDC20	ENSG00000117399	9.07121233	0.15297416	0.002844705	0.578452121	salmon	1.49324627
SLC1A1	ENSG00000106688	8.07328389	0.1845533	4.84E-03	0.577961638	royalblue	1.49273869
KIF18A	ENSG00000121621	11.5408175	0.09793903	0.000784204	0.576601042	salmon	1.49133156
CRMP1	ENSG00000072832	1.95985966	0.62363212	0.162684867	0.573911274	purple	1.48855371
NPIPB2	ENSG00000234719	8.29516409	0.17788172	0.004296183	0.57286078	purple	1.48747022
CCNF	ENSG00000162063	16.5023366	0.04742106	6.39E-05	0.57133066	salmon	1.48589344
KIAA0319	ENSG00000137261	9.27115683	0.14805153	0.002559649	0.568975067	purple	1.4834693
POLQ	ENSG00000051341	12.308193	0.08425772	0.00052871	0.567827204	salmon	1.48228946
SKA3	ENSG00000165480	7.64127489	0.19831435	0.00610056	0.567089852	salmon	1.48153207
AURKA	ENSG00000087586	8.19516518	0.18123823	0.004531932	0.564817504	salmon	1.47920038
PLK1	ENSG00000166851	11.1961017	0.10214637	0.000936958	0.56470564	salmon	1.47908569
SHANK2	ENSG00000162105	2.12678731	0.60579669	0.145914761	0.562414837	purple	1.47673897
CCNB1	ENSG00000134057	10.9802272	0.10639852	0.001047729	0.561641372	salmon	1.47594747
SPATA17	ENSG00000162814	4.65348517	0.37546287	0.031879618	0.559200612	purple	1.47345256
STMN1	ENSG00000117632	10.7503654	0.11576303	0.001180406	0.559034518	salmon	1.47328294
BUB1	ENSG00000169679	11.6224631	0.09664159	0.000751896	0.556628476	salmon	1.47082793
DPY19L2P2	ENSG00000170629	3.8252996	0.44471242	0.051523157	0.556264133	salmon	1.47045653
LINC02321	ENSG00000258884	3.59316228	0.46529741	0.059093593	0.556046821	salmon	1.47023505

CKAP2L	ENSG00000169607	6.66433009	0.24238234	0.010367855	0.555837056	salmon	1.4700213
DEPDC1B	ENSG00000035499	8.78358511	0.16182112	0.003312803	0.554403179	salmon	1.46856099
LYPD8	ENSG00000259823	2.78282966	0.53980305	9.64E-02	0.553277785	royalblue	1.46741586
NEIL3	ENSG00000109674	6.90412358	0.22931255	0.009095325	0.55282677	salmon	1.46695719
TMEM132D-AS1	ENSG00000249196	1.20742917	0.72872305	2.73E-01	0.551756757	royalblue	1.46586959
SMPX	ENSG00000091482	1.76839787	0.65146764	1.85E-01	0.551437328	royalblue	1.46554506
ST5	ENSG00000166444	2.53107219	0.56441707	1.13E-01	0.550736855	royalblue	1.46483367
ACRV1	ENSG00000134940	9.35665073	0.14592516	0.002446836	0.54836962	salmon	1.46243208
SERPINH1P1	ENSG00000229207	10.3602882	0.12808308	0.001446018	0.547900445	salmon	1.46195656
LINC00896	ENSG00000236499	2.25701466	0.59484754	1.34E-01	0.547805812	royalblue	1.46186067
LINC02241	ENSG00000251629	1.99651218	0.620491	1.59E-01	0.547581255	royalblue	1.46163315
VENTXP5	ENSG00000253569	3.14505372	0.50152637	7.73E-02	0.547486951	royalblue	1.46153761
TMPOP2	ENSG00000262904	9.59017226	0.14331226	0.002163876	0.546217653	salmon	1.4602523
CYCSP6	ENSG00000214429	1.65380017	0.66551967	2.00E-01	0.545383775	royalblue	1.45940851
FAM227A	ENSG00000184949	4.38976398	0.39616628	0.037093899	0.544690884	purple	1.45870776
PBK	ENSG00000168078	5.96433129	0.27915004	0.015244778	0.543466123	salmon	1.45746993
NOSTRIN	ENSG00000163072	6.00423844	0.27702213	0.014911283	0.543219784	salmon	1.45722109
ATP2A1-AS1	ENSG00000260442	11.6952289	0.09593262	0.000724245	0.5426505	salmon	1.45664619
ATP2B4	ENSG00000058668	6.91668786	0.22892563	0.009033266	0.537415919	purple	1.45137057
SOX1-OT	ENSG00000224243	1.66826527	0.66327512	1.98E-01	0.536856159	royalblue	1.45080755
TRIP13	ENSG00000071539	9.57743015	0.14338504	0.002178417	0.536326965	salmon	1.45027548
HIST2H4A	ENSG00000270882	8.00918958	0.18653743	0.005006275	0.536257087	purple	1.45020524
MAGEC1	ENSG00000155495	1.07333002	0.74908004	3.01E-01	0.533686955	royalblue	1.44762402
EEF1E1P1	ENSG00000236307	5.59589002	0.30566205	0.018714871	0.533624141	purple	1.447561
KNL1	ENSG00000137812	9.47206975	0.14527608	0.002302543	0.53266049	salmon	1.44659442
CEACAM16	ENSG00000213892	2.09668459	0.60884136	1.49E-01	0.531695003	royalblue	1.44562665
CDC45	ENSG00000093009	8.49167915	0.17032814	0.003868817	0.530807398	salmon	1.44473751
CSMD1	ENSG00000183117	2.49980103	0.56618304	1.15E-01	0.529944735	royalblue	1.44387388

KIF11	ENSG00000138160	8.29235773	0.17790769	0.004302624	0.529361025	salmon	1.44328982
CDCA2	ENSG00000184661	6.01737743	0.27677597	0.014803146	0.526455943	salmon	1.44038646
PCLAF	ENSG00000166803	9.43100801	0.14573404	0.002352854	0.524001697	salmon	1.43793823
CCDC138	ENSG00000163006	18.1862494	0.03832796	2.78E-05	0.522997964	salmon	1.43693815
NPHP1	ENSG00000144061	4.87631381	0.35837624	0.028074477	0.522276016	purple	1.43621926
POT1-AS1	ENSG00000224897	3.82302234	0.44493171	5.16E-02	0.520297846	royalblue	1.43425132
FAM86GP	ENSG00000166492	3.53190834	0.47149919	6.13E-02	0.517785609	royalblue	1.43175596
ZNF284	ENSG00000186026	4.2494588	0.40755554	0.040227287	0.517681031	purple	1.43165218
SHCBP1	ENSG00000171241	7.06787089	0.22336871	0.008319828	0.517527324	salmon	1.43149966
PLCB1	ENSG00000182621	1.88521555	0.63402905	1.71E-01	0.517008492	royalblue	1.43098494
NDN	ENSG00000182636	1.39908586	0.70125419	2.38E-01	0.515821238	royalblue	1.42980781
DMC1	ENSG00000100206	3.82557088	0.44471242	0.05151494	0.510433966	salmon	1.42447862
SVOP	ENSG00000166111	1.48231848	0.69122869	2.24E-01	0.510295909	royalblue	1.42434231
TEX41	ENSG00000226674	1.4895735	0.69100788	2.23E-01	0.509824937	royalblue	1.42387741
KIF15	ENSG00000163808	8.85187091	0.16026226	0.003194979	0.508002942	salmon	1.42208031
SHTN1	ENSG00000187164	2.07223122	0.61198526	1.51E-01	0.507910026	royalblue	1.42198873
TYMSOS	ENSG00000176912	5.01008576	0.34882766	0.026021446	0.506496166	salmon	1.42059584
VN1R54P	ENSG00000232109	1.97246723	0.62250607	1.61E-01	0.504570787	royalblue	1.41870122
FHAD1	ENSG00000142621	1.94874014	0.6252294	1.64E-01	0.502578047	royalblue	1.41674297
HMMR	ENSG00000072571	9.44163741	0.14573404	0.002339723	0.50192663	salmon	1.41610342
EFL1P1	ENSG00000259404	3.31767166	0.48964541	6.97E-02	0.499899257	royalblue	1.41411481
KLRG1	ENSG00000139187	6.55881603	0.24915483	0.010984701	0.498528552	purple	1.4127719
UHRF1	ENSG00000276043	7.0586124	0.22364901	0.008361804	0.49849221	salmon	1.41273631
CDC25C	ENSG00000158402	9.46493004	0.1455112	0.002311211	0.497990499	salmon	1.4122451
MYLK2	ENSG00000101306	8.13917432	0.18428839	0.004669683	0.495367019	salmon	1.40967933
CDKN3	ENSG00000100526	10.6639601	0.11799054	0.0012346	0.491162886	salmon	1.40557739
ACOT7	ENSG00000097021	14.069514	0.07410552	0.000215922	0.490097368	salmon	1.40453967
FSD2	ENSG00000186628	5.08040259	0.34474531	2.50E-02	0.488391914	royalblue	1.4028803

SPAG5	ENSG00000076382	12.3822098	0.08323217	0.000509053	0.487031282	salmon	1.40155784
CSPG4	ENSG00000173546	3.79609279	0.44824613	5.24E-02	0.484646756	royalblue	1.39924322
LINC02714	ENSG00000251226	2.2701387	0.59374223	1.33E-01	0.484237729	royalblue	1.39884657
RMI2	ENSG00000175643	4.92277943	0.35440889	0.027342791	0.483549936	salmon	1.39817984
TNFRSF8	ENSG00000120949	2.34796906	0.5858914	1.27E-01	0.481799896	royalblue	1.39648482
MTX1P1	ENSG00000236675	13.3056728	0.07513653	0.00031791	0.480402159	purple	1.39513251
PLK4	ENSG00000142731	8.2293826	0.1796277	0.004449807	0.480083244	salmon	1.39482415
ICAM5	ENSG00000105376	1.82402225	0.6432221	1.78E-01	0.478749561	royalblue	1.39353531
CENPW	ENSG00000203760	11.7835122	0.09517333	0.00069208	0.475363214	salmon	1.39026819
CCDC15	ENSG00000149548	4.84293356	0.36073043	0.028612745	0.473820053	salmon	1.3887819
RPRM	ENSG00000177519	1.09162189	0.74610765	0.297053598	0.473261846	purple	1.38824466
REEP1	ENSG00000068615	3.06558613	0.50794465	8.11E-02	0.471301036	royalblue	1.38635914
ST8SIA6-AS1	ENSG00000204832	1.03760424	0.75511846	3.09E-01	0.467543307	royalblue	1.38275284
DHFRP1	ENSG00000188985	5.24307965	0.33111562	0.022811678	0.466808124	salmon	1.38204838
PGAP1	ENSG00000197121	5.36441758	0.32370133	0.021306441	0.466314061	purple	1.38157517
WDR62	ENSG00000075702	7.96886679	0.18741778	0.00511567	0.465866441	salmon	1.38114658
RAB36	ENSG00000100228	3.91258075	0.43791485	0.048950082	0.464449755	purple	1.379791
BMS1P22	ENSG00000232775	2.24959661	0.59573588	1.35E-01	0.464050369	royalblue	1.37940908
DNA2	ENSG00000138346	17.7365972	0.0392808	3.47E-05	0.463820797	salmon	1.3791896
RACGAP1	ENSG00000161800	10.0651415	0.1322138	0.001686929	0.463454214	salmon	1.37883919
TEDC2	ENSG00000162062	6.86588836	0.23167073	0.0092869	0.460077	salmon	1.37561524
GINS3	ENSG00000181938	8.04063196	0.18549618	0.004922639	0.458883083	salmon	1.3744773
TK1	ENSG00000167900	6.80365492	0.23555701	0.009607659	0.458560585	salmon	1.37417009
LINC02830	ENSG00000224899	1.28891463	0.71610189	2.57E-01	0.45742398	royalblue	1.3730879
RN7SL219P	ENSG00000265386	2.4709844	0.57010299	0.117145091	0.457048476	purple	1.37273056
SGO2	ENSG00000163535	8.7931097	0.16182112	0.003296106	0.455532301	salmon	1.37128867
RAD54L	ENSG00000085999	8.75476461	0.16256807	0.003363857	0.455273953	salmon	1.37104313
FBXO43	ENSG00000156509	5.07192396	0.34561474	0.025126061	0.454403511	salmon	1.37021617

ORC1	ENSG00000085840	9.50591851	0.14527608	0.002261897	0.454271032	salmon	1.37009035
LINC01515	ENSG00000228065	2.23320989	0.59728897	1.36E-01	0.454207938	royalblue	1.37003043
HSD17B6	ENSG00000025423	6.49148933	0.25235582	0.011397992	0.454020274	purple	1.36985223
MCM2	ENSG00000073111	7.64475213	0.19831435	0.006089142	0.453216742	salmon	1.36908948
DEPDC1-AS1	ENSG00000234264	6.16296094	0.2703136	0.013657913	0.45255965	salmon	1.36846606
STIL	ENSG00000123473	11.6654202	0.09616727	0.000735445	0.449392122	salmon	1.3654648
MND1	ENSG00000121211	6.19027508	0.26998775	0.013453475	0.448406309	salmon	1.36453208
CDKN2C	ENSG00000123080	3.62680666	0.4624797	0.057926507	0.447535331	salmon	1.36370854
SHISA4	ENSG00000198892	6.14317545	0.27164468	0.013808007	0.446878595	purple	1.3630879
DYRK3	ENSG00000143479	8.69547151	0.16304475	3.47E-03	0.4466009	royalblue	1.36282555
E2F1	ENSG00000101412	6.3882772	0.25721551	0.012063019	0.445973205	salmon	1.36223273
ATAD2	ENSG00000156802	9.45543383	0.14564098	0.002322792	0.44585362	salmon	1.36211982
RAB29	ENSG00000117280	3.07651688	0.50665578	0.080573469	0.445625128	purple	1.36190411
C18orf54	ENSG00000166845	5.60975699	0.30451123	0.018570428	0.444927228	salmon	1.36124545
FANCI	ENSG00000140525	10.0035981	0.1322138	0.001742117	0.444775488	salmon	1.36110229
TMPO-AS1	ENSG00000257167	9.93530909	0.13409008	0.001805514	0.444565422	salmon	1.36090411
ZNF229	ENSG00000278318	1.29651918	0.71605378	2.56E-01	0.442089836	royalblue	1.35857088
CCDC34	ENSG00000109881	7.31478262	0.21310928	0.007276897	0.437870008	salmon	1.35460292
RAD51	ENSG00000051180	9.33177715	0.14598224	0.002479124	0.437698902	salmon	1.35444227
STRIP2	ENSG00000128578	5.03083366	0.34808795	0.025717367	0.436647256	salmon	1.35345531
PAQR4	ENSG00000162073	5.39116866	0.32255818	0.020988736	0.43386417	salmon	1.3508469
CHML	ENSG00000203668	9.9305412	0.13409979	0.001810027	0.433120265	purple	1.35015053
LMNB1	ENSG00000113368	14.6934389	0.06518483	0.000157677	0.432486052	salmon	1.34955713
PTTG1	ENSG00000164611	5.92409718	0.28131258	0.015588839	0.431482718	salmon	1.3486189
SHOX2	ENSG00000168779	1.9551115	0.62435929	1.63E-01	0.431065335	royalblue	1.34822879
SDK2	ENSG00000069188	1.08246693	0.74729622	2.99E-01	0.430532882	royalblue	1.34773129
FAM133A	ENSG00000179083	1.21407951	0.72716544	2.72E-01	0.43007688	royalblue	1.34730537
ACSM4	ENSG00000215009	1.7131371	0.6574677	1.92E-01	0.429463297	royalblue	1.34673248

GBAP1	ENSG00000160766	9.37496744	0.14585328	0.002423335	0.427223512	purple	1.3446433
S100A5	ENSG00000196420	3.87997718	0.44123812	0.049894891	0.426943354	purple	1.34438221
HEATR6	ENSG00000068097	22.173851	0.01529222	4.00E-06	0.426433452	salmon	1.34390714
GLYATL1B	ENSG00000255151	3.31744918	0.48964541	0.069662907	0.424197478	salmon	1.34182588
ARHGAP11A	ENSG00000198826	6.16991043	0.27015185	0.013605595	0.423583602	salmon	1.34125505
KIF24	ENSG00000186638	5.61818442	0.30401069	0.018483211	0.423565927	salmon	1.34123862
RAD51AP1	ENSG00000111247	8.34908669	0.17593058	0.004174312	0.423387377	salmon	1.34107263
GINS4	ENSG00000147536	5.91940345	0.28131258	0.015629498	0.422628922	salmon	1.34036779
WDHD1	ENSG00000198554	5.79639926	0.29044979	0.016735178	0.420062801	salmon	1.3379858
KCTD3	ENSG00000136636	12.6366519	0.08288676	0.000446968	0.419328638	purple	1.33730509
NCAPG2	ENSG00000146918	7.97605601	0.18741778	0.005095987	0.419076235	salmon	1.33707115
LCLAT1	ENSG00000172954	15.2631524	0.06359822	0.000118475	0.418836807	salmon	1.33684927
HASPIN	ENSG00000177602	4.52886075	0.3853206	0.034240366	0.417422077	salmon	1.33553897
CDT1	ENSG00000167513	5.4650692	0.31683656	0.020136448	0.416728028	salmon	1.33489663
IL6R-AS1	ENSG00000228013	4.98794407	0.35070048	0.026350101	0.415596229	purple	1.33384981
MAD2L1	ENSG00000164109	9.20712534	0.14946792	0.002647611	0.41493993	salmon	1.33324316
ELFN1	ENSG00000225968	1.41197221	0.69997255	2.36E-01	0.412307086	royalblue	1.33081228
ABCA12	ENSG00000144452	4.52974001	0.38526778	0.034223081	0.411221828	salmon	1.32981156
NSD2	ENSG00000109685	2.79413781	0.53916876	0.095776803	0.410168013	salmon	1.32884056
SPARCL1	ENSG00000152583	1.34460551	0.70916898	2.47E-01	0.409712317	royalblue	1.32842089
CSAG1	ENSG00000198930	0.84303111	0.78949154	3.59E-01	0.409561893	royalblue	1.32828239
PSRC1	ENSG00000134222	4.44078187	0.39285142	0.03601925	0.408602682	salmon	1.32739954
TTLL9	ENSG00000131044	4.29328646	0.40323919	3.92E-02	0.407515631	royalblue	1.32639974
LINC02382	ENSG00000250968	0.95882899	0.76879724	3.28E-01	0.407267677	royalblue	1.3261718
HELLS	ENSG00000119969	7.6159937	0.19950518	0.006184233	0.402907177	salmon	1.32216953
PLS1	ENSG00000120756	3.96591481	0.43297298	0.047445261	0.401551893	purple	1.32092806
GGH	ENSG00000137563	5.18969478	0.33653413	0.023508673	0.400460684	salmon	1.31992933
BRIP1	ENSG00000136492	5.35908194	0.32435103	0.021370405	0.400169457	salmon	1.31966291

LINC01137	ENSG00000233621	5.48950268	0.31539042	0.019862649	0.399835117	purple	1.31935712
LINC01572	ENSG00000261008	3.52193671	0.47283698	0.061648135	0.399237116	salmon	1.31881035
SYCE3	ENSG00000217442	2.51418698	0.56546038	0.114005003	0.398864587	purple	1.31846986
HNRNPA1P8	ENSG00000229251	1.42331574	0.69892805	2.34E-01	0.398511534	royalblue	1.31814724
FSTL5	ENSG00000168843	0.76491461	0.8028239	3.83E-01	0.398416518	royalblue	1.31806043
ECT2	ENSG00000114346	10.337893	0.12808308	0.001463001	0.397339709	salmon	1.31707702
SGO1	ENSG00000129810	5.73790157	0.29455171	0.017289198	0.396547538	salmon	1.31635402
DUXAP9	ENSG00000225210	1.41887768	0.6993387	2.35E-01	0.395988473	royalblue	1.31584401
SERPINI2	ENSG00000114204	2.88286317	0.5264702	9.07E-02	0.39524222	royalblue	1.31516355
TMEM184B	ENSG00000198792	6.91697035	0.22892563	9.03E-03	0.394990372	royalblue	1.31493399
ERCC6L	ENSG00000186871	3.12821745	0.50154187	0.078085317	0.39453446	salmon	1.31451851
THSD7A	ENSG00000005108	0.98675558	0.76423242	3.21E-01	0.39354081	royalblue	1.31361346
TACC3	ENSG00000013810	9.40486029	0.14585328	0.002385475	0.392293769	salmon	1.31247848
SEMA6C	ENSG00000143434	4.01077342	0.42972672	0.046217582	0.390703697	purple	1.31103272
SLC30A8	ENSG00000164756	2.44420431	0.5728341	1.19E-01	0.390260707	royalblue	1.31063022
CENPL	ENSG00000120334	14.0197749	0.07410552	0.000221416	0.387449386	salmon	1.30807874
RECQL4	ENSG00000160957	8.88831223	0.1596802	0.003133865	0.386731434	salmon	1.30742794
PARPBP	ENSG00000185480	7.57018088	0.20228779	0.006338878	0.38537865	salmon	1.30620257
LYG2	ENSG00000185674	3.87672588	0.4412679	5.00E-02	0.384052472	royalblue	1.30500241
FDPSP8	ENSG00000224763	6.76384856	0.23844091	0.009818797	0.383684889	salmon	1.30466995
TUBA1C	ENSG00000167553	13.2070939	0.07513653	0.00033424	0.38327273	purple	1.30429728
CYB5A	ENSG00000166347	6.92182526	0.22892563	0.009008016	0.380920615	purple	1.30217254
CDCA3	ENSG00000111665	8.47310954	0.17128162	0.003907267	0.380856504	salmon	1.30211467
GAS2L3	ENSG00000139354	3.44360568	0.48007519	0.06459473	0.380609729	salmon	1.30189196
MYB	ENSG00000118513	1.53825124	0.6834251	0.215963048	0.380161133	salmon	1.30148721
AUNIP	ENSG00000127423	6.08178191	0.27525348	0.01428471	0.378371049	salmon	1.29987334
CPS1	ENSG00000021826	1.47093017	0.69266955	2.26E-01	0.37836277	royalblue	1.29986588
C21orf58	ENSG00000160298	7.06957052	0.22336871	0.008312146	0.377644815	salmon	1.29921916

TOB2P1	ENSG00000176933	3.86344018	0.44219437	0.050381503	0.376964767	purple	1.29860689
EIF3FP1	ENSG00000275945	2.15503697	0.60384973	1.43E-01	0.375139065	royalblue	1.29696457
RN7SKP269	ENSG00000202415	1.98076745	0.62187482	0.160468318	0.373925376	purple	1.29587393
WDR76	ENSG00000092470	6.49852528	0.25213718	0.011354059	0.373802438	salmon	1.29576351
LINC02765	ENSG00000234476	2.7107893	0.54629042	0.100843771	0.372412354	purple	1.2945156
ALDH4A1	ENSG00000159423	6.83694893	0.2333461	0.009434661	0.370788988	purple	1.29305979
PPP1R16B	ENSG00000101445	2.18954227	0.59947152	0.140125114	0.368314741	salmon	1.29084407
ZNF367	ENSG00000165244	6.86496285	0.23167073	0.009291588	0.367568128	salmon	1.29017622
SLC19A2	ENSG00000117479	10.2248778	0.12940243	0.001551859	0.367013275	purple	1.28968012
MROH3P	ENSG00000233217	2.34306618	0.58638998	0.127021138	0.366350669	purple	1.28908793
DNAH7	ENSG00000118997	2.26639651	0.59374223	0.133384653	0.365992479	purple	1.28876791
XAGE5	ENSG00000171405	0.87577912	0.78266793	3.50E-01	0.364896267	royalblue	1.28778903
PDZK1P1	ENSG00000215859	8.10724224	0.1845533	0.004750158	0.36480131	purple	1.28770427
LINC02506	ENSG00000251129	0.98392413	0.76471651	3.22E-01	0.36314093	royalblue	1.28622312
ZNF823	ENSG00000197933	3.74255987	0.45294026	0.054095524	0.36272481	salmon	1.28585219
EZH2	ENSG00000106462	8.15170406	0.18360733	0.004638488	0.361038022	salmon	1.28434966
CDC7	ENSG00000097046	6.70149369	0.24059699	0.010159203	0.360957307	salmon	1.2842778
KIAA0895L	ENSG00000196123	7.80098625	0.19347635	5.60E-03	0.360949214	royalblue	1.2842706
ADGRV1	ENSG00000164199	1.36151484	0.70626224	2.44E-01	0.360744409	royalblue	1.2840883
SMC2	ENSG00000136824	9.65065834	0.14072336	0.002096189	0.359687714	salmon	1.28314812
GDPD2	ENSG00000130055	3.35775426	0.48566885	0.067998735	0.359431488	salmon	1.28292025
SOCS5P4	ENSG00000227536	5.26066006	0.33007094	0.022586894	0.358855357	salmon	1.28240802
ORC6	ENSG00000091651	5.98773938	0.27764471	0.015048236	0.358442664	salmon	1.28204123
HMGN5	ENSG00000198157	3.18791281	0.49805506	0.075315405	0.358433282	purple	1.2820329
RDM1	ENSG00000278023	3.67012136	0.46019343	0.0564601	0.357125575	salmon	1.28087135
POC1A	ENSG00000164087	6.96157763	0.22751411	0.008815081	0.35601067	salmon	1.27988188
WDR31	ENSG00000148225	3.15444029	0.50059499	0.07685522	0.35426559	purple	1.27833467
TOR3A	ENSG00000186283	6.39416257	0.25691631	0.012024041	0.354197693	purple	1.27827451

UNC5D	ENSG00000156687	1.51607847	0.68826686	2.19E-01	0.353883773	royalblue	1.2779964
NRM	ENSG00000137404	11.413168	0.10065508	0.000837571	0.353283426	salmon	1.2774647
ARHGEF2	ENSG00000116584	8.87477505	0.1596802	0.003156427	0.352483387	purple	1.27675649
HDGF	ENSG00000143321	12.61533	0.08319566	0.000451861	0.352195074	purple	1.27650136
GSEC	ENSG00000280832	6.31701702	0.26148932	0.012545471	0.350115201	salmon	1.27466241
CD5	ENSG00000110448	1.93230078	0.62663072	1.66E-01	0.35004691	royalblue	1.27460207
APOBEC3B	ENSG00000179750	1.60959016	0.67154417	0.205650265	0.34837617	salmon	1.27312685
UAP1	ENSG00000117143	10.1905612	0.12973798	0.001579915	0.348319038	purple	1.27307643
MIR4258	ENSG00000264349	3.3841631	0.48523216	0.066931549	0.347252269	salmon	1.27213543
C15orf41	ENSG00000186073	6.66624595	0.24238234	0.010356991	0.345156216	purple	1.27028852
KYNU	ENSG00000115919	1.08684919	0.74636827	2.98E-01	0.345002531	royalblue	1.27015321
CALR3	ENSG00000269058	2.46146734	0.5709564	0.117849652	0.344315593	purple	1.26954857
SEPTIN11	ENSG00000138758	4.20940046	0.4102475	0.041172286	0.343936796	salmon	1.26921528
COA6	ENSG00000168275	12.0436364	0.09084778	0.000605501	0.343004146	purple	1.26839504
MIS18A	ENSG00000159055	13.0827221	0.07584465	0.000356064	0.342564253	salmon	1.26800836
RNF183	ENSG00000165188	2.14030984	0.60470443	0.144644678	0.342482574	purple	1.26793657
ARHGAP30	ENSG00000186517	12.2275134	0.08558391	0.000551017	0.341511359	purple	1.26708329
PRC1	ENSG00000198901	5.19358704	0.33639075	0.023457113	0.340686485	salmon	1.26635903
MYBL2	ENSG00000101057	6.29989908	0.26271357	0.012664308	0.33991539	salmon	1.26568236
GBA	ENSG00000177628	7.87163159	0.18999209	0.00538967	0.339779418	purple	1.26556308
LINC00884	ENSG00000233058	4.26280787	0.40614183	0.039917486	0.339406293	purple	1.26523581
C9	ENSG00000113600	2.63441665	0.55360793	1.06E-01	0.338741947	royalblue	1.26465332
COL11A1	ENSG00000060718	0.91692422	0.77726135	3.39E-01	0.338731813	royalblue	1.26464443
CASP17P	ENSG00000235505	0.70735666	0.81335528	0.401072512	0.337393676	midnightblue	1.26347199
OIP5	ENSG00000104147	3.74679768	0.45294026	0.053960501	0.337159928	salmon	1.26326729
ASH1L-IT1	ENSG00000227773	2.21342091	0.59920488	0.137990624	0.336799037	purple	1.26295133
CENPM	ENSG00000100162	4.6555525	0.37546287	0.03184193	0.336403677	salmon	1.26260527
PRCC	ENSG00000143294	16.463968	0.04742106	6.51E-05	0.335984078	purple	1.2622381

MCM4	ENSG00000104738	5.5953493	0.30566205	0.018720527	0.33568346	salmon	1.26197511
MSH2	ENSG00000095002	7.06320822	0.22348169	0.00834094	0.335256884	salmon	1.26160203
ACBD3-AS1	ENSG00000234478	7.89339419	0.18999209	0.005327065	0.33523468	purple	1.26158261
IGSF8	ENSG00000162729	14.240237	0.07215924	0.000198097	0.3335396	purple	1.2601012
TRIM72	ENSG00000177238	0.73848457	0.80809602	3.91E-01	0.333230184	royalblue	1.25983097
HPRT1	ENSG00000165704	10.6789392	0.11799054	0.001225027	0.33232543	salmon	1.25904115
PCNA	ENSG00000132646	7.01979158	0.22565879	0.008540195	0.331758701	salmon	1.25854666
ZNF93	ENSG00000184635	1.96565249	0.62284996	0.162067242	0.329774266	salmon	1.25681671
NUP133	ENSG00000069248	21.7234512	0.01529222	4.97E-06	0.329274065	purple	1.25638103
MLLT11	ENSG00000213190	9.08903543	0.15297416	0.002818028	0.328349727	purple	1.25557632
TSEN15	ENSG00000198860	11.4102183	0.10065508	0.000838847	0.328332352	purple	1.2555612
FRG1-DT	ENSG00000245685	2.88102338	0.52684191	0.090789929	0.327598692	salmon	1.25492287
OR7E12P	ENSG00000189398	0.63764445	0.82493193	4.25E-01	0.327505092	royalblue	1.25484145
RAD51C	ENSG00000108384	12.3146843	0.08425772	0.000526956	0.326582814	salmon	1.25403952
C8orf37	ENSG00000156172	10.0354824	0.1322138	0.001713299	0.32637398	salmon	1.25385801
LINC02804	ENSG00000274372	4.46703495	0.39064831	0.035479074	0.324559887	purple	1.25228236
TCF19	ENSG00000137310	3.48192802	0.47607433	0.06313472	0.324366344	salmon	1.25211437
CKS1B	ENSG00000173207	6.17473228	0.26998775	0.013569417	0.32435281	salmon	1.25210262
TCL6	ENSG00000187621	0.88674527	0.78122635	3.47E-01	0.323143755	royalblue	1.25105373
GLMP	ENSG00000198715	6.42331801	0.25543324	0.011832857	0.323106364	purple	1.25102131
ASF1B	ENSG00000105011	4.48782568	0.38921791	0.035057343	0.320800769	salmon	1.24902363
DARS2	ENSG00000117593	7.70894349	0.19635531	0.005882269	0.320608858	purple	1.24885749
RNF157	ENSG00000141576	1.02912194	0.75693982	3.11E-01	0.320037539	royalblue	1.24836303
C17orf53	ENSG00000125319	3.94582797	0.43548551	0.048006165	0.320012549	salmon	1.24834141
IGSF9	ENSG00000085552	1.17913053	0.73270749	0.278508825	0.319431674	purple	1.24783889
TIMM17A	ENSG00000134375	14.566493	0.06678816	0.000168073	0.319299164	purple	1.24772428
PMF1-BGLAP	ENSG00000260238	9.29478559	0.14761481	0.002527948	0.318445447	purple	1.24698615
ATP8B2	ENSG00000143515	10.0221077	0.1322138	0.001725328	0.316133091	purple	1.24498908

TMEM9	ENSG00000116857	8.97078249	0.15690931	0.002999933	0.31481889	purple	1.2438555
CENPH	ENSG00000153044	6.39402116	0.25691631	0.012024975	0.31331256	salmon	1.24255745
ARHGEF39	ENSG00000137135	9.04470361	0.15371345	0.002884863	0.313208337	salmon	1.24246769
DDR2	ENSG00000162733	4.30175033	0.40267321	3.90E-02	0.312536523	royalblue	1.24188925
LINC02860	ENSG00000222004	1.23778946	0.72294943	2.67E-01	0.312375812	royalblue	1.24175092
CASP12	ENSG00000204403	1.2429822	0.72182368	0.265895849	0.311444434	midnightblue	1.24094952
CSTF2	ENSG00000101811	13.7031583	0.07410552	0.00025987	0.311382816	salmon	1.24089652
LRRCC1	ENSG00000133739	4.9901221	0.35070048	0.026317581	0.31113188	salmon	1.24068071
LINC00404	ENSG00000229520	0.61322566	0.82897942	4.34E-01	0.31111874	royalblue	1.24066941
MIR646HG	ENSG00000228340	0.74911679	0.80513257	3.88E-01	0.310679932	royalblue	1.2402921
SMG7-AS1	ENSG00000232860	6.53006238	0.25054908	0.011159281	0.310299737	purple	1.23996529
LINC01993	ENSG00000204277	1.55337268	0.68089348	0.213726541	0.30868724	purple	1.23858016
BRI3BP	ENSG00000184992	4.27610102	0.40441516	0.039611485	0.306609308	salmon	1.2367975
KIF22	ENSG00000079616	9.02773648	0.15417003	0.002910869	0.306265384	salmon	1.2365027
CACYBPP1	ENSG00000225928	2.48400596	0.56839636	0.116188657	0.304953708	purple	1.235379
IDH1-AS1	ENSG00000231908	3.05607796	0.50923461	0.081580705	0.304939202	purple	1.23536658
PRIM2	ENSG00000146143	13.2382253	0.07513653	0.000328993	0.304526393	salmon	1.23501314
CENPK	ENSG00000123219	3.6844809	0.45810933	0.055982751	0.30431999	salmon	1.23483646
SH3BP5L	ENSG00000175137	11.3120312	0.10132658	0.000882469	0.303597488	purple	1.23421821
MEF2D	ENSG00000116604	13.5029251	0.07513653	0.000287624	0.302456953	purple	1.23324288
REXO5	ENSG00000005189	4.07290916	0.42486803	0.044572403	0.302064614	salmon	1.23290754
TRIM11	ENSG00000154370	14.4909872	0.06761889	0.000174583	0.300896844	purple	1.23190999
EME1	ENSG00000154920	5.12776847	0.34092072	0.024345113	0.300892288	salmon	1.2319061
FEN1	ENSG00000168496	7.15691855	0.21979852	0.007927021	0.300486474	salmon	1.23155962
PIMREG	ENSG00000129195	1.23008311	0.72475051	0.268385126	0.29958791	salmon	1.2307928
SLC9C2	ENSG00000162753	0.81374149	0.79414243	3.68E-01	0.299078763	royalblue	1.23035851
LCA5L	ENSG00000157578	3.38378053	0.48523216	0.066946879	0.298422516	purple	1.22979898
OAZ3	ENSG00000143450	8.68195833	0.16308575	0.003496446	0.296438696	purple	1.22810907

CDC25A	ENSG00000164045	4.35948879	0.39745335	0.037747547	0.296191711	salmon	1.22789884
RABIF	ENSG00000183155	11.2122512	0.10214637	0.000929167	0.295508282	purple	1.2273173
TUBB	ENSG00000196230	7.31586902	0.21310928	0.007272618	0.295337681	salmon	1.22717218
NSL1	ENSG00000117697	9.21326441	0.14946792	0.002639046	0.294960769	purple	1.22685162
CENPO	ENSG00000138092	8.11573331	0.18450799	0.004728621	0.29452612	salmon	1.22648205
LINC01775	ENSG00000267201	1.23400586	0.72391191	0.267625036	0.293171697	salmon	1.22533115
ANP32E	ENSG00000143401	10.2861405	0.12852	0.001503029	0.292768681	purple	1.2249889
LBR	ENSG00000143815	9.6732672	0.14072336	0.002071448	0.291621881	purple	1.22401555
SMG8	ENSG00000167447	7.50122599	0.20551465	0.006579171	0.291411265	purple	1.22383687
RPL26P29	ENSG00000234806	2.04546886	0.61398122	0.153824751	0.291246961	salmon	1.2236975
MAP3K12	ENSG00000139625	4.55698648	0.38238405	0.033691986	0.290864092	salmon	1.22337279
RRP15	ENSG00000067533	11.8643737	0.09386565	0.000663896	0.289868961	purple	1.22252923
CHAF1B	ENSG00000159259	3.04835183	0.50943837	0.081965001	0.289363504	salmon	1.22210099
FAM234B	ENSG00000084444	2.59997389	0.55701459	0.108042407	0.289222079	purple	1.22198119
KLHL23	ENSG00000213160	1.96879459	0.62284996	0.16173336	0.288794628	salmon	1.22161919
TBC1D31	ENSG00000156787	7.16753222	0.21903534	0.007881492	0.288107736	salmon	1.22103769
RUSC1	ENSG00000160753	7.55200634	0.20360468	0.006401324	0.287864137	purple	1.22083154
ASTN2	ENSG00000148219	6.15848947	0.2704572	0.013691686	0.287371159	purple	1.22041444
TPGS2	ENSG00000134779	8.68210494	0.16308575	0.003496173	0.287249771	salmon	1.22031176
GINS2	ENSG00000131153	2.83972488	0.53206668	0.093123964	0.286628112	salmon	1.21978604
TUBG1	ENSG00000131462	10.36949	0.12808308	0.001439099	0.28622491	purple	1.21944519
RNPEP	ENSG00000176393	11.5132863	0.09811222	0.000795413	0.284474213	purple	1.2179663
HEATR1	ENSG00000119285	10.9268259	0.10877251	0.001077132	0.284383207	purple	1.21788947
ASCL5	ENSG00000232237	3.3416121	0.48601849	0.068660042	0.283685527	purple	1.21730065
TAF1A-AS1	ENSG00000225265	4.47470602	0.39032453	0.035322852	0.283150978	purple	1.2168497
LINC01145	ENSG00000272419	3.39784248	0.48418809	0.066385852	0.283088672	midnightblue	1.21679714
NVL	ENSG00000143748	16.6471965	0.04546232	5.94E-05	0.282392166	purple	1.21620984
TARS2	ENSG00000143374	12.2835518	0.08488556	0.000535424	0.282308792	purple	1.21613956

LGALS8-AS1	ENSG00000223776	3.09491818	0.50476041	0.079678175	0.282272245	purple	1.21610875
KIAA1841	ENSG00000162929	3.90457491	0.43885069	0.049180307	0.281872702	salmon	1.215772
NCAPD2	ENSG00000010292	7.48287309	0.20656223	0.00664469	0.281645253	salmon	1.21558035
PSMD4	ENSG00000159352	12.9446222	0.07725874	0.000381999	0.280604299	purple	1.21470358
WNT6	ENSG00000115596	1.6807594	0.66142473	1.96E-01	0.279883234	royalblue	1.21409662
MTMR11	ENSG00000014914	1.59306776	0.67357589	0.207985653	0.279681774	purple	1.21392709
MCM3	ENSG00000112118	5.45165429	0.31751671	0.020288442	0.279466579	salmon	1.21374603
ASAH2B	ENSG00000204147	2.7702027	0.54155279	0.09720269	0.279440584	purple	1.21372416
B4GALT3	ENSG00000158850	10.5455616	0.12211351	0.001313006	0.279391501	purple	1.21368287
LINC01703	ENSG00000225518	1.56527082	0.67905859	0.211986188	0.27865576	purple	1.21306408
RNF157-AS1	ENSG00000267128	1.08234996	0.74729622	2.99E-01	0.277776542	royalblue	1.21232503
FH	ENSG00000091483	9.6624731	0.14072336	0.002083223	0.277717414	purple	1.21227534
GPATCH2	ENSG00000092978	11.105554	0.10359968	0.000981891	0.277546445	purple	1.21213169
MCM6	ENSG00000076003	6.11317825	0.27360525	0.014038834	0.277195558	salmon	1.21183691
CEP85	ENSG00000130695	9.04516879	0.15371345	0.002884153	0.27518162	salmon	1.21014642
OTUD7B	ENSG00000264522	8.23886603	0.1796277	0.004427317	0.275041295	purple	1.21002872
NR1I3	ENSG00000143257	3.58744324	0.46580028	0.059294467	0.275013809	purple	1.21000567
VAMP7	ENSG00000124333	7.93016074	0.18856183	0.005222986	0.274672036	salmon	1.20971906
RBM8B	ENSG00000258427	4.87630361	0.35837624	0.02807464	0.27341621	purple	1.20866649
INCENP	ENSG00000149503	8.00012669	0.18657923	0.005030651	0.272652256	salmon	1.20802663
KLHDC9	ENSG00000162755	2.45675537	0.57171839	0.118200226	0.272359637	purple	1.20778163
TRAIP	ENSG00000183763	4.35166219	0.39856751	0.037918492	0.271442191	salmon	1.20701382
SUV39H1	ENSG00000101945	10.3739212	0.12808308	0.001435778	0.271322059	salmon	1.20691331
FAM126A	ENSG00000122591	2.40463472	0.57847251	0.122156424	0.270071231	purple	1.20586736
PKIA-AS1	ENSG00000254266	1.32721777	0.7111902	0.250327421	0.269658201	purple	1.20552219
DESI2	ENSG00000121644	10.7639421	0.1153194	0.001172114	0.269041133	purple	1.20500667
TPM3	ENSG00000143549	9.94409803	0.1337992	0.001797224	0.267454019	purple	1.20368177
XRCC2	ENSG00000196584	2.19931298	0.59947152	0.139247251	0.26711503	salmon	1.20339897

H3F3BP1	ENSG00000236534	2.98768986	0.51686326	0.085051523	0.26711445	purple	1.20339849
BRCA1	ENSG00000012048	3.35418282	0.48566885	0.068144456	0.26646064	salmon	1.20285325
LINC00997	ENSG00000281332	2.02039077	0.6176497	0.156361782	0.266237023	salmon	1.20266682
TUBBP1	ENSG00000127589	3.35348952	0.48566885	0.068172783	0.26617678	salmon	1.2026166
KREMEN2	ENSG00000131650	1.05931098	0.75251643	0.304300368	0.265963124	salmon	1.20243851
C1orf112	ENSG00000000460	3.98295488	0.43186174	0.046974887	0.265927934	salmon	1.20240918
DHFR	ENSG00000228716	5.23064299	0.33249782	0.022972102	0.265880431	salmon	1.20236959
SLC9A6	ENSG00000198689	9.68409735	0.14072336	0.002059702	0.265421365	salmon	1.20198706
ATAD5	ENSG00000176208	4.78378323	0.36560736	0.029593283	0.264651286	salmon	1.20134564
RASAL2-AS1	ENSG00000224687	1.35817267	0.7069414	0.244890616	0.26446415	purple	1.20118982
CIP2A	ENSG00000163507	3.08685989	0.50569489	0.080068908	0.263775985	salmon	1.20061699
AIFM2	ENSG00000042286	6.29460045	0.2628409	0.012701329	0.263608644	salmon	1.20047773
BCAN	ENSG00000132692	1.10469854	0.74499368	0.294184076	0.263461007	purple	1.20035489
SH2D6	ENSG00000152292	2.25107949	0.59550655	0.134698373	0.262810018	purple	1.19981337
H2AFX	ENSG00000188486	5.72536571	0.29497387	0.01741038	0.261980885	salmon	1.19912403
SLC44A5	ENSG00000137968	0.33675355	0.88384753	5.62E-01	0.261114892	royalblue	1.19840446
TUBBP2	ENSG00000214222	1.68773299	0.66088362	0.19501661	0.26107458	salmon	1.19837097
TMPO	ENSG00000120802	6.63700597	0.24413635	0.010524081	0.260127362	salmon	1.19758442
TFB2M	ENSG00000162851	12.1713432	0.08719824	0.000567111	0.259725716	purple	1.19725106
CCDC14	ENSG00000175455	3.75767245	0.4525605	0.053615655	0.258594488	salmon	1.19631266
TONSL	ENSG00000160949	6.70567394	0.24059699	0.010136006	0.258028128	salmon	1.19584311
CENPU	ENSG00000151725	3.55489507	0.46910992	0.060451677	0.25550689	salmon	1.19375509
CLCN5	ENSG00000171365	1.83658611	0.6415034	0.176492713	0.255272139	purple	1.19356087
DCAF8L1	ENSG00000226372	0.43521245	0.86307896	5.10E-01	0.255185401	royalblue	1.19348911
EDEM3	ENSG00000116406	3.99606313	0.43152734	0.046616414	0.255178397	purple	1.19348332
SNAP47-AS1	ENSG00000230005	2.00703258	0.61978983	0.157732642	0.255122315	purple	1.19343692
C1orf131	ENSG00000143633	9.01369706	0.15497541	0.00293257	0.254566604	purple	1.19297731
MMS22L	ENSG00000146263	4.97713827	0.3515826	0.026512068	0.254060872	salmon	1.19255919

DNMT3B	ENSG00000088305	1.70258398	0.65901238	0.193069685	0.253818794	salmon	1.1923591
VBP1	ENSG00000155959	8.90647332	0.1593486	0.003103855	0.253493345	salmon	1.19209015
CREB3L4	ENSG00000143578	5.14644942	0.33967398	0.024089566	0.251456443	purple	1.19040826
CNPY4	ENSG00000166997	5.72318992	0.29500505	0.017431503	0.250973068	purple	1.19000948
RNFT2	ENSG00000135119	1.42311829	0.69892805	0.23394589	0.250501239	salmon	1.18962036
ATP1A1	ENSG00000163399	7.27427528	0.21370613	0.007438298	0.249439684	purple	1.18874534
RBBP8	ENSG00000101773	6.07889189	0.27525348	0.014307566	0.249300323	salmon	1.18863051
DIXDC1	ENSG00000150764	0.67293827	0.81893216	4.13E-01	0.24921751	royalblue	1.18856229
INTS7	ENSG00000143493	6.72014853	0.24038158	0.010056106	0.249101293	salmon	1.18846655
UHMK1	ENSG00000152332	6.52181452	0.25109815	0.011209885	0.247846296	purple	1.18743315
FRG1JP	ENSG00000215548	0.43592046	0.86307896	5.10E-01	0.247839936	royalblue	1.18742792
ASRGL1	ENSG00000162174	3.39610869	0.48418809	0.066454751	0.247640935	salmon	1.18726414
RRM1	ENSG00000167325	6.73687973	0.23969773	0.009964557	0.247226216	salmon	1.18692289
STK26	ENSG00000134602	6.18590416	0.26998775	0.013485976	0.246916234	salmon	1.1866679
DUSP12	ENSG00000081721	9.63101503	0.14118615	0.002117931	0.246908261	purple	1.18666134
LINC01694	ENSG00000233922	0.37107325	0.87632174	5.43E-01	0.246622908	royalblue	1.18642665
CHAF1A	ENSG00000167670	5.05176811	0.34701221	0.025414315	0.246247906	salmon	1.1861183
DBF4B	ENSG00000161692	4.72072872	0.37199524	0.030677422	0.246222451	salmon	1.18609737
DAP3	ENSG00000132676	13.1800153	0.07513653	0.000338873	0.246028395	purple	1.18593784
CDCA4	ENSG00000170779	5.80086038	0.29033671	0.016693691	0.245345616	salmon	1.18537671
TDRKH	ENSG00000182134	6.90430668	0.22931255	0.009094418	0.244644019	purple	1.18480039
GLRX2	ENSG00000023572	7.16855596	0.21903534	0.007877115	0.244642137	purple	1.18479884
ARPC5	ENSG00000162704	5.77579754	0.29197736	0.01692816	0.243404433	purple	1.18378283
TSACC	ENSG00000163467	2.45369771	0.57190159	0.118428339	0.243150846	purple	1.18357477
ZNF887P	ENSG00000267500	1.60125144	0.67273721	0.206825005	0.242331436	salmon	1.18290272
AIFM1	ENSG00000156709	7.56743048	0.20228779	0.006348288	0.242288042	purple	1.18286714
ZWILCH	ENSG00000174442	5.8665733	0.28521351	0.016094798	0.242050337	salmon	1.18267226
PAPPA	ENSG00000182752	2.20007365	0.59947152	1.39E-01	0.241538716	royalblue	1.18225293

EXOC8	ENSG00000116903	5.83907318	0.28804896	0.016342669	0.241378648	purple	1.18212177
PIGM	ENSG00000143315	3.69950003	0.45775796	0.05548808	0.241121269	purple	1.18191089
TMEM79	ENSG00000163472	4.91579352	0.35501323	0.02745151	0.240634361	purple	1.18151207
IPO9	ENSG00000198700	13.2803653	0.07513653	0.000322024	0.240184793	salmon	1.18114394
DCK	ENSG00000156136	2.79467557	0.53913256	0.095745031	0.239998217	salmon	1.1809912
LINC02362	ENSG00000249096	1.15925	0.73544867	0.282589384	0.23971819	midnightblue	1.18076199
ATPSCKMT	ENSG00000150756	6.17218627	0.26998775	0.013588507	0.238927451	purple	1.180115
CENPN	ENSG00000166451	5.28564728	0.32888969	0.022271372	0.238511924	salmon	1.17977515
TUBAP2	ENSG00000214391	3.15908334	0.50059499	0.07663961	0.238311631	salmon	1.17961137
PDZRN4	ENSG00000165966	0.80231951	0.7959438	0.37120459	0.237237456	midnightblue	1.1787334
CHD1L	ENSG00000131778	7.23484909	0.21629025	0.007598929	0.236952592	purple	1.17850068
PDXP	ENSG00000241360	4.30296942	0.40267321	0.039000524	0.236524164	salmon	1.17815076
RBFADN	ENSG00000261126	1.41284603	0.69993316	0.235636691	0.23582411	purple	1.17757921
MCM5	ENSG00000100297	4.5968913	0.37983196	0.032929735	0.235753447	salmon	1.17752154
RIBC2	ENSG00000128408	0.62070491	0.82732658	0.431481717	0.235271953	salmon	1.17712861
TUSC3	ENSG00000104723	0.3729209	0.87631257	5.42E-01	0.235078771	royalblue	1.176971
ECM1	ENSG00000143369	4.04050757	0.42777322	0.045422419	0.234592528	purple	1.17657438
PIAS3	ENSG00000131788	12.4056058	0.08323217	0.000502996	0.233410905	purple	1.17561112
ENTPD1	ENSG00000138185	1.833513	0.64205977	0.176853839	0.232581527	salmon	1.17493547
POU5F1	ENSG00000204531	1.53728373	0.68373952	0.216107094	0.232353416	salmon	1.17474971
PSMC3IP	ENSG00000131470	4.56805492	0.38095272	0.033478719	0.232201738	salmon	1.17462621
ATF6	ENSG00000118217	4.53657664	0.38479111	0.034088998	0.231668838	purple	1.17419241
FBXO28	ENSG00000143756	10.0875084	0.13216723	0.001667316	0.231044602	purple	1.17368446
PIPSL	ENSG00000180764	4.92459121	0.35440889	0.027314669	0.23028475	purple	1.17306646
PDZK1	ENSG00000174827	5.05443026	0.34694203	0.025376046	0.230175756	purple	1.17297784
GAS6-DT	ENSG00000272695	0.90552184	0.77903844	0.342162809	0.230146646	purple	1.17295417
FIGNL1	ENSG00000132436	3.35032767	0.48566885	0.06830213	0.228748258	salmon	1.17181779
H3F3A	ENSG00000163041	8.75218786	0.16256807	0.00336846	0.228700221	purple	1.17177877

DIPK2A	ENSG00000181744	3.24245226	0.49377824	0.072877395	0.228671037	purple	1.17175507
MSTO2P	ENSG00000203761	3.29085028	0.49179922	0.070785142	0.2284265	purple	1.17155647
PMF1	ENSG00000160783	9.34084876	0.14598224	0.002467298	0.227958965	purple	1.17117687
PSMB4	ENSG00000159377	11.0973235	0.10368439	0.000986083	0.227675448	purple	1.17094673
DONSON	ENSG00000159147	8.12587239	0.18435965	0.004703035	0.227454467	salmon	1.17076739
ABCB6	ENSG00000115657	5.37010666	0.32363264	0.021238458	0.227413607	salmon	1.17073423
ACBD3	ENSG00000182827	8.00734167	0.18653743	0.005011235	0.227263632	purple	1.17061254
LAMTOR2	ENSG00000116586	6.86823728	0.23167073	0.009275012	0.226707867	purple	1.17016167
TMEM164	ENSG00000157600	3.88594345	0.44072039	0.049720559	0.225394209	salmon	1.16909665
NCSTN	ENSG00000162736	10.0386683	0.1322138	0.001710446	0.225351357	purple	1.16906193
PARP1	ENSG00000143799	7.50670557	0.20551465	0.006559738	0.225310209	purple	1.16902859
GET1	ENSG00000182093	5.82288692	0.28915181	0.016490411	0.225236653	purple	1.16896899
MRPL9	ENSG00000143436	9.89531832	0.13477394	0.001843728	0.22470144	purple	1.1685354
CEP41	ENSG00000106477	2.22914377	0.59729492	1.37E-01	0.223998844	royalblue	1.16796646
TPI1P2	ENSG00000230359	2.62229439	0.55407189	0.106548108	0.223958873	purple	1.1679341
HTATSF1	ENSG00000102241	7.76614358	0.1951003	0.005704011	0.223813741	salmon	1.16781661
RPS2P32	ENSG00000232818	2.25375418	0.59508961	0.134467925	0.223578082	purple	1.16762587
HMGXB4	ENSG00000100281	9.85750011	0.13534279	0.001880625	0.223378027	salmon	1.16746397
SNRPE	ENSG00000182004	7.05088994	0.2237177	0.008396982	0.222986932	purple	1.16714753
PRDX1P1	ENSG00000214121	0.71563688	0.81191	3.98E-01	0.222857581	royalblue	1.16704289
UBL4A	ENSG00000102178	6.9388763	0.22870936	0.008924733	0.22166953	salmon	1.16608223
JMJD4	ENSG00000081692	8.32403593	0.1769125	0.004230483	0.221220916	purple	1.16571969
LINC00680	ENSG00000215190	3.65640971	0.4610232	0.056919969	0.220904539	purple	1.16546408
E2F7	ENSG00000165891	0.97969897	0.76565465	0.323165927	0.220013128	salmon	1.16474419
UBAP2L	ENSG00000143569	11.7665196	0.09517333	0.000698156	0.219855892	purple	1.16461725
PYCR2	ENSG00000143811	5.66340915	0.29953785	0.018022393	0.219654221	purple	1.16445446
BRCC3	ENSG00000185515	5.7461098	0.29431968	0.017210325	0.219589561	salmon	1.16440227
TSFM	ENSG00000123297	7.66878449	0.19691583	0.00601083	0.219404406	purple	1.16425284

AGMAT	ENSG00000116771	0.64666221	0.8229013	0.42202129	0.218562832	purple	1.16357389
CDC6	ENSG00000094804	2.03999962	0.61472362	0.15437402	0.217639128	salmon	1.16282914
FAM122B	ENSG00000156504	6.30973867	0.26218235	0.012595858	0.216651861	salmon	1.16203366
ADSS	ENSG00000035687	9.05001665	0.15371345	0.002876768	0.216366967	purple	1.16180421
MIR548AA1	ENSG00000207704	0.49179997	0.85109559	0.483733958	0.216318687	salmon	1.16176533
POLD3	ENSG00000077514	4.65443793	0.37546287	0.031862243	0.215365838	salmon	1.16099828
CD55	ENSG00000196352	3.282754	0.4919307	0.071130586	0.21522085	purple	1.16088161
SPRTN	ENSG00000010072	8.86865027	0.1596802	0.003166689	0.215085166	purple	1.16077244
PIP5K1A	ENSG00000143398	8.32164496	0.1769125	0.004235884	0.214753288	purple	1.16050544
KLLN	ENSG00000227268	2.86244669	0.52877306	0.09183181	0.214621183	purple	1.16039918
TUBA1B	ENSG00000123416	4.79967057	0.36417714	0.029326507	0.213950886	salmon	1.15986017
DPM3	ENSG00000179085	2.34001325	0.5870864	0.127267929	0.21384478	purple	1.15977487
SDHC	ENSG00000143252	7.36179814	0.2113578	0.007094074	0.213831784	purple	1.15976442
RBM8A	ENSG00000265241	12.5828017	0.08323217	0.000459432	0.213510965	purple	1.15950655
TAF5L	ENSG00000135801	8.31639959	0.17707483	0.004247759	0.213462041	purple	1.15946723
PEX19	ENSG00000162735	7.71829585	0.19614682	0.005852737	0.212642448	purple	1.15880872
BCAP31	ENSG00000185825	5.95644359	0.27957208	0.015311603	0.212324485	purple	1.15855335
POLE2	ENSG00000100479	1.4735301	0.69232708	0.225858191	0.211961558	salmon	1.15826194
MSTO1	ENSG00000125459	3.69697367	0.45795206	0.055570961	0.211663049	purple	1.15802231
C2orf27A	ENSG00000197927	1.08337533	0.74724375	0.298881765	0.211217098	purple	1.15766441
RBBP9	ENSG00000089050	2.49193137	0.56691558	0.11561078	0.211008369	purple	1.15749693
PRKAB2	ENSG00000131791	5.40737197	0.32189796	0.020798703	0.210799069	purple	1.15732902
TOMM40L	ENSG00000158882	7.31339057	0.21310928	0.007282384	0.210628881	purple	1.1571925
FAM50A	ENSG00000071859	6.5698494	0.24870256	0.010918461	0.210500808	purple	1.15708978
ILF2	ENSG00000143621	8.87442371	0.1596802	0.003157015	0.210200864	purple	1.15684924
SF3B4	ENSG00000143368	6.3490091	0.25921982	0.012326454	0.210132826	purple	1.15679468
STIM1	ENSG00000167323	7.04407788	0.2238314	0.008428139	0.209877213	salmon	1.15658974
AGTRAP	ENSG00000177674	3.06491022	0.50804941	0.081143782	0.209836166	purple	1.15655684

NXT2	ENSG00000101888	3.60535082	0.46514444	0.058667908	0.209815383	salmon	1.15654018
CHTF18	ENSG00000127586	3.16608904	0.50059499	0.076315523	0.209428553	salmon	1.15623011
LMNB2	ENSG00000176619	5.55205449	0.3102474	0.019179208	0.20931544	salmon	1.15613946
ZNF571-AS1	ENSG00000267470	0.47035674	0.85538476	0.493415942	0.209144608	purple	1.15600257
CEP72	ENSG00000112877	4.5274781	0.38536894	0.034267565	0.209123329	salmon	1.15598552
SRP9	ENSG00000143742	9.38756254	0.14585328	0.002407309	0.208560366	purple	1.15553453
CDYL	ENSG00000153046	5.38032755	0.32312977	0.02111689	0.208110941	salmon	1.15517461
H3F3AP4	ENSG00000235655	2.44419716	0.5728341	0.119140245	0.20806442	purple	1.15513736
TMCO1	ENSG00000143183	4.69310344	0.37284517	0.031165452	0.207999951	purple	1.15508575
DTYMK	ENSG00000168393	6.82082306	0.23392227	0.009518048	0.207849142	salmon	1.15496501
USP31	ENSG00000103404	4.57018608	0.38080385	0.033437819	0.207427627	purple	1.15462761
SLX4	ENSG00000188827	4.82066735	0.36249493	0.028977796	0.207360211	salmon	1.15457366
DENND4B	ENSG00000198837	9.7608953	0.13883748	0.001978337	0.206963313	purple	1.15425607
DEDD	ENSG00000158796	10.4148797	0.12779565	0.001405457	0.206949226	purple	1.1542448
LINC01206	ENSG00000242512	0.57748603	0.83437096	0.447967375	0.206372325	midnightblue	1.15378333
G6PD	ENSG00000160211	4.72707066	0.37187849	0.030566521	0.206371864	salmon	1.15378296
HAUS1	ENSG00000152240	5.67203212	0.29880996	0.017935893	0.205651982	salmon	1.15320739
GMNN	ENSG00000112312	3.74108633	0.45298604	0.054142557	0.204498663	salmon	1.15228586
SLF1	ENSG00000133302	6.0956137	0.27435323	0.014175843	0.204251318	salmon	1.15208832
TBX4	ENSG00000121075	1.37642393	0.70415559	2.42E-01	0.204132381	royalblue	1.15199334
CDK2	ENSG00000123374	5.81966014	0.28915181	0.01652003	0.203728323	salmon	1.15167075
XK	ENSG00000047597	0.62487083	0.82649764	0.429941922	0.201935686	salmon	1.15024062
MRPS23	ENSG00000181610	8.2341816	0.1796277	0.004438411	0.201674608	purple	1.15003248
GNPAT	ENSG00000116906	10.6908621	0.11799054	0.001217461	0.201193215	purple	1.14964881
RAB4A	ENSG00000168118	1.74521628	0.6545738	0.187606069	0.201146293	purple	1.14961142
TTC25	ENSG00000204815	0.91633658	0.77748749	0.339301432	0.200878672	purple	1.14939818
UBE2CP2	ENSG00000265939	0.94161004	0.77188802	0.332739062	0.200690172	salmon	1.14924801
SOAT1	ENSG00000057252	5.1395149	0.34006666	0.024184099	0.200401826	purple	1.14901834

LRRC20	ENSG00000172731	2.36697457	0.5835479	0.125106829	0.200202867	salmon	1.14885989
ADAM15	ENSG00000143537	2.67572421	0.55057298	0.103062823	0.199694227	purple	1.14845492
VMA21	ENSG00000160131	5.33951221	0.32478778	0.02160673	0.198999047	salmon	1.14790166
BCL9	ENSG00000116128	3.26311808	0.49271972	0.071975964	0.198219324	salmon	1.14728143
ARHGAP19	ENSG00000213390	5.92020582	0.28131258	0.01562254	0.197969016	salmon	1.14708239
PRRC2C	ENSG00000117523	8.86803653	0.1596802	0.003167719	0.197619005	purple	1.14680413
RTKN2	ENSG00000182010	0.71443757	0.81199714	0.398728539	0.197298345	salmon	1.14654926
TMEM106C	ENSG00000134291	3.65672192	0.4610232	0.056909453	0.196957967	salmon	1.14627879
RAB3GAP2	ENSG00000118873	8.30767388	0.17741802	0.004267588	0.19685284	purple	1.14619526
SNAP47	ENSG00000143740	5.03812581	0.34798265	0.025611378	0.19676071	purple	1.14612207
RRNAD1	ENSG00000143303	6.46395511	0.25319108	0.01157161	0.196455323	purple	1.14587949
PHTF2	ENSG00000006576	4.23221307	0.40851856	0.040631284	0.196044035	salmon	1.14555286
MRPL24	ENSG00000143314	4.69447713	0.37284517	0.031140992	0.195808964	purple	1.14536622
LIX1L	ENSG00000271601	3.41361115	0.48265966	0.065762734	0.195358203	purple	1.14500842
OPN3	ENSG00000054277	1.71615963	0.6574677	0.191309887	0.194980915	purple	1.14470902
GSPT1	ENSG00000103342	7.88090329	0.18999209	0.005362906	0.194919235	purple	1.14466008
RFC4	ENSG00000163918	5.4348255	0.31891689	0.020480806	0.194886107	salmon	1.1446338
PGRMC1	ENSG00000101856	4.7005634	0.37251614	0.031032866	0.194711624	salmon	1.14449537
SCNM1	ENSG00000163156	7.37524716	0.21085225	0.00704265	0.194310661	purple	1.14417733
GPR162	ENSG00000250510	0.26084958	0.8984212	6.10E-01	0.193801682	royalblue	1.14377374
APH1A	ENSG00000117362	6.22061287	0.26821422	0.013230115	0.193678127	purple	1.14367579
FAAP24	ENSG00000131944	2.9258989	0.5228469	0.088325892	0.19362797	salmon	1.14363602
BEND3	ENSG00000178409	2.77309454	0.54097691	0.097029185	0.193543213	purple	1.14356884
P3H1	ENSG00000117385	6.83202643	0.2333461	0.009460035	0.193185264	purple	1.14328514
NUTM2D	ENSG00000214562	3.05095105	0.50943837	0.081835498	0.192736624	purple	1.14292967
CLTC	ENSG00000141367	7.58674355	0.20192482	0.006282516	0.192659693	purple	1.14286872
SMIM22	ENSG00000267795	0.29944631	0.88956126	5.85E-01	0.192155093	royalblue	1.14246906
GORAB-AS1	ENSG00000231407	2.1238249	0.6065444	0.146194685	0.19187436	purple	1.14224677

SLFN13	ENSG00000154760	3.42990062	0.48097441	0.065125626	0.191822716	salmon	1.14220588
TIPRL	ENSG00000143155	7.0061694	0.22577747	0.008603717	0.191719072	purple	1.14212383
MRPL55	ENSG00000162910	5.76226002	0.29308899	0.017056226	0.190771941	purple	1.14137427
CD99L2	ENSG00000102181	3.6007893	0.46529741	0.058826834	0.190614411	salmon	1.14124965
PFN1P6	ENSG00000227212	1.18498298	0.73194667	0.277321821	0.190607673	purple	1.14124431
TMEM183A	ENSG00000163444	8.64075017	0.16395449	0.003573849	0.190489272	purple	1.14115066
CHEK1	ENSG00000149554	3.26751194	0.49256303	0.071785861	0.189995334	salmon	1.14076003
UBQLN4P1	ENSG00000241627	2.17905885	0.59980399	0.141073965	0.189384376	purple	1.14027704
TADA1	ENSG00000152382	7.85259692	0.19043982	0.005445048	0.189223182	purple	1.14014964
KLHL12	ENSG00000117153	7.14409403	0.22029895	0.007982394	0.189218013	purple	1.14014555
NFATC2IP	ENSG00000176953	11.7292224	0.09517333	0.000711684	0.188468448	salmon	1.13955333
NBPF9	ENSG00000269713	8.35635655	0.17573372	0.004158155	0.187944887	purple	1.13913986
NME7	ENSG00000143156	3.09592719	0.50476041	0.079629396	0.187783356	purple	1.13901232
C8orf33	ENSG00000182307	9.64063293	0.14091142	0.002107257	0.187157264	purple	1.13851813
ARHGEF11	ENSG00000132694	2.90496803	0.52506125	0.089465973	0.186711626	purple	1.1381665
COPA	ENSG00000122218	6.7371012	0.23969773	0.009963351	0.186242008	purple	1.13779607
AMDHD1	ENSG00000139344	1.56435317	0.67905859	0.212119811	0.185953619	purple	1.13756866
C1orf56	ENSG00000143443	2.16513862	0.60233382	0.142345115	0.185928684	purple	1.13754899
PRIM1	ENSG00000198056	2.70606026	0.54684345	0.101139954	0.185748224	salmon	1.13740671
TRIM37	ENSG00000108395	8.66196908	0.16386161	0.003533776	0.185556782	salmon	1.13725579
PRKDC	ENSG00000253729	5.1546412	0.33912368	0.023978391	0.185101651	salmon	1.13689708
NDUFS2	ENSG00000158864	6.14779581	0.27118422	0.013772805	0.185008214	purple	1.13682345
SSX2IP	ENSG00000117155	2.36007724	0.58449931	1.26E-01	0.184463861	royalblue	1.13639458
FLAD1	ENSG00000160688	6.06473457	0.27525348	0.014420078	0.184207737	purple	1.13619286
TIMELESS	ENSG00000111602	2.21122163	0.59928233	0.138185675	0.184123502	salmon	1.13612652
CBX8	ENSG00000141570	2.66869421	0.55111332	1.04E-01	0.183902537	royalblue	1.13595252
MTX1	ENSG00000173171	5.17452755	0.33776704	0.023710724	0.183896131	purple	1.13594748
IARS2	ENSG00000067704	4.63167627	0.37695764	0.032280062	0.183746953	purple	1.13583002

LGALS8	ENSG00000116977	2.60436095	0.55624501	0.107746888	0.183583033	purple	1.13570098
FANCB	ENSG00000181544	2.22001814	0.59857634	0.137407382	0.182995065	salmon	1.13523822
SLC25A44	ENSG00000160785	8.14518138	0.18401102	0.004654701	0.182659968	purple	1.13497457
FATE1	ENSG00000147378	0.16016767	0.92587031	6.89E-01	0.182629971	royalblue	1.13495097
LAMC1	ENSG00000135862	2.17876827	0.59980399	0.141100369	0.181082976	purple	1.13373462
ELK4	ENSG00000158711	4.59766674	0.37983196	0.032915103	0.180677537	purple	1.13341605
CNDP2	ENSG00000133313	2.88045576	0.52684191	0.090821572	0.180472254	purple	1.13325479
PLAGL2	ENSG00000126003	2.92810813	0.52254444	0.088206485	0.180314916	purple	1.1331312
CLK2	ENSG00000176444	5.4706575	0.31643915	0.020073482	0.180186761	purple	1.13303055
POLA1	ENSG00000101868	3.73107768	0.45392724	0.054463177	0.180040776	salmon	1.13291591
LINC01136	ENSG00000233791	0.67332481	0.8188886	0.412624076	0.180027921	purple	1.13290581
FDXR	ENSG00000161513	2.46566288	0.57068267	0.117538472	0.179901192	salmon	1.1328063
CEP170	ENSG00000143702	5.25150508	0.33048839	0.02270366	0.179618576	salmon	1.13258441
ACP6	ENSG00000162836	2.6247335	0.55407189	0.106386203	0.179611639	purple	1.13257896
SMG7	ENSG00000116698	9.9473425	0.1337992	0.001794174	0.179541809	purple	1.13252415
PSMD1	ENSG00000173692	8.51735675	0.16917337	0.003816285	0.179390728	purple	1.13240555
TPTEP2	ENSG00000244627	0.40122898	0.8699771	0.52699542	0.179056743	midnightblue	1.13214343
HCFC1	ENSG00000172534	6.53825209	0.25007055	0.011109266	0.178788468	salmon	1.13193292
POLD1	ENSG00000062822	3.80933306	0.44651465	0.052009223	0.178330038	salmon	1.1315733
GNAS-AS1	ENSG00000235590	0.84693897	0.7886081	0.358247354	0.17795148	purple	1.13127642
STK38	ENSG00000112079	3.90292705	0.43889084	0.049227837	0.177887484	purple	1.13122624
CHTOP	ENSG00000160679	8.04361922	0.18549618	0.004914768	0.17759955	purple	1.13100049
PFDN2	ENSG00000143256	6.73041151	0.23998064	0.009999847	0.177332301	purple	1.130791
APEX2	ENSG00000169188	5.79493466	0.2905218	0.016748822	0.176850321	salmon	1.13041328
NARS	ENSG00000134440	5.58008725	0.30746028	0.018880903	0.17650265	purple	1.1301409
SUV39H2	ENSG00000152455	3.14916335	0.50099716	0.077101063	0.176354945	salmon	1.1300252
DCAF6	ENSG00000143164	6.38483003	0.25749474	0.01208591	0.176117568	purple	1.12983928
ACBD6	ENSG00000230124	7.79483371	0.19363591	0.005616699	0.17607129	purple	1.12980304

PPP2R5A	ENSG00000066027	4.37575571	0.39701856	0.037394845	0.175821172	purple	1.12960719
EEF1AKNMT	ENSG00000010165	3.22032032	0.49563665	0.073856302	0.175680025	purple	1.12949668
ADAR	ENSG00000160710	5.12885409	0.34086768	0.024330185	0.175525855	purple	1.12937598
INTS3	ENSG00000143624	3.72873112	0.45392724	0.05453864	0.175348103	purple	1.12923684
PROSER3	ENSG00000167595	4.33309511	0.40018393	0.0383273	0.175235136	salmon	1.12914842
SMIM30	ENSG00000214194	2.48700991	0.56811192	0.115969249	0.175035812	purple	1.12899243
SHMT1	ENSG00000176974	1.48390719	0.69122869	0.224235531	0.174978829	salmon	1.12894784
LINC02055	ENSG00000254101	0.15971561	0.9259315	6.90E-01	0.17443369	royalblue	1.12852133
DNMT1	ENSG00000130816	4.37740731	0.39701856	0.03735923	0.174342064	salmon	1.12844966
PPOX	ENSG00000143224	4.23410577	0.40839302	0.040586737	0.174220049	purple	1.12835423
SMG5	ENSG00000198952	8.09978628	0.1845533	0.004769152	0.173778356	purple	1.12800883
NONO	ENSG00000147140	9.43472038	0.14573404	0.002348259	0.173403263	salmon	1.12771559
CSNK1A1P1	ENSG00000223518	0.47480587	0.85419972	4.91E-01	0.173139317	royalblue	1.12750929
XPR1	ENSG00000143324	4.34392222	0.39952821	0.038088348	0.17310596	purple	1.12748322
SLAMF9	ENSG00000162723	0.39389381	0.87159159	0.530794092	0.172429021	purple	1.12695431
GUK1	ENSG00000143774	6.14239718	0.27164468	0.013813946	0.172383766	purple	1.12691896
CEP135	ENSG00000174799	4.93899093	0.35380463	0.027092232	0.172194354	salmon	1.12677101
WDR34	ENSG00000119333	1.53985456	0.68309438	0.215724591	0.171895847	salmon	1.1265379
CALM2	ENSG00000143933	4.37016193	0.39703159	0.037515737	0.171038822	purple	1.12586888
CLN3	ENSG00000188603	4.08675564	0.42277171	0.04421431	0.170589777	purple	1.12551851
CACYBP	ENSG00000116161	3.86157927	0.44219437	0.050436576	0.170511529	purple	1.12545746
TMEM232	ENSG00000186952	1.19838909	0.73020178	0.274626752	0.170406388	purple	1.12537544
RBFA	ENSG00000101546	1.74118421	0.65464005	0.188114986	0.170166451	purple	1.1251883
UOX	ENSG00000240520	0.7031675	0.81438265	0.402468697	0.169708987	purple	1.12483157
NCAPD3	ENSG00000151503	3.16696064	0.50059499	0.076275306	0.169026738	salmon	1.12429976
ALDH9A1	ENSG00000143149	6.75587254	0.23910263	0.009861675	0.168685617	purple	1.12403396
NDC1	ENSG00000058804	3.67265663	0.46011676	0.056375507	0.168667484	salmon	1.12401983
POLE	ENSG00000177084	3.40185985	0.48385828	0.066226501	0.168571191	salmon	1.12394481

TMEM254-AS1	ENSG00000230091	1.15619642	0.73603301	0.283222847	0.168172262	purple	1.12363406
MSH5	ENSG00000204410	2.10598096	0.60741642	0.147893729	0.16781739	salmon	1.1233577
GOLGA8UP	ENSG00000103832	0.60027993	0.83030613	0.439154265	0.16738429	salmon	1.12302052
SNRPD3	ENSG00000100028	7.06575022	0.22336871	0.008329423	0.167367527	salmon	1.12300747
ABCB10	ENSG00000135776	4.26679925	0.40578619	0.039825346	0.167358189	purple	1.1230002
CABLES2	ENSG00000149679	2.78873566	0.53929505	0.096096612	0.167040746	salmon	1.12275313
THOC3	ENSG00000051596	3.00774349	0.51377784	0.084017432	0.16700024	salmon	1.12272161
SETDB1	ENSG00000143379	7.61890154	0.1995025	0.00617455	0.166144512	purple	1.12205587
MRTFB	ENSG00000186260	3.39805196	0.48418809	0.066377533	0.16588579	purple	1.12185467
NEB	ENSG00000183091	0.26605524	0.89717552	6.06E-01	0.165780797	royalblue	1.12177303
GLIS3	ENSG00000107249	0.16765741	0.92359931	0.682529256	0.165760481	midnightblue	1.12175723
SRPK2	ENSG00000135250	3.74386955	0.45294026	0.054053757	0.165683078	salmon	1.12169705
DDX59	ENSG00000118197	4.98800813	0.35070048	0.026349144	0.165532338	purple	1.12157985
RFC2	ENSG00000049541	4.25019406	0.40753574	0.040210157	0.165133228	salmon	1.12126962
MTBP	ENSG00000172167	2.82148238	0.53481631	0.094175773	0.16430747	salmon	1.12062802
HAUS8	ENSG00000131351	3.00308374	0.51450473	0.084256493	0.164156234	salmon	1.12051055
RAD21	ENSG00000164754	4.62141673	0.37762265	0.032470265	0.16399152	salmon	1.12038263
LIX1L-AS1	ENSG00000234222	3.130659	0.50154187	0.077969891	0.163790383	purple	1.12022644
LAGE3	ENSG00000196976	3.25704096	0.49280848	0.072239789	0.163593691	salmon	1.12007372
DCLRE1B	ENSG00000118655	1.63202337	0.66893149	0.202528966	0.163502246	salmon	1.12000273
SCAMP3	ENSG00000116521	4.93005811	0.35440889	0.027229996	0.161953407	purple	1.11880097
FLVCR1	ENSG00000162769	2.82539541	0.53411311	0.093949069	0.161800523	salmon	1.11868241
GON4L	ENSG00000116580	6.65623432	0.2428691	0.010413892	0.161432159	purple	1.11839682
HCG25	ENSG00000232940	1.83101024	0.64222656	0.177148576	0.161168043	purple	1.11819209
CCHCR1	ENSG00000204536	2.2314227	0.59728897	0.136405676	0.16109507	salmon	1.11813553
CRTC2	ENSG00000160741	5.52848698	0.31189603	0.019433789	0.161071215	purple	1.11811704
COX20	ENSG00000203667	4.63170297	0.37695764	0.032279568	0.160939838	purple	1.11801523
SLC39A1	ENSG00000143570	4.28086787	0.40433077	0.039502359	0.159955012	purple	1.1172523

UBE2Q1	ENSG00000160714	6.67182536	0.24238234	0.010325421	0.159913673	purple	1.11722029
MPC2	ENSG00000143158	4.29548809	0.40313237	0.039169637	0.159637116	purple	1.11700614
EPRS	ENSG00000136628	4.36849776	0.39703159	0.037551782	0.159503815	purple	1.11690294
CNIH4	ENSG00000143771	3.851945	0.44332724	0.050722726	0.158798979	purple	1.1163574
TRIM34	ENSG00000258659	0.77320278	0.80174941	0.380015809	0.158761373	midnightblue	1.1163283
NTAN1	ENSG00000157045	1.52551433	0.68604959	0.217868615	0.158728254	purple	1.11630268
EMC1	ENSG00000127463	5.66479703	0.29946864	0.018008441	0.158299859	salmon	1.11597125
NIT1	ENSG00000158793	3.98559831	0.43183058	0.046902362	0.15781373	purple	1.11559528
RNASEH1-AS1	ENSG00000234171	2.57643069	0.56045496	0.10964368	0.157333985	salmon	1.11522436
PITPNC1	ENSG00000154217	0.35302489	0.88103501	0.552907	0.157151429	purple	1.11508325
KIF20B	ENSG00000138182	1.55948921	0.67990861	0.21282975	0.15709564	salmon	1.11504014
L3MBTL2	ENSG00000100395	5.69509457	0.29735912	0.017706658	0.156717913	purple	1.11474823
SCLY	ENSG00000132330	5.43581373	0.31890247	0.020469457	0.156544599	salmon	1.11461432
RPRD2	ENSG00000163125	4.15255222	0.41639344	0.042553837	0.156338133	purple	1.11445482
PGP	ENSG00000184207	3.117485	0.5027251	0.078594901	0.156194078	salmon	1.11434355
TOPBP1	ENSG00000163781	4.82207445	0.36249493	0.028954583	0.156154128	salmon	1.11431269
MRPS14	ENSG00000120333	3.79282007	0.44868571	0.052517063	0.155263309	purple	1.11362485
DECR2	ENSG00000242612	1.96622727	0.62284996	0.162006106	0.155182866	purple	1.11356276
RO60	ENSG00000116747	3.47067275	0.47659107	0.063559819	0.155153924	purple	1.11354042
TOB1	ENSG00000141232	1.27866187	0.71770558	0.259158899	0.155019159	purple	1.1134364
PPP1R9B	ENSG00000108819	3.77637691	0.45023398	0.053028004	0.154948912	purple	1.11338219
COG2	ENSG00000135775	6.00861611	0.27697497	0.014875163	0.154363603	purple	1.11293058
EFNA3	ENSG00000143590	1.37145846	0.70454868	0.242601637	0.154217795	purple	1.1128181
TXNDC11	ENSG00000153066	2.73024652	0.54459375	0.099635134	0.154148907	purple	1.11276497
PIGC	ENSG00000135845	5.01083537	0.34882766	0.026010394	0.1540835	purple	1.11271452
SFT2D2	ENSG00000213064	2.95000751	0.51981544	0.087032306	0.153335995	purple	1.11213814
GVQW2	ENSG00000279968	0.91547849	0.77755077	0.339527283	0.153040508	purple	1.11191038
ZNF672	ENSG00000171161	4.10090427	0.42127384	0.043851549	0.152789009	purple	1.11171656

ISG20L2	ENSG00000143319	4.69889746	0.37257861	0.031062423	0.152323039	purple	1.11135755
FAM20B	ENSG00000116199	2.01020894	0.61912771	0.157405434	0.152128066	purple	1.11120737
CENPS	ENSG00000175279	2.19414323	0.59947152	0.139710959	0.151935045	salmon	1.1110587
ARNT	ENSG00000143437	2.94336128	0.52071329	0.087386847	0.151035172	purple	1.1103659
IQCH	ENSG00000103599	1.33243168	0.7111902	2.49E-01	0.150974616	royalblue	1.1103193
DHX9	ENSG00000135829	6.99930855	0.22623381	0.008635894	0.150222113	purple	1.10974031
RNF2	ENSG00000121481	4.03443396	0.42880063	0.045583656	0.150038087	purple	1.10959877
USP37	ENSG00000135913	3.3566185	0.48566885	0.068045039	0.149487519	salmon	1.1091754
LINC02475	ENSG00000251350	0.15062298	0.92828484	6.98E-01	0.149221041	royalblue	1.10897054
LRR1	ENSG00000165501	2.93299535	0.52220902	0.087942959	0.149110993	salmon	1.10888595
PAFAH2	ENSG00000158006	3.17130788	0.50016001	0.07607506	0.14904526	purple	1.10883543
CASP2	ENSG00000106144	5.32185126	0.32587603	0.021822341	0.148670233	salmon	1.10854723
RAB23	ENSG00000112210	1.19939684	0.7300274	0.274425501	0.148620364	purple	1.10850891
RAD9B	ENSG00000151164	0.98111958	0.76519652	0.322815998	0.148515638	salmon	1.10842844
GAS5-AS1	ENSG00000270084	2.13613313	0.60470443	0.145035628	0.148479819	purple	1.10840092
CCDC84-DT	ENSG00000255121	0.87845306	0.78217175	0.349469572	0.14830765	salmon	1.10826866
RPS6KC1	ENSG00000136643	2.00493824	0.62009015	0.157948812	0.147847999	purple	1.10791561
VPS72	ENSG00000163159	2.97843645	0.51776138	0.085533345	0.14784367	purple	1.10791229
IL23R	ENSG00000162594	0.14521625	0.92941883	7.03E-01	0.147717583	royalblue	1.10781547
NEMP1	ENSG00000166881	2.66554787	0.55115105	0.103716835	0.147355796	salmon	1.10753769
ZNF669	ENSG00000188295	2.13955184	0.60470443	0.14471554	0.14706924	purple	1.10731773
BROX	ENSG00000162819	2.78013953	0.54000731	0.096607919	0.146033976	purple	1.10652341
ADH6	ENSG00000172955	0.61047678	0.82980803	0.435298013	0.145903113	purple	1.10642305
MDH1	ENSG00000014641	3.58779398	0.46580028	0.059282127	0.145122841	purple	1.10582481
IFT81	ENSG00000122970	0.86960758	0.78429146	0.35190331	0.14405435	purple	1.10500611
TAF6	ENSG00000106290	5.21492511	0.33387744	0.023176537	0.143669063	purple	1.10471105
SMC1A	ENSG00000072501	3.0077787	0.51377784	0.084015629	0.143574518	salmon	1.10463866
TFDP1	ENSG00000198176	0.91291645	0.77773962	0.340202829	0.143569188	salmon	1.10463457

SMC4	ENSG00000113810	2.13834488	0.60470443	0.144828454	0.143462042	salmon	1.10455254
RNF115	ENSG00000265491	6.42727026	0.25542756	0.011807183	0.143459812	purple	1.10455083
WNK4	ENSG00000126562	0.7970675	0.79718297	0.372772532	0.143212164	purple	1.10436124
NUP85	ENSG00000125450	4.72165621	0.37199524	0.030661177	0.143072311	salmon	1.10425419
RNASEH2A	ENSG00000104889	2.13587854	0.60470443	0.145059497	0.142902341	salmon	1.1041241
GATAD2B	ENSG00000143614	4.24471202	0.4077106	0.04033806	0.142530287	purple	1.1038394
FDPS	ENSG00000160752	3.94842338	0.43495344	0.047933297	0.141826811	purple	1.10330129
ENSA	ENSG00000143420	3.49063904	0.47498599	0.062807808	0.141625631	purple	1.10314744
RBM15	ENSG00000162775	2.78160467	0.54000731	0.09652056	0.141486838	salmon	1.10304132
GGPS1	ENSG00000152904	3.20419714	0.49639137	0.074578372	0.141332214	purple	1.10292311
RAD18	ENSG00000070950	2.85146324	0.53085123	0.092453958	0.141273056	salmon	1.10287788
POGK	ENSG00000143157	3.46070035	0.47731539	0.06393903	0.140792674	purple	1.10251071
ZNF337-AS1	ENSG00000213742	2.09667846	0.60884136	0.148788361	0.140757621	salmon	1.10248393
RUSC1-AS1	ENSG00000225855	2.6337915	0.55360793	0.105787313	0.13954291	purple	1.10155605
MTMR8	ENSG00000102043	1.72717589	0.65580448	0.18989567	0.139536286	salmon	1.101551
SEPHS1	ENSG00000086475	4.01972398	0.42972672	0.045976682	0.139531136	salmon	1.10154706
UNG	ENSG00000076248	1.736764	0.6551839	0.188674748	0.13931391	salmon	1.10138122
CASP6	ENSG00000138794	2.09149439	0.60976752	0.149289584	0.139086778	purple	1.10120783
UBQLN4	ENSG00000160803	3.14664365	0.50132386	0.077218751	0.138973856	purple	1.10112164
PKIA	ENSG00000171033	0.37119203	0.87632174	0.542871359	0.138804387	purple	1.10099231
CDC73	ENSG00000134371	3.54957704	0.46940442	0.060643038	0.138560988	purple	1.10080657
RFC3	ENSG00000133119	1.09318733	0.74610168	0.296708185	0.138486697	salmon	1.10074989
MRPS21	ENSG00000266472	2.28322589	0.59318007	0.131957785	0.138485054	purple	1.10074863
GPR89B	ENSG00000188092	2.7798487	0.54000731	0.09662527	0.138129859	purple	1.10047766
COP1	ENSG00000143207	5.68523577	0.29813666	0.017804277	0.138126665	purple	1.10047522
PCDHB6	ENSG00000113211	0.19835189	0.91634979	6.56E-01	0.137333062	royalblue	1.09987004
WDR26	ENSG00000162923	4.26076502	0.40625943	0.039964732	0.137263238	purple	1.09981681
ESYT1	ENSG00000139641	2.22247379	0.59805814	0.137190995	0.137223313	purple	1.09978637

NDE1	ENSG00000072864	2.74374283	0.54426864	0.09880612	0.136259903	salmon	1.0990522
RIDA	ENSG00000132541	1.96114032	0.62334209	0.162548092	0.136119968	purple	1.0989456
SUCLG2-AS1	ENSG00000241316	0.63413224	0.82538052	0.426548445	0.136044047	purple	1.09888777
KLHL20	ENSG00000076321	5.02972855	0.34808795	0.025733469	0.136037093	purple	1.09888247
ZFP64	ENSG00000020256	2.22913465	0.59729492	0.136605983	0.136031976	purple	1.09887857
POGZ	ENSG00000143442	4.41962957	0.39508432	0.036460771	0.135831013	purple	1.09872551
MCM8	ENSG00000125885	1.47656226	0.69176438	0.225382596	0.13573656	salmon	1.09865358
POLR3C	ENSG00000186141	4.72447946	0.37199524	0.030611782	0.135021138	purple	1.0981089
JTB	ENSG00000143543	3.6634862	0.4610232	0.056682137	0.134865971	purple	1.0979908
CDH18	ENSG00000145526	0.12800102	0.93438486	7.21E-01	0.134854711	royalblue	1.09798223
ARF3	ENSG00000134287	3.36416694	0.48566885	0.067737926	0.134806358	purple	1.09794544
FDPSP1	ENSG00000225462	0.85120423	0.78797158	0.357041734	0.134566118	purple	1.09776262
TSNAX	ENSG00000116918	3.03192534	0.51175061	0.082788598	0.133966184	purple	1.09730622
HIST1H3PS1	ENSG00000220875	0.40234241	0.86977726	0.526423066	0.133899481	purple	1.09725548
SPATA5	ENSG00000145375	2.9556928	0.51958577	0.086730268	0.133726914	salmon	1.09712424
KIFAP3	ENSG00000075945	1.81460783	0.6448493	0.179094365	0.133726189	purple	1.09712369
TMEM241	ENSG00000134490	1.36494413	0.70603058	0.243720686	0.133365282	purple	1.09684927
FOXRED1	ENSG00000110074	3.90835539	0.43839071	0.049071448	0.133314692	salmon	1.09681081
GPRIN1	ENSG00000169258	0.52087539	0.84478975	0.4710977	0.132643851	salmon	1.09630092
SNX27	ENSG00000143376	2.35442989	0.58473472	0.12610721	0.132472361	purple	1.09617061
BEST4	ENSG00000142959	0.76474011	0.8028239	0.382632164	0.131983508	purple	1.09579924
SLC35B4	ENSG00000205060	1.30070326	0.71526076	0.255102969	0.131957911	salmon	1.0957798
PRPS1	ENSG00000147224	2.86456185	0.5285881	0.091712524	0.131727567	salmon	1.09560486
EFTUD2	ENSG00000108883	6.23336277	0.26756444	0.013137393	0.130335193	purple	1.09454798
EGLN1	ENSG00000135766	3.55813599	0.4685639	0.060335374	0.13031042	purple	1.09452918
DCAF8	ENSG00000132716	3.8678108	0.44197273	0.050252406	0.130236696	purple	1.09447325
TTC26	ENSG00000105948	0.87186748	0.78387141	0.351279325	0.130038945	purple	1.09432324
GORAB	ENSG00000120370	2.7360461	0.54459375	0.099277961	0.128728429	purple	1.09332963

KCNN3	ENSG00000143603	0.56288549	0.83719542	0.453758121	0.128595231	purple	1.09322869
ZBTB7B	ENSG00000160685	2.08685935	0.61057097	0.149739348	0.128377601	purple	1.09306379
YY1AP1	ENSG00000163374	4.40448862	0.39508432	0.036780313	0.128249713	purple	1.0929669
CPSF3	ENSG00000119203	3.65523647	0.4610232	0.056959503	0.127643289	salmon	1.09250758
GABPB2	ENSG00000143458	1.58345939	0.67559613	0.209358248	0.127353806	purple	1.09228839
SLC10A3	ENSG00000126903	1.95760743	0.62404066	0.162925725	0.126902864	purple	1.09194702
TSTD1	ENSG00000215845	1.78369536	0.648947	0.182829358	0.126566348	purple	1.09169235
STAU2	ENSG00000040341	3.24858702	0.49315782	0.072608536	0.125289327	purple	1.09072645
FLVCR1-DT	ENSG00000198468	0.58436486	0.83309617	0.445279047	0.124900041	salmon	1.09043218
MSH6	ENSG00000116062	2.70490158	0.54684345	0.101212669	0.12435745	salmon	1.09002215
OCRL	ENSG00000122126	1.59309077	0.67357589	0.207982379	0.12390875	salmon	1.08968319
C1orf216	ENSG00000142686	2.27626813	0.59374223	0.132545606	0.123889898	purple	1.08966895
KIAA1328	ENSG00000150477	1.69284293	0.6605164	0.194344119	0.123875414	salmon	1.08965801
CSTF2T	ENSG00000177613	2.21385293	0.59920488	0.137952345	0.123357033	purple	1.08926655
B3GALNT2	ENSG00000162885	2.22680308	0.59742056	0.136810441	0.121914983	purple	1.08817831
CYB5R1	ENSG00000159348	2.71975948	0.54579834	0.100284575	0.121863263	purple	1.0881393
CERS2	ENSG00000143418	2.74040319	0.54426864	0.099010551	0.121722255	purple	1.08803296
RELN	ENSG00000189056	0.04833937	0.9649722	8.26E-01	0.121310789	royalblue	1.08772269
FANCA	ENSG00000187741	0.73070609	0.80972767	0.393417913	0.121247802	salmon	1.0876752
TMEM237	ENSG00000155755	0.79221858	0.79855544	0.374228405	0.120320503	purple	1.08697631
ABHD3	ENSG00000158201	1.778827	0.6497468	0.183425802	0.120128241	salmon	1.08683147
ZNF692	ENSG00000171163	1.8478266	0.64008754	0.175179081	0.119902184	purple	1.08666118
ANGEL2	ENSG00000174606	3.76338176	0.45166324	0.053435549	0.119686378	purple	1.08649865
PMVK	ENSG00000163344	1.39278134	0.70191046	0.238982401	0.119552468	purple	1.0863978
ADIPOR1	ENSG00000159346	3.6090582	0.46451731	0.058539079	0.119127511	purple	1.08607784
PGM2	ENSG00000169299	2.72028265	0.54579834	0.100252067	0.118876559	purple	1.08588894
GPR89A	ENSG00000117262	2.23932843	0.5964725	0.13571611	0.118735941	purple	1.08578311
USP5	ENSG00000111667	2.47436489	0.56991435	0.116895956	0.118214236	purple	1.08539054

PRR3	ENSG00000204576	3.43096674	0.48093087	0.06508416	0.118194506	salmon	1.08537569
TPP2	ENSG00000134900	1.14638741	0.73744468	0.285269972	0.118192576	midnightblue	1.08537424
THBS3	ENSG00000169231	1.16829028	0.73409181	0.280724499	0.118019067	purple	1.08524371
MAZ	ENSG00000103495	3.48529668	0.47551454	0.063008083	0.117858167	purple	1.08512269
SSR2	ENSG00000163479	2.3484492	0.58588681	0.126587288	0.117603774	purple	1.08493136
EHMT2	ENSG00000204371	3.29483271	0.49118983	0.070615887	0.117556126	salmon	1.08489553
CCDC102B	ENSG00000150636	0.49713438	0.84959223	0.481374288	0.117316491	purple	1.08471534
TOP3A	ENSG00000177302	4.09889244	0.42150678	0.043902938	0.11710626	salmon	1.08455729
VPS45	ENSG00000136631	2.082461	0.61125812	0.150167569	0.116584899	purple	1.08416542
TACO1	ENSG00000136463	3.62646851	0.4624797	0.057938113	0.116438399	purple	1.08405533
DSTYK	ENSG00000133059	2.42597826	0.57575476	0.120518801	0.116397551	purple	1.08402464
TSN	ENSG00000211460	4.85180291	0.35980363	0.028468677	0.116026686	salmon	1.08374601
TRIM59	ENSG00000213186	1.39932662	0.70125419	0.237884679	0.115761418	salmon	1.08354676
GALNT2	ENSG00000143641	2.26626761	0.59374223	0.133395648	0.115695374	purple	1.08349716
GATB	ENSG00000059691	2.09431098	0.60933087	0.149017024	0.115379292	purple	1.0832598
KCND1	ENSG00000102057	0.55142419	0.83921001	0.45838672	0.115321148	purple	1.08321615
RPL21P110	ENSG00000226617	0.26966878	0.89695824	0.603982628	0.114742545	midnightblue	1.0827818
VCX3A	ENSG00000169059	0.22473041	0.90753271	6.36E-01	0.114333563	royalblue	1.08247489
EMC3-AS1	ENSG00000180385	1.0557034	0.75316014	0.305123638	0.113992623	salmon	1.08221911
MGME1	ENSG00000125871	2.07895631	0.61146048	0.15050978	0.113795203	salmon	1.08207103
NASP	ENSG00000132780	1.48561512	0.69115522	0.223969811	0.11348794	salmon	1.0818406
TXNRD1	ENSG00000198431	2.4020175	0.57874569	0.122358936	0.11243443	purple	1.08105088
MTR	ENSG00000116984	2.30737999	0.59109412	0.129939725	0.112347705	purple	1.0809859
TRMT61B	ENSG00000171103	3.61549335	0.46382414	0.058316179	0.11112858	purple	1.08007282
NLRX1	ENSG00000160703	1.36903281	0.70522118	0.243017586	0.110754976	salmon	1.07979316
CEP97	ENSG00000182504	1.02218007	0.75831966	0.312914368	0.110565678	salmon	1.07965148
CNOT9	ENSG00000144580	4.64931686	0.37589062	0.031955749	0.110510329	salmon	1.07961006
NENF	ENSG00000117691	1.02607078	0.75747701	0.311996951	0.110305708	purple	1.07945695

CEP78	ENSG00000148019	1.71933155	0.65718918	0.190901429	0.110013043	salmon	1.07923799
RALBP1	ENSG00000017797	1.81493415	0.64478493	0.179055413	0.109906223	purple	1.07915809
DENND1B	ENSG00000213047	0.97782981	0.76613339	0.323627108	0.109130508	purple	1.078578
MCM7	ENSG00000166508	1.79837042	0.64722713	0.181045067	0.109105851	salmon	1.07855956
PRDX3	ENSG00000165672	1.53281127	0.6847834	0.21677447	0.108965206	purple	1.07845442
TAF4	ENSG00000130699	2.8437641	0.53167057	0.092892815	0.108928872	salmon	1.07842726
BPNT1	ENSG00000162813	1.27603846	0.71779001	0.259646953	0.108643956	purple	1.07821431
ZNF687	ENSG00000143373	2.07804793	0.61146048	0.150598622	0.107669063	purple	1.07748595
POLA2	ENSG00000014138	1.28711535	0.71610189	0.257593951	0.107163492	salmon	1.07710843
TTLL4	ENSG00000135912	1.88906027	0.63344614	0.170456153	0.106985987	salmon	1.07697592
HNRNPR	ENSG00000125944	4.67081257	0.37420821	0.03156518	0.106348088	salmon	1.07649983
CEP350	ENSG00000135837	2.39495163	0.57928604	0.122907542	0.10610764	purple	1.07632043
NTPCR	ENSG00000135778	1.62032887	0.67079056	0.204149054	0.105373074	purple	1.07577254
FMO4	ENSG00000076258	0.56685184	0.83634188	0.452173465	0.105093635	purple	1.0755642
TPR	ENSG00000047410	2.02329146	0.6176497	0.156065904	0.105019642	salmon	1.07550903
VRK1	ENSG00000100749	1.1479326	0.73734191	0.284946246	0.104981667	salmon	1.07548072
STK35	ENSG00000125834	2.98509459	0.5169623	0.085186359	0.10486857	salmon	1.07539642
POLR3GL	ENSG00000121851	2.32731834	0.58821115	0.128299928	0.104798804	purple	1.07534441
MRPS28	ENSG00000147586	1.41051989	0.70026082	0.236021631	0.104686365	purple	1.07526061
PRRC2A	ENSG00000204469	2.30221643	0.59109412	0.130368213	0.104282646	salmon	1.07495975
MPV17	ENSG00000115204	3.53741032	0.47068303	0.061083283	0.104171182	purple	1.0748767
FAM189B	ENSG00000160767	0.80159886	0.79623655	0.371419188	0.103807107	purple	1.07460549
NAXE	ENSG00000163382	1.32436694	0.71136012	0.250835562	0.103323006	purple	1.07424496
LINC02328	ENSG00000258733	0.21477009	0.91069572	0.643430207	0.103249568	midnightblue	1.07419028
MACROD2	ENSG00000172264	0.08724443	0.95071206	0.767938678	0.102793473	midnightblue	1.07385074
MED24	ENSG00000008838	3.0505752	0.50943837	0.08185421	0.102584703	purple	1.07369535
IVNS1ABP	ENSG00000116679	1.85959349	0.63785359	0.17381604	0.102390926	purple	1.07355115
DNAJC11	ENSG00000007923	5.3139965	0.32638305	0.021918954	0.102049953	salmon	1.07329745

CACYBPP2	ENSG00000177855	0.603921	0.83017503	0.437771286	0.101793852	purple	1.07310694
DECR1	ENSG00000104325	1.75839396	0.65340429	0.185954006	0.101656003	purple	1.07300441
ICMT	ENSG00000116237	1.79657014	0.64732506	0.181262863	0.101591915	purple	1.07295674
PYGO2	ENSG00000163348	1.4631404	0.69319325	0.22749703	0.101025739	purple	1.07253575
MGST1	ENSG00000008394	0.04545607	0.96558956	0.831329714	0.100828481	purple	1.07238912
TOR1AIP2	ENSG00000169905	1.20443856	0.72922962	0.273421427	0.100588422	purple	1.07221069
PDCL3	ENSG00000115539	0.93068249	0.77378267	0.335555337	0.100434785	purple	1.07209651
KIF3B	ENSG00000101350	1.85014321	0.64008754	0.174909756	0.099903012	purple	1.07170141
THEM4	ENSG00000159445	1.12894975	0.74002101	0.288955957	0.098908704	purple	1.07096305
ENTPD1-AS1	ENSG00000226688	1.25000958	0.72081544	0.264551845	0.098869613	salmon	1.07093403
EFCAB11	ENSG00000140025	1.18873203	0.73140272	0.276564789	0.098860055	salmon	1.07092694
LIFR	ENSG00000113594	0.04908204	0.96446248	0.824837073	0.09868914	purple	1.07080007
SNAPIN	ENSG00000143553	1.39318686	0.70183596	0.238914211	0.098442396	purple	1.07061695
HAX1	ENSG00000143575	1.50583353	0.68983817	0.220852823	0.097788039	purple	1.07013146
UFC1	ENSG00000143222	1.39051221	0.7022642	0.239364403	0.097386312	purple	1.06983352
CNTROB	ENSG00000170037	1.46357455	0.69319325	0.227428261	0.097380737	purple	1.06982939
HNRNPU	ENSG00000153187	2.99935991	0.51513996	0.08444807	0.097153261	purple	1.06966071
TTL	ENSG00000114999	1.33482799	0.71071954	0.248977147	0.096633232	salmon	1.06927522
ATL2	ENSG00000119787	2.81315534	0.5362034	0.094660199	0.096620353	salmon	1.06926567
NELFCD	ENSG00000101158	2.23215115	0.59728897	0.136341973	0.096365187	salmon	1.06907657
RNU6-850P	ENSG00000252743	0.27527087	0.89518079	0.600251448	0.095982148	salmon	1.06879277
RFC5	ENSG00000111445	0.98677122	0.76423242	0.321428829	0.095659749	salmon	1.06855395
XRCC3	ENSG00000126215	1.04936942	0.7535391	0.306576101	0.095165831	salmon	1.06818818
PI4KB	ENSG00000143393	2.20657016	0.59928233	0.138599232	0.094925372	purple	1.06801016
C1orf43	ENSG00000143612	1.74146787	0.65464005	0.18807913	0.094359255	purple	1.06759115
TMEM67	ENSG00000164953	0.26376958	0.89736263	0.607965303	0.094197303	purple	1.06747132
PRORP	ENSG00000100890	1.04359947	0.75432976	0.307907066	0.094077174	salmon	1.06738243
SMAD2	ENSG00000175387	2.49869423	0.56630991	0.115120192	0.093897084	salmon	1.0672492

EXTL2	ENSG00000162694	0.39055342	0.87244579	5.33E-01	0.093664777	royalblue	1.06707737
UBR7	ENSG00000012963	1.75081462	0.65406274	0.186902129	0.093654565	salmon	1.06706981
GLB1L	ENSG00000163521	0.87290368	0.78355527	0.350993724	0.093382044	purple	1.06686826
UCHL5	ENSG00000116750	1.52192056	0.68697763	0.218409914	0.093152907	purple	1.06669883
AK2	ENSG00000004455	2.95773658	0.51958577	0.086621968	0.092829688	purple	1.06645988
MRGBP	ENSG00000101189	2.11898489	0.6067668	0.146653338	0.092778283	purple	1.06642188
UBL7-AS1	ENSG00000247240	0.99766871	0.76213162	3.19E-01	0.092777846	royalblue	1.06642156
VEGFA	ENSG00000112715	0.50223942	0.84855792	0.479133772	0.092083656	purple	1.06590854
PSMD10	ENSG00000101843	1.2840507	0.71680379	0.258159936	0.092036591	purple	1.06587377
FAM86B2	ENSG00000145002	0.17325253	0.9226246	0.677570347	0.091606793	purple	1.06555628
PSMD11	ENSG00000108671	2.30494174	0.59109412	0.130141861	0.091576787	purple	1.06553412
ANKRD20A17P	ENSG00000251056	0.09652324	0.94725137	7.56E-01	0.091527896	royalblue	1.06549801
MPHOSPH9	ENSG00000051825	1.38742181	0.70273122	0.239885861	0.090563788	salmon	1.06478621
PPP1R12B	ENSG00000077157	1.48258624	0.69122869	0.224441305	0.090427684	purple	1.06468576
YTHDF2	ENSG00000198492	2.29979011	0.59109412	0.130570102	0.09002374	purple	1.0643877
RNASEH1	ENSG00000171865	2.46477327	0.5706914	0.117604377	0.089622322	salmon	1.06409158
PIGU	ENSG00000101464	1.5346325	0.6842827	0.216502411	0.089419829	salmon	1.06394224
TAF1B	ENSG00000115750	1.21793173	0.72652865	0.270756866	0.089149793	salmon	1.06374311
MR1	ENSG00000153029	1.13665209	0.73893768	0.287320384	0.088971209	purple	1.06361145
NUDT1	ENSG00000106268	0.58175779	0.8338524	0.446294959	0.088970645	salmon	1.06361103
PPP1CB	ENSG00000213639	1.82828476	0.64278324	0.177470189	0.088965264	purple	1.06360706
SMYD2	ENSG00000143499	1.06910918	0.75036606	0.302078867	0.088137692	purple	1.06299712
PSMD12	ENSG00000197170	1.85421017	0.6392164	0.174438099	0.087969654	purple	1.06287332
C5orf34	ENSG00000172244	0.4425249	0.86181877	0.506478371	0.087531205	salmon	1.06255035
BFSP2-AS1	ENSG00000249993	0.07292397	0.95590789	0.787333934	0.086481829	midnightblue	1.06177776
ITGB3BP	ENSG00000142856	1.09547024	0.74570399	0.296205395	0.086091699	salmon	1.06149068
NEK7	ENSG00000151414	0.8557094	0.78741294	0.355774363	0.08591753	purple	1.06136254
KDSR	ENSG00000119537	1.03882159	0.75508246	0.309014891	0.083672376	purple	1.0597121

BORCS5	ENSG00000165714	1.03532502	0.75561896	0.309828923	0.083478832	purple	1.05956995
XPNPEP3	ENSG00000196236	1.20824216	0.72871557	0.272666988	0.081898707	purple	1.05841008
RNU1-51P	ENSG00000202215	0.04446913	0.96620571	8.33E-01	0.081472533	royalblue	1.05809747
DAAM1	ENSG00000100592	0.38044169	0.87493476	0.537890562	0.081188748	salmon	1.05788936
AHCTF1	ENSG00000153207	1.25428783	0.71994364	0.263737771	0.081148376	purple	1.05785976
MIA3	ENSG00000154305	1.00279547	0.76109118	0.317538322	0.081146594	purple	1.05785845
UROS	ENSG00000188690	1.74858992	0.65427743	0.187181494	0.080696897	purple	1.05752876
RGS5	ENSG00000143248	0.45364651	0.85929662	0.501188788	0.080291313	purple	1.0572315
MKRN3	ENSG00000179455	0.03842805	0.96829214	8.45E-01	0.080022111	royalblue	1.05703424
ACAT2	ENSG00000120437	0.75927412	0.80387608	0.38433568	0.079855405	salmon	1.05691211
CANT1	ENSG00000171302	1.73780287	0.65508516	0.188543014	0.078415571	purple	1.05585782
RAB22A	ENSG00000124209	1.52898684	0.68546554	0.217347104	0.07836791	purple	1.05582294
RAD54B	ENSG00000197275	0.25768605	0.89887833	0.612131845	0.078103457	salmon	1.05562942
REEP4	ENSG00000168476	0.6352726	0.82538052	0.426133409	0.077796605	salmon	1.05540491
SEH1L	ENSG00000085415	1.05533867	0.75318371	0.305207032	0.077104353	salmon	1.05489862
CEP131	ENSG00000141577	1.0205511	0.75841939	0.313299524	0.077077771	salmon	1.05487918
APOBEC3C	ENSG00000244509	0.63961771	0.82464023	0.424557561	0.076699445	purple	1.05460259
CACUL1	ENSG00000151893	1.61392291	0.67138348	0.205043003	0.075614599	purple	1.05380987
ZBED4	ENSG00000100426	1.10297859	0.74520832	0.294559456	0.075148022	purple	1.05346912
LYPLAL1	ENSG00000143353	0.67144928	0.81912871	0.413274881	0.074957814	purple	1.05333024
SLBP	ENSG00000163950	0.93912968	0.77245232	0.33337552	0.074725273	salmon	1.05316047
GLUD1	ENSG00000148672	1.99906376	0.620491	0.158556966	0.074205734	purple	1.05278127
NPRL3	ENSG00000103148	0.91064331	0.77785411	0.340803718	0.073742665	purple	1.05244341
XRCC6	ENSG00000196419	1.40158622	0.70125419	0.237507145	0.073238908	purple	1.05207599
GAR1	ENSG00000109534	1.22733391	0.72522476	0.268919434	0.0731269	purple	1.05199431
NUMBL	ENSG00000105245	0.25410185	0.8998548	0.614615679	0.073122481	salmon	1.05199109
DYRK4	ENSG00000010219	0.73498465	0.80892108	0.392038208	0.07203522	purple	1.05119857
GNAI3	ENSG00000065135	0.96229921	0.76808299	0.32749299	0.070568466	purple	1.05013039

RPL31P44	ENSG00000213657	0.04337205	0.96662337	0.835184477	0.07039953	midnightblue	1.05000743
PATZ1	ENSG00000100105	0.57916803	0.83414975	0.447307699	0.070298873	midnightblue	1.04993417
NSF	ENSG00000073969	0.92555696	0.77515704	0.336887327	0.069450062	purple	1.04931662
ZC4H2	ENSG00000126970	0.18984564	0.91770482	0.663395851	0.068399267	purple	1.04855262
ATE1	ENSG00000107669	1.06088295	0.75242424	0.30394254	0.067666341	purple	1.04802007
TOR1AIP1	ENSG00000143337	0.97014136	0.76691993	0.325533299	0.067425925	purple	1.04784544
LINC00634	ENSG00000205704	0.10962633	0.94133033	0.740828689	0.066803364	salmon	1.04739336
MASTL	ENSG00000120539	0.65553079	0.82082057	0.418860462	0.066776488	salmon	1.04737385
RBKS	ENSG00000171174	0.49924259	0.84916334	0.480446949	0.066655803	purple	1.04728624
MBLAC1	ENSG00000214309	0.46396289	0.85712651	0.496365835	0.066536207	purple	1.04719942
WDR81	ENSG00000167716	0.76880608	0.80250453	0.38137193	0.066440822	purple	1.04713019
EIF3EP1	ENSG00000234882	0.34873045	0.88176667	0.55533031	0.066103101	salmon	1.04688509
SASS6	ENSG00000156876	0.48706027	0.85181319	0.485846643	0.06602739	salmon	1.04683016
GTF3C2	ENSG00000115207	2.27799046	0.59374223	0.132399823	0.065885466	purple	1.04672718
CEP76	ENSG00000101624	0.64625593	0.82301361	0.422166946	0.065446519	salmon	1.04640876
TM7SF3	ENSG00000064115	0.51550134	0.84598158	0.473392425	0.064235125	purple	1.04553048
H2AFV	ENSG00000105968	1.01144915	0.75944448	0.315463082	0.06370401	salmon	1.04514565
POFUT1	ENSG00000101346	0.92383617	0.77531713	0.33733611	0.06358444	salmon	1.04505903
MEMO1	ENSG00000162959	1.11979537	0.74228058	0.290915379	0.063107555	salmon	1.04471365
TELO2	ENSG00000100726	0.62905782	0.82602978	0.428402723	0.062965775	salmon	1.04461098
MTPAP	ENSG00000107951	1.4065573	0.700841	0.236679141	0.06211894	salmon	1.04399799
EIF4E2	ENSG00000135930	2.35951712	0.58449931	0.125700454	0.062054575	purple	1.04395142
DDX52	ENSG00000278053	1.44893849	0.69500456	0.229760491	0.061999271	salmon	1.0439114
XPO1	ENSG00000082898	0.81052907	0.79473309	0.368772146	0.06115339	salmon	1.04329951
THOC5	ENSG00000100296	0.78532942	0.79983002	0.376310641	0.059926354	salmon	1.04241255
SMYD3	ENSG00000185420	0.1727519	0.92262839	0.67801019	0.059884723	purple	1.04238247
SUZ12	ENSG00000178691	1.11537235	0.74330736	0.291868185	0.059848996	salmon	1.04235665
USP21	ENSG00000143258	0.74809402	0.80522297	0.387853877	0.05931539	purple	1.04197119

STX2	ENSG00000111450	0.2490475	0.90068667	0.618155952	0.058246426	purple	1.04119943
TRMT6	ENSG00000089195	0.7496771	0.80511517	0.387352918	0.057412564	salmon	1.0405978
ENY2	ENSG00000120533	1.11944497	0.742349	0.290990717	0.05727234	purple	1.04049667
NCOA7-AS1	ENSG00000232131	0.0640142	0.95935364	0.800454229	0.056480537	midnightblue	1.03992576
AMER1	ENSG00000184675	0.38860991	0.87304303	0.533561166	0.05623123	salmon	1.03974607
STX6	ENSG00000135823	0.44823143	0.86092874	0.503752392	0.055866964	purple	1.03948358
SPECC1L	ENSG00000100014	0.32616021	0.88471069	0.56840748	0.055526093	salmon	1.039238
SLC16A13	ENSG00000174327	0.20138598	0.91564117	0.653967079	0.055149468	purple	1.03896674
AFG3L2	ENSG00000141385	0.71620798	0.81191	0.398145597	0.053932917	salmon	1.038091
SMC6	ENSG00000163029	0.41556053	0.86648911	0.519712001	0.053902206	salmon	1.0380689
KPNA3	ENSG00000102753	0.47319305	0.85453663	0.492116805	0.053293142	midnightblue	1.03763075
EMG1	ENSG00000126749	0.7921716	0.79855544	0.37424255	0.052372766	purple	1.036969
SNAP29	ENSG00000099940	0.81930671	0.79354556	0.36619594	0.052130877	purple	1.03679515
CENPJ	ENSG00000151849	0.12643436	0.93527186	0.722437754	0.051897411	midnightblue	1.03662739
ACYP1	ENSG00000119640	0.24872554	0.90072109	0.618382978	0.050209703	salmon	1.03541542
DNAJC9	ENSG00000213551	0.81777408	0.7940106	0.366643948	0.049997389	salmon	1.03526305
XRCC5	ENSG00000079246	0.88134347	0.78214782	0.34867929	0.049773939	salmon	1.03510272
JUP	ENSG00000173801	0.01625868	0.98148724	0.89863275	0.049589011	purple	1.03497004
CHD4	ENSG00000111642	0.64403	0.82364431	0.422966308	0.04951053	salmon	1.03491374
CATSPER1	ENSG00000175294	0.0252297	0.9743729	0.873915671	0.048984606	purple	1.03453654
LINC00894	ENSG00000235703	0.1036676	0.9448676	0.747722993	0.048314132	midnightblue	1.03405587
SACS	ENSG00000151835	0.22476795	0.90753271	0.635816773	0.048074992	midnightblue	1.03388448
MTRF1	ENSG00000120662	0.27877677	0.89398949	0.597940979	0.047682586	midnightblue	1.0336033
TATDN3	ENSG00000203705	0.24622447	0.90170302	0.620152895	0.046536243	purple	1.03278235
DPY30	ENSG00000162961	0.4691822	0.85593706	0.493955615	0.045271079	purple	1.03187705
FAM210A	ENSG00000177150	0.36707593	0.87731097	0.545115242	0.045271057	salmon	1.03187703
KLHDC3	ENSG00000124702	0.44777334	0.8610394	0.503970288	0.044740643	purple	1.03149773
ZNF519	ENSG00000175322	0.09562167	0.94799931	0.757388724	0.044447214	salmon	1.03128795

AIDA	ENSG00000186063	0.28776876	0.89172985	0.592098919	0.042480418	purple	1.02988298
FAM86EP	ENSG00000251669	0.13020636	0.93368186	0.718502433	0.042057453	purple	1.02958108
MX2	ENSG00000183486	0.02780278	0.97316346	0.867699054	0.041807135	midnightblue	1.02940246
TMEM53	ENSG00000126106	0.27202685	0.89636791	0.602406122	0.041789862	purple	1.02939014
TMTC4	ENSG00000125247	0.15001934	0.92837192	0.698824285	0.041333045	midnightblue	1.02906424
ATP9B	ENSG00000166377	0.40240684	0.86977726	0.526389982	0.041125478	salmon	1.02891619
ACAA2	ENSG00000167315	0.29467889	0.89037729	0.587688832	0.040568677	salmon	1.02851917
SLC20A2	ENSG00000168575	0.42485549	0.86457169	0.515082724	0.040253967	purple	1.02829483
G3BP2	ENSG00000138757	0.39496908	0.87156084	0.530234166	0.039879457	purple	1.02802793
PLEKHB2	ENSG00000115762	0.2765538	0.89474798	0.599403801	0.038910454	purple	1.02733767
SYT2	ENSG00000143858	0.13707148	0.93136129	0.711502314	0.038816162	purple	1.02727053
LAS1L	ENSG00000001497	0.63364213	0.8253994	0.426727012	0.03759783	salmon	1.02640338
TERT	ENSG00000164362	0.03680775	0.96911153	0.848002281	0.037433921	salmon	1.02628678
HAUS4	ENSG00000092036	0.20653116	0.91413916	0.649867801	0.037261218	purple	1.02616393
NUFIP1	ENSG00000083635	0.16204612	0.92527665	0.687600549	0.035519136	midnightblue	1.02492556
GLRX3	ENSG00000108010	0.43395476	0.86312916	0.510620316	0.034926123	purple	1.02450436
PRDX6	ENSG00000117592	0.2291652	0.90655381	0.632533832	0.034805732	purple	1.02441887
KLF12	ENSG00000118922	0.02792651	0.97316346	0.867407738	0.034343828	midnightblue	1.02409094
HBBP1	ENSG00000229988	0.00551327	0.98888258	9.41E-01	0.033365237	royalblue	1.02339652
KHDC1	ENSG00000135314	0.07005327	0.95761918	0.791462683	0.033282111	salmon	1.02333756
HIBCH	ENSG00000198130	0.13292992	0.93271305	0.715700851	0.032943928	purple	1.02309771
NFYAP1	ENSG00000237849	0.00958359	0.98640196	9.22E-01	0.031455412	royalblue	1.02204266
SCIN	ENSG00000006747	0.0080869	0.98764238	0.928412238	0.031255023	midnightblue	1.02190071
BCKDHB	ENSG00000083123	0.10035785	0.94611631	0.751647256	0.031015012	purple	1.02173071
FAM155B	ENSG00000130054	0.01635541	0.98148724	0.89833331	0.030569343	purple	1.02141514
CCDC18	ENSG00000122483	0.08930549	0.94998029	0.765293543	0.029134759	salmon	1.02039997
TMEM254	ENSG00000133678	0.13082053	0.93368186	0.717867801	0.029025259	purple	1.02032252
RPRD1A	ENSG00000141425	0.19716842	0.91634979	0.657374508	0.028733347	salmon	1.02011609

RITA1	ENSG00000139405	0.1465305	0.92878304	0.702176657	0.028531513	purple	1.01997339
UXS1	ENSG00000115652	0.11365148	0.93968536	0.736288534	0.028367889	purple	1.01985772
BORA	ENSG00000136122	0.05069948	0.96357638	0.822022166	0.025614532	midnightblue	1.01791319
DNM1L	ENSG00000087470	0.14685132	0.92878011	7.02E-01	0.025293164	royalblue	1.01768647
HADHB	ENSG00000138029	0.19964922	0.91615763	0.655364996	0.025177854	purple	1.01760513
PDHA1	ENSG00000131828	0.23360873	0.90597416	0.62925546	0.024657763	purple	1.01723835
ZNF711	ENSG00000147180	0.00768505	0.98797393	0.930208849	0.024597593	midnightblue	1.01719593
DLEU2	ENSG00000231607	0.03123644	0.97150705	0.859847416	0.0245888	midnightblue	1.01718973
RCBTB1	ENSG00000136144	0.07082114	0.95740591	0.790349559	0.02453567	midnightblue	1.01715227
MCMBP	ENSG00000197771	0.12882407	0.93390463	0.719937008	0.024153888	salmon	1.01688314
PLA2G4A	ENSG00000116711	0.01799899	0.97984531	0.893376507	0.024052953	purple	1.01681199
ZDHHC20	ENSG00000180776	0.06448132	0.95900375	0.799743012	0.023771812	midnightblue	1.01661387
UBA1	ENSG00000130985	0.18530786	0.91891245	0.667196858	0.023759178	purple	1.01660496
CNOT10	ENSG00000182973	0.1773279	0.92137749	0.674017246	0.022087072	salmon	1.01542738
TMEM231	ENSG00000205084	0.02286911	0.97612714	0.879911714	0.022068359	purple	1.01541421
HNRNPA1P46	ENSG00000228020	0.00399224	0.99050449	9.50E-01	0.019204714	royalblue	1.01340069
MTTP	ENSG00000138823	0.00767872	0.98797393	0.930237531	0.018740609	purple	1.01307474
TCFL5	ENSG00000101190	0.00693615	0.98856074	0.933688249	0.018558202	purple	1.01294666
NUP58	ENSG00000139496	0.05314922	0.96285715	0.817847189	0.018544858	midnightblue	1.01293729
HSD17B7	ENSG00000132196	0.04944153	0.9642771	0.824207269	0.017897145	purple	1.01248262
FKBP4	ENSG00000004478	0.03758697	0.96858771	0.846421734	0.017634932	purple	1.01229862
LMO7	ENSG00000136153	0.02312881	0.97596902	0.879237005	0.017481459	midnightblue	1.01219094
PELP1	ENSG00000141456	0.06907345	0.95812306	0.792892572	0.017239555	purple	1.01202123
PRDM11	ENSG00000019485	0.01153645	0.9856621	0.914545787	0.016855306	midnightblue	1.01175172
NRIR	ENSG00000225964	0.00219586	0.99278989	0.962659697	0.016518082	midnightblue	1.01151526
TMEM14B	ENSG00000137210	0.0653706	0.95884537	0.798396578	0.016305476	purple	1.01136621
PDS5B	ENSG00000083642	0.03275128	0.9703608	0.856525446	0.016137973	midnightblue	1.01124879
MRPL57	ENSG00000173141	0.04403594	0.96624007	0.8339462	0.016064131	midnightblue	1.01119703

PAK2	ENSG00000180370	0.08444838	0.9521328	0.771581985	0.01579026	salmon	1.01100509
LINC01232	ENSG00000280734	0.0103246	0.98596532	0.919142209	0.015667661	midnightblue	1.01091918
CPSF6	ENSG00000111605	0.09746049	0.94697538	0.755141447	0.015343564	salmon	1.01069211
PARP8	ENSG00000151883	0.01553576	0.9820624	0.900900005	0.015338377	midnightblue	1.01068847
EPHX1	ENSG00000143819	0.01755763	0.98032143	0.89468415	0.015317623	purple	1.01067393
ATXN10	ENSG00000130638	0.04552249	0.96554096	0.831208393	0.014689924	purple	1.0102343
RPL23AP82	ENSG00000184319	0.01982685	0.97879534	0.888127511	0.014240024	purple	1.00991931
PWP1	ENSG00000136045	0.06004304	0.96003316	0.806615448	0.013991231	purple	1.00974516
CCDC187	ENSG00000260220	0.00041247	0.99649306	9.84E-01	0.010238651	royalblue	1.00712214
EXOSC8	ENSG00000120699	0.01264916	0.98433828	0.910536151	0.010138554	midnightblue	1.00705226
NPC1	ENSG00000141458	0.02265278	0.97635334	0.880476739	0.010046362	salmon	1.00698791
DDX1	ENSG00000079785	0.01764434	0.98031692	0.894425932	0.008298916	purple	1.00576895
RNLS	ENSG00000184719	0.00912353	0.98682792	0.923975449	0.007291811	purple	1.00506709
AMMECR1	ENSG00000101935	0.00514853	0.98905101	0.942851789	0.007148535	salmon	1.00496728
ODR4	ENSG00000157181	0.00619613	0.98872623	0.937317675	0.005914828	purple	1.00410826
LINC02363	ENSG00000180712	0.00086005	0.99464982	0.97662593	0.00551021	midnightblue	1.00382669
TCF20	ENSG00000100207	0.00411141	0.99033395	0.948922259	0.004791662	salmon	1.00332685
KLHL3	ENSG00000146021	0.00123939	0.99423295	9.72E-01	0.004789491	royalblue	1.00332534
TRIM47	ENSG00000132481	0.00052114	0.99579187	0.981804145	0.003910437	purple	1.00271419
WDR90	ENSG00000161996	0.00068185	0.99511037	0.979187292	0.003731137	salmon	1.00258957
SLC39A6	ENSG00000141424	0.00191673	0.99335896	0.965112026	0.003635046	purple	1.0025228
ZNF768	ENSG00000169957	0.00296035	0.99185364	0.956649747	0.003095188	purple	1.00214772
ZNF18	ENSG00000154957	0.00214545	0.99278989	0.963090519	0.002866186	purple	1.00198866
ALG11	ENSG00000253710	0.0009285	0.99464982	0.975713912	0.002617007	midnightblue	1.00181562
PTPRT	ENSG00000196090	1.69E-05	0.99877753	9.97E-01	0.001487449	royalblue	1.00103155
DCLRE1C	ENSG00000152457	0.00022606	0.99713988	0.988015129	0.00106857	salmon	1.00074095
RETREG2	ENSG00000144567	0.00015208	0.99755692	0.990169987	0.000703321	purple	1.00048762
SLC25A13	ENSG00000004864	5.23E-05	0.99837824	9.94E-01	-0.000500755	royalblue	0.99965296

ZBTB12	ENSG00000204366	3.62E-05	0.99856552	9.95E-01	-0.000713582	royalblue	0.99950551
CATSPERE	ENSG00000179397	5.35E-05	0.99837824	0.994167737	-0.001078072	purple	0.99925302
SNRNP35	ENSG00000184209	0.00064436	0.995151	0.979767433	-0.001769741	purple	0.99877406
DDX18	ENSG00000088205	0.00143015	0.99403486	0.969861529	-0.001945875	purple	0.99865213
PLA2G12A	ENSG00000123739	0.00048874	0.99598492	0.982378745	-0.002094267	purple	0.99854942
SNX12	ENSG00000147164	0.00142146	0.99403486	0.969953108	-0.002147125	purple	0.99851283
HCCS	ENSG00000004961	0.0012529	0.99423295	0.971790084	-0.002210611	purple	0.99846889
S100A11	ENSG00000163191	0.00027841	0.99685673	0.986699945	-0.002900603	purple	0.99799148
ZNF48	ENSG00000180035	0.00291179	0.99186549	0.957006439	-0.003364651	purple	0.99767052
SMIM19	ENSG00000176209	0.00235233	0.99245878	0.96135322	-0.003389007	purple	0.99765368
WRNIP1	ENSG00000124535	0.00483023	0.98944013	0.944643575	-0.00403944	purple	0.99720399
UPF3A	ENSG00000169062	0.00297832	0.99185364	0.956518533	-0.00485529	midnightblue	0.99664023
VWA8	ENSG00000102763	0.00387096	0.99076511	0.95043636	-0.005208803	midnightblue	0.99639604
TGFBR2	ENSG00000163513	0.00030551	0.99670079	0.98606772	-0.005381766	midnightblue	0.99627659
NIPSNAP1	ENSG00000184117	0.00624479	0.98872623	0.937072517	-0.006796508	purple	0.9953001
TUG1	ENSG00000253352	0.0124869	0.98455129	0.911109412	-0.006800532	midnightblue	0.99529732
GMPS	ENSG00000163655	0.01107607	0.9856621	0.916261789	-0.007964746	salmon	0.99449447
STK4-AS1	ENSG00000227477	0.00544405	0.98888258	0.941237457	-0.010126232	purple	0.99300561
GPRIN3	ENSG00000185477	0.00135246	0.99408269	0.970691146	-0.010234041	midnightblue	0.9929314
SEPTIN2	ENSG00000168385	0.04360369	0.96651963	0.83475131	-0.010244819	purple	0.99292399
RAC3	ENSG00000169750	0.00366043	0.99087695	0.951801297	-0.010551458	salmon	0.99271297
SLC39A10	ENSG00000196950	0.0111872	0.9856621	0.915844303	-0.01082662	purple	0.99252365
C2	ENSG00000166278	0.00099727	0.99464019	9.75E-01	-0.011043523	royalblue	0.99237444
NETO2	ENSG00000171208	0.00095708	0.99464019	0.975343099	-0.011763841	purple	0.99187908
MMP15	ENSG00000102996	0.00183658	0.99335896	0.965848755	-0.011914882	salmon	0.99177524
RIBC1	ENSG00000158423	0.02567016	0.97413289	0.872829185	-0.014369059	salmon	0.99008956
HMGB1P5	ENSG00000132967	0.01352124	0.98430341	0.907517046	-0.015498079	midnightblue	0.98931504
CBWD2	ENSG00000136682	0.04161584	0.96722338	0.838508621	-0.016249709	purple	0.98879976

GPD1	ENSG00000167588	0.01831422	0.97984531	0.892452531	-0.016947791	purple	0.98832142
HNRNPA1L2	ENSG00000139675	0.04753625	0.96514702	0.827573139	-0.017473639	midnightblue	0.98796125
SH2B1	ENSG00000178188	0.08318005	0.95292669	0.773256221	-0.018030646	midnightblue	0.98757988
SOX12	ENSG00000177732	0.02366563	0.97545746	0.877854532	-0.018799361	salmon	0.98705381
FAM161A	ENSG00000170264	0.02184403	0.9767861	0.882613912	-0.018941876	salmon	0.98695631
NKIRAS2	ENSG00000168256	0.11256195	0.94015961	0.737508522	-0.019213084	purple	0.98677079
CENPS-CORT	ENSG00000251503	0.02799059	0.97316346	0.867257114	-0.01936842	salmon	0.98666455
EXO5	ENSG00000164002	0.01048283	0.98596532	0.918527099	-0.020000891	purple	0.9862321
MED4	ENSG00000136146	0.05686381	0.9612418	0.811705525	-0.020191559	midnightblue	0.98610176
SHLD2	ENSG00000122376	0.08779604	0.95057519	0.767227437	-0.020279461	purple	0.98604168
FITM1	ENSG00000139914	0.02225849	0.97643787	0.881513714	-0.020530484	midnightblue	0.98587013
EFCAB2	ENSG00000203666	0.02958394	0.97227205	0.863567473	-0.02095494	purple	0.98558012
TEF	ENSG00000167074	0.03570227	0.96976929	0.850274672	-0.021029529	midnightblue	0.98552917
IFT88	ENSG00000032742	0.04413569	0.96624007	0.833760985	-0.021390222	midnightblue	0.9852828
MROH2A	ENSG00000185038	0.00198131	0.99320662	9.65E-01	-0.021652235	royalblue	0.98510388
SBSPON	ENSG00000164764	0.02961072	0.97226159	0.86350635	-0.021964747	midnightblue	0.98489051
SAP18	ENSG00000150459	0.0837949	0.95257163	0.77244288	-0.02221979	midnightblue	0.98471641
TMEM14DP	ENSG00000214881	0.03316009	0.97035222	0.855642595	-0.022239362	purple	0.98470305
ABHD11	ENSG00000106077	0.04525688	0.96559053	0.831694109	-0.022978394	purple	0.98419876
TUBGCP3	ENSG00000126216	0.08429527	0.95218045	0.771783366	-0.023080929	midnightblue	0.98412882
DPY19L1	ENSG00000173852	0.03754044	0.96864941	0.846515628	-0.02371388	salmon	0.98369715
ZNF583	ENSG00000198440	0.01126698	0.9856621	0.915545903	-0.024229874	purple	0.98334538
DNLZ	ENSG00000213221	0.02292245	0.97612714	0.879772826	-0.025730997	midnightblue	0.98232274
BPHL	ENSG00000137274	0.04412203	0.96624007	0.833786339	-0.02719602	purple	0.98132572
BOLA1	ENSG00000178096	0.05453898	0.96263375	0.815523635	-0.027707656	purple	0.98097777
SLAIN1	ENSG00000139737	0.07751641	0.95406776	0.780906437	-0.028424636	midnightblue	0.98049037
FOXK2	ENSG00000141568	0.23171243	0.90631812	0.630649782	-0.028614471	purple	0.98036136
VIPAS39	ENSG00000151445	0.15327141	0.92764801	0.695739582	-0.028701098	midnightblue	0.9803025

ASB5	ENSG00000164122	0.00610213	0.98872623	9.38E-01	-0.029200053	royalblue	0.97996352
CNNM3	ENSG00000168763	0.29308433	0.89067733	0.588700511	-0.03119902	salmon	0.97860664
CDADC1	ENSG00000102543	0.14715441	0.92878011	0.701573811	-0.031254925	midnightblue	0.97856872
TTC27	ENSG00000018699	0.21823176	0.90955113	0.640770121	-0.031418753	purple	0.97845761
TIAM1	ENSG00000156299	0.00870229	0.98715889	0.925746016	-0.031819716	midnightblue	0.9781857
MIPEP	ENSG00000027001	0.13130904	0.93351673	0.717364213	-0.033108162	midnightblue	0.97731249
CMTR1	ENSG00000137200	0.26630318	0.89717552	0.60624797	-0.033225934	midnightblue	0.97723271
VAMP4	ENSG00000117533	0.24667307	0.90145442	0.619834615	-0.033972642	purple	0.97672705
OGG1	ENSG00000114026	0.12174587	0.936912	0.727422581	-0.034039419	salmon	0.97668184
CNP	ENSG00000173786	0.19016204	0.91770482	0.663132851	-0.034894122	purple	0.97610339
WBP4	ENSG00000120688	0.14749926	0.92878011	0.701241237	-0.035342312	midnightblue	0.9758002
THAP8	ENSG00000161277	0.12591032	0.93544694	0.722989698	-0.036196677	purple	0.9752225
TMEM19	ENSG00000139291	0.13849375	0.93092548	0.710077137	-0.036546832	purple	0.97498584
INTS6	ENSG00000102786	0.2654311	0.89717552	0.606837916	-0.036556374	midnightblue	0.97497939
SATB2	ENSG00000119042	0.0333296	0.97033007	8.55E-01	-0.036630477	royalblue	0.97492931
CDCA7L	ENSG00000164649	0.1221178	0.93682379	0.727023246	-0.037102745	salmon	0.97461022
PRUNE1	ENSG00000143363	0.19502058	0.91667215	0.659126572	-0.037386772	purple	0.97441836
TMEM116	ENSG00000198270	0.22382792	0.90753271	0.636523689	-0.037444333	purple	0.97437949
LNX2	ENSG00000139517	0.21758205	0.90996292	0.64126742	-0.038056753	midnightblue	0.97396595
PHLDA3	ENSG00000174307	0.03028252	0.9720608	0.861982113	-0.038283374	purple	0.97381297
MBTPS2	ENSG00000012174	0.15022236	0.92833287	0.698630592	-0.039678761	purple	0.97287155
KAT2A	ENSG00000108773	0.20471454	0.91466364	6.51E-01	-0.039951516	royalblue	0.97268764
VPS36	ENSG00000136100	0.21942416	0.90907467	0.639859788	-0.040360942	midnightblue	0.97241163
CDK8	ENSG00000132964	0.28472988	0.89268242	0.594059983	-0.041101162	midnightblue	0.97191284
LIG4	ENSG00000174405	0.17212741	0.922739	0.678559907	-0.041556991	midnightblue	0.9716058
ITPR1	ENSG00000150995	0.02489249	0.9744641	0.874754064	-0.041682359	midnightblue	0.97152137
TPTE2P1	ENSG00000253771	0.02260792	0.97635334	0.880594255	-0.042187609	midnightblue	0.9711812
DUSP23	ENSG00000158716	0.03830662	0.96829214	0.844977037	-0.043249553	purple	0.97046659

ITGA4	ENSG00000115232	0.1508663	0.92818909	0.698017232	-0.043286735	midnightblue	0.97044158
SLC44A1	ENSG00000070214	0.17045365	0.92292028	0.680039052	-0.043967757	midnightblue	0.96998359
COL4A3	ENSG00000169031	0.16247597	0.92524373	0.687208479	-0.044430214	midnightblue	0.96967271
PANK1	ENSG00000152782	0.0528424	0.96296015	0.818364458	-0.045579634	salmon	0.96890046
ARHGEF7	ENSG00000102606	0.345895	0.88221479	0.556941394	-0.045973571	midnightblue	0.96863593
PCGF6	ENSG00000156374	0.28095435	0.893628	0.596515246	-0.04698301	salmon	0.96795843
RAD51D	ENSG00000185379	0.41836324	0.86635957	0.518308456	-0.047666471	salmon	0.96749998
TDRD3	ENSG00000083544	0.28451677	0.89277047	0.594198009	-0.048674895	midnightblue	0.96682394
SRR	ENSG00000167720	0.47457304	0.85419972	0.491486791	-0.048914284	purple	0.96666353
DIS3	ENSG00000083520	0.34060368	0.88307097	0.55997181	-0.050557398	midnightblue	0.9655632
MESP2	ENSG00000188095	0.0290991	0.97256859	0.864679136	-0.050812708	purple	0.96539235
SP110	ENSG00000135899	0.18418576	0.91906226	0.668145269	-0.051495031	midnightblue	0.96493587
EFNA4	ENSG00000243364	0.23699999	0.90531556	0.626779229	-0.051706134	purple	0.96479469
MED31	ENSG00000108590	0.50549954	0.8480318	0.4777119	-0.051913182	purple	0.96465623
DCUN1D2	ENSG00000150401	0.4034251	0.86961364	0.525867584	-0.052282259	midnightblue	0.96440948
ZFYVE27	ENSG00000155256	1.16882019	0.73399541	0.280615671	-0.052585635	midnightblue	0.9642067
CTSS	ENSG00000163131	0.2871522	0.89178927	0.592495719	-0.05284945	purple	0.9640304
TMEM185B	ENSG00000226479	0.26700081	0.89717146	0.605776922	-0.053675325	salmon	0.9634787
PROSER1	ENSG00000120685	0.38762866	0.87342147	0.534077904	-0.053911564	midnightblue	0.96332094
GKAP1	ENSG00000165113	0.52396912	0.8442292	0.469784827	-0.05524283	midnightblue	0.96243244
DDX17	ENSG00000100201	0.39424086	0.87159159	0.53061326	-0.057451777	midnightblue	0.96095996
PKN2	ENSG00000065243	0.4306053	0.86341513	0.512255084	-0.057604744	midnightblue	0.96085808
LINC01237	ENSG00000233806	0.22067083	0.90907467	0.638911242	-0.05764805	midnightblue	0.96082923
CYP20A1	ENSG00000119004	0.39101238	0.87218274	0.532299817	-0.057916205	purple	0.96065066
HEIH	ENSG00000278970	0.80782251	0.79502558	0.369571614	-0.058728973	midnightblue	0.96010961
ZC3H13	ENSG00000123200	0.38208517	0.87476388	0.537014335	-0.058732572	midnightblue	0.96010722
HSPH1	ENSG00000120694	0.29965448	0.88956126	0.584554677	-0.059826881	midnightblue	0.95937924
ESD	ENSG00000139684	0.43320497	0.86312916	0.510985478	-0.060446338	midnightblue	0.95896739

PSPC1	ENSG00000121390	0.58698206	0.83273419	0.444262791	-0.061536035	midnightblue	0.95824334
SNRPGP14	ENSG00000236577	0.14518796	0.92941883	0.703478871	-0.062471647	midnightblue	0.9576221
TRIM13	ENSG00000204977	0.50306979	0.84826413	0.478770957	-0.062572765	midnightblue	0.95755498
FNDC3A	ENSG00000102531	0.36753531	0.87713724	0.544863961	-0.063297686	midnightblue	0.95707396
THAP12P3	ENSG00000227972	0.05599399	0.96188311	0.813124223	-0.06665945	midnightblue	0.95484638
CALML4	ENSG00000129007	0.11968012	0.93761779	0.729653097	-0.066797343	purple	0.95475512
EVL	ENSG00000196405	0.05302688	0.96287095	0.818053246	-0.067735301	midnightblue	0.95413459
ERVK13-1	ENSG00000260565	0.5596918	0.83771491	0.455040442	-0.067890657	midnightblue	0.95403185
C2CD2	ENSG00000157617	0.29617947	0.89026255	0.586740019	-0.068320347	midnightblue	0.95374775
PTPRJ	ENSG00000149177	0.04429809	0.96623192	0.83345993	-0.068361308	midnightblue	0.95372067
RNASEH2B-AS1	ENSG00000233672	0.23452259	0.90575173	0.628586001	-0.06860292	midnightblue	0.95356096
MICU2	ENSG00000165487	0.70453713	0.81418759	0.402011442	-0.069674796	midnightblue	0.95285276
SUGT1	ENSG00000165416	0.9384137	0.77253112	0.333559541	-0.069774675	midnightblue	0.9527868
STARD4	ENSG00000164211	0.38804976	0.8733387	0.533856036	-0.070223012	midnightblue	0.95249075
LAMP1	ENSG00000185896	0.5180975	0.84542634	0.472281603	-0.070352165	midnightblue	0.95240549
RNF6	ENSG00000127870	0.75605184	0.80422665	0.385344991	-0.070412485	midnightblue	0.95236567
CERKL	ENSG00000188452	0.35055045	0.88133539	0.554300855	-0.070499916	midnightblue	0.95230795
SIPA1L1	ENSG00000197555	0.5909742	0.83226388	0.442719563	-0.071324211	midnightblue	0.951764
RHPN1-AS1	ENSG00000254389	0.08552378	0.95153661	0.770173069	-0.072729471	purple	0.95083738
FOXRED2	ENSG00000100350	0.22562946	0.90753271	0.635170494	-0.07278906	salmon	0.95079811
RPL23AP7	ENSG00000240356	0.43081883	0.86341513	0.512150596	-0.073207673	purple	0.95052227
IRF9	ENSG00000213928	0.48995246	0.85132956	0.484555662	-0.073761021	midnightblue	0.95015776
GTF2H5	ENSG00000272047	0.53261872	0.84223515	0.466145375	-0.073850807	purple	0.95009863
C4orf47	ENSG00000205129	0.17442778	0.92225284	0.676540711	-0.074213887	purple	0.94985955
DCAF5	ENSG00000139990	1.52025076	0.68725658	0.218661971	-0.074243583	midnightblue	0.94984
PCCA	ENSG00000175198	0.66031061	0.8201941	0.417171545	-0.075961826	midnightblue	0.94870942
SP140L	ENSG00000185404	2.41384352	0.57722015	0.121446841	-0.078669817	midnightblue	0.94693033
HMGB1P6	ENSG00000259781	0.38382529	0.87428121	0.53608942	-0.079882382	midnightblue	0.94613478

ZNF286B	ENSG00000249459	0.28540344	0.89252962	0.593624157	-0.080126312	purple	0.94597482
OBI1	ENSG00000152193	0.44799326	0.86097734	0.503865663	-0.080193732	midnightblue	0.94593061
PAWR	ENSG00000177425	0.19999406	0.91610363	0.655086852	-0.080856138	midnightblue	0.9454964
SASH3	ENSG00000122122	0.16077145	0.92565974	0.688766739	-0.080872839	midnightblue	0.94548545
MIR5571	ENSG00000264824	0.0327752	0.9703608	0.856473631	-0.081369313	midnightblue	0.94516014
CARS2	ENSG00000134905	1.30650105	0.71420404	0.254049178	-0.081878543	midnightblue	0.94482658
PPIL6	ENSG00000185250	0.42517619	0.86457169	0.514924293	-0.082006329	purple	0.9447429
ZNF205	ENSG00000122386	0.06958364	0.95765281	7.92E-01	-0.082519375	royalblue	0.94440699
C17orf78	ENSG00000278505	0.31280506	0.88701018	0.576431046	-0.082617382	purple	0.94434284
ALOX5AP	ENSG00000132965	0.04997751	0.9637944	0.823272733	-0.082692313	midnightblue	0.94429379
ABHD13	ENSG00000139826	0.89563103	0.78002406	0.344808391	-0.082855639	midnightblue	0.94418689
STRBP	ENSG00000165209	1.15397818	0.73644943	0.28368415	-0.083749222	midnightblue	0.94360226
ING1	ENSG00000153487	1.4573882	0.69411233	0.228410538	-0.083773903	midnightblue	0.94358612
CDC16	ENSG00000130177	1.01862238	0.75841939	0.313756355	-0.083857408	midnightblue	0.9435315
HS2ST1	ENSG00000153936	0.55480771	0.83855065	0.457012568	-0.084209125	midnightblue	0.94330151
LACC1	ENSG00000179630	0.23519987	0.90575173	0.628090891	-0.085288631	midnightblue	0.94259594
NADK2	ENSG00000152620	1.58746609	0.67461578	0.20878457	-0.085464814	midnightblue	0.94248083
LINC01666	ENSG00000279579	0.07511883	0.95492684	7.84E-01	-0.085589195	royalblue	0.94239958
THAP2	ENSG00000173451	0.95340532	0.76960308	0.329734552	-0.085693334	purple	0.94233156
LINC00355	ENSG00000227674	0.04713007	0.9654391	8.28E-01	-0.086974296	royalblue	0.94149524
STPG1	ENSG00000001460	0.31348617	0.88678999	0.576016443	-0.087073558	midnightblue	0.94143046
XPO4	ENSG00000132953	1.12392875	0.74131573	0.290028564	-0.087563343	midnightblue	0.94111091
UBE2D3-AS1	ENSG00000246560	0.63981435	0.82464023	0.424486453	-0.088312045	purple	0.94062264
IPO5	ENSG00000065150	1.15064018	0.73682217	0.28438012	-0.088440657	midnightblue	0.94053879
NUP88	ENSG00000108559	1.99762315	0.620491	0.158706514	-0.088816081	purple	0.94029407
USP12	ENSG00000152484	0.36183938	0.87891404	0.547994945	-0.089118078	midnightblue	0.94009726
PRPF39	ENSG00000185246	1.70436303	0.65901238	0.19283799	-0.089403042	midnightblue	0.93991159
RBCK1	ENSG00000125826	1.26426255	0.71875832	0.261851858	-0.090344875	midnightblue	0.93929818

FBXL3	ENSG00000005812	0.9940502	0.76245461	0.319653825	-0.09074531	midnightblue	0.93903751
MRPS31P4	ENSG00000250299	0.64486403	0.82342308	0.422666532	-0.090962968	midnightblue	0.93889585
LRCH1	ENSG00000136141	0.58885131	0.83239159	0.443539166	-0.091624385	midnightblue	0.9384655
RNASEH2B	ENSG00000136104	0.75177056	0.80495258	0.386691872	-0.091899488	midnightblue	0.93828656
CMPK2	ENSG00000134326	0.06773894	0.95848902	0.79485763	-0.092219933	midnightblue	0.93807818
ENO2	ENSG00000111674	0.14929911	0.92850428	0.699512644	-0.09343975	midnightblue	0.93728536
CHMP4A	ENSG00000254505	1.79010266	0.64809205	0.182047811	-0.09453451	midnightblue	0.93657439
RNFT1	ENSG00000189050	1.01167981	0.75944448	0.315408012	-0.095096894	purple	0.93620937
CCNT2	ENSG00000082258	1.82888664	0.64267794	0.177399108	-0.096031568	midnightblue	0.93560303
KANK2	ENSG00000197256	0.35653256	0.88064044	0.550942411	-0.096250146	salmon	0.93546129
PIBF1	ENSG00000083535	1.09090653	0.74610765	0.297211611	-0.096925296	midnightblue	0.93502361
WNT16	ENSG00000002745	0.17265698	0.92262839	0.678093676	-0.097380363	midnightblue	0.93472873
POC1B	ENSG00000139323	1.78087594	0.6494523	0.183174501	-0.097555673	midnightblue	0.93461515
UNK	ENSG00000132478	2.58052842	0.5603054	0.109363102	-0.099518151	midnightblue	0.93334467
CLMN	ENSG00000165959	0.39739812	0.87065099	0.528973197	-0.100373385	midnightblue	0.93279154
TM9SF2	ENSG00000125304	1.25938417	0.71923256	0.262772103	-0.101092302	midnightblue	0.93232684
COQ6	ENSG00000119723	2.19709048	0.59947152	0.139446387	-0.101218803	midnightblue	0.93224509
DOCK9	ENSG00000088387	0.67589237	0.81815759	0.411735593	-0.101433018	midnightblue	0.93210668
STT3B	ENSG00000163527	1.62875216	0.66941502	0.202980604	-0.102421087	purple	0.93146852
RPS6KA5	ENSG00000100784	0.68782494	0.81625567	0.407643263	-0.102766926	midnightblue	0.93124526
INO80E	ENSG00000169592	1.60855268	0.67154417	0.205795991	-0.102812559	midnightblue	0.9312158
HMGB1	ENSG00000189403	1.54599443	0.68249486	0.214814331	-0.104467381	midnightblue	0.93014827
NUMB	ENSG00000133961	1.87050142	0.63637643	0.172563445	-0.104485529	midnightblue	0.93013657
NADK2-AS1	ENSG00000245711	0.80815389	0.79502558	0.369473601	-0.104660652	midnightblue	0.93002368
CLDN14	ENSG00000159261	0.06910926	0.95810908	0.792840121	-0.109680449	purple	0.92679332
MZT1	ENSG00000204899	1.22193824	0.72585554	0.26997197	-0.109795301	midnightblue	0.92671954
ING4	ENSG00000111653	1.4077403	0.70067735	0.236482613	-0.110136977	midnightblue	0.92650009
KDM4C	ENSG00000107077	4.13140672	0.41803006	0.043080159	-0.112614489	midnightblue	0.9249104

PCID2	ENSG00000126226	2.22460239	0.5979056	0.137003738	-0.113106097	midnightblue	0.92459528
ABCD4	ENSG00000119688	2.46931552	0.57010299	0.117268302	-0.113902139	midnightblue	0.92408525
PAXIP1	ENSG00000157212	2.24494898	0.59586416	0.135228249	-0.114008843	salmon	0.92401691
UBL3	ENSG00000122042	1.31293691	0.71250689	0.252885707	-0.114086447	midnightblue	0.92396721
PSPC1-AS2	ENSG00000226352	0.3299664	0.88411766	0.566160709	-0.114157601	midnightblue	0.92392164
GPALPP1	ENSG00000133114	1.36092058	0.70626224	0.244415022	-0.114786906	midnightblue	0.92351871
METTL3	ENSG00000165819	2.3059672	0.59109412	0.130056805	-0.115718788	midnightblue	0.92292237
ANKHD1-EIF4EBP3	ENSG00000254996	2.16720219	0.60203031	0.142155862	-0.118905328	midnightblue	0.92088613
E2F5	ENSG00000133740	2.50073963	0.56618304	0.11497227	-0.121452811	midnightblue	0.91926148
PRXL2A	ENSG00000122378	0.59538619	0.83108471	0.441023654	-0.121701538	purple	0.91910301
COMMD3	ENSG00000148444	0.86047797	0.78625307	0.354439622	-0.122333206	midnightblue	0.91870068
MTMR6	ENSG00000139505	1.65598469	0.66537787	0.199256731	-0.12375346	midnightblue	0.91779671
LUC7L3	ENSG00000108848	1.868066	0.63672355	0.172842206	-0.128144017	midnightblue	0.91500782
ARHGAP15	ENSG00000075884	0.7175125	0.81191	0.397716849	-0.129115836	midnightblue	0.91439167
CAPN12	ENSG00000182472	0.57670337	0.83437096	0.448274848	-0.129466063	midnightblue	0.91416972
FBXW7-AS1	ENSG00000270751	0.54508491	0.84025512	0.460979	-0.130825549	midnightblue	0.91330868
LINC01058	ENSG00000225039	0.36663383	0.8774353	0.545357274	-0.131074086	midnightblue	0.91315136
PLXNB1	ENSG00000164050	0.17879849	0.92084323	0.672746921	-0.131404108	midnightblue	0.91294249
ZC3H12C	ENSG00000149289	0.15915662	0.9259315	0.690251884	-0.132084443	midnightblue	0.91251208
ZNF326	ENSG00000162664	3.07949007	0.50625038	0.080428076	-0.132193845	midnightblue	0.91244288
DNAJC15	ENSG00000120675	1.23386654	0.72391191	0.267651985	-0.132656912	midnightblue	0.91215006
DHRS1	ENSG00000157379	3.64716938	0.46132474	0.05723213	-0.132939322	midnightblue	0.91197152
PKD2	ENSG00000118762	0.86296077	0.78588273	0.353747395	-0.133953905	midnightblue	0.9113304
CUL4A	ENSG00000139842	3.59438159	0.46529741	0.05905086	-0.13410854	midnightblue	0.91123272
RAB11FIP3	ENSG00000090565	3.2765802	0.49256303	0.071395225	-0.135435823	midnightblue	0.91039477
IFT80	ENSG00000068885	2.26856734	0.59374223	0.133199631	-0.136581881	midnightblue	0.90967185
HACD4	ENSG00000188921	0.59443845	0.8311627	0.441387105	-0.137579219	midnightblue	0.90904321
MRPS31P5	ENSG00000243406	1.71319462	0.6574677	0.191692626	-0.140249296	midnightblue	0.90736235

TTC14	ENSG00000163728	2.41197666	0.57734646	0.121590319	-0.140496208	midnightblue	0.90720707
ST6GALNAC4P1	ENSG00000233469	0.43991532	0.86227603	0.507733422	-0.14069541	midnightblue	0.90708182
NUDT15	ENSG00000136159	1.72768115	0.65576789	0.189831101	-0.140911251	midnightblue	0.90694612
CCDC122	ENSG00000151773	0.6386748	0.82469386	0.424898781	-0.141364217	midnightblue	0.90666141
C22orf23	ENSG00000128346	2.7463317	0.54391566	0.098647965	-0.14138474	midnightblue	0.90664851
SPRYD7	ENSG00000123178	3.1747217	0.49967339	0.075918209	-0.14215804	midnightblue	0.90616267
LARS2-AS1	ENSG00000232455	0.40917708	0.8682638	0.522933902	-0.142601743	midnightblue	0.90588402
CAVIN4	ENSG00000170681	0.32882285	0.88440122	5.67E-01	-0.146143861	royalblue	0.90366261
HIVEP2	ENSG00000010818	1.92253199	0.62812216	0.166730119	-0.146569002	midnightblue	0.90339636
ZMYM2	ENSG00000121741	3.23233744	0.49466258	0.07332303	-0.146729783	midnightblue	0.90329568
CLN5	ENSG00000102805	3.15118143	0.50093212	0.077006944	-0.148978115	midnightblue	0.90188906
STK24	ENSG00000102572	2.37807305	0.58151049	0.124229161	-0.149319018	midnightblue	0.90167597
RSRP1	ENSG00000117616	2.21270241	0.5992603	0.138054312	-0.14960886	midnightblue	0.90149484
RBM26	ENSG00000139746	3.52271978	0.47283698	0.061619416	-0.149900453	midnightblue	0.90131265
DHX58	ENSG00000108771	0.91400832	0.77773962	0.339914707	-0.150445839	midnightblue	0.90097199
DHRS12	ENSG00000102796	2.59269885	0.55831693	0.108534439	-0.150596563	midnightblue	0.90087787
FGFR1OP2	ENSG00000111790	3.61448045	0.46386857	0.058351204	-0.151675168	midnightblue	0.90020459
EDRF1-DT	ENSG00000224023	0.82709994	0.79180784	0.363929611	-0.152111404	purple	0.89993244
ZMYM5	ENSG00000132950	2.36434023	0.58367382	0.125316168	-0.152950649	midnightblue	0.89940908
CTNS	ENSG00000040531	1.93527962	0.62658835	0.165335784	-0.153858847	purple	0.89884307
SFMBT2	ENSG00000198879	2.68289573	0.5494468	0.102604649	-0.155016211	midnightblue	0.89812228
NEK3	ENSG00000136098	1.90034258	0.63163835	0.169189601	-0.156501826	midnightblue	0.89719792
ZBTB1	ENSG00000126804	5.13573948	0.34041018	0.024235729	-0.156664322	midnightblue	0.89709687
SUPT20H	ENSG00000102710	3.37697566	0.48533523	0.067220199	-0.156803443	midnightblue	0.89701036
ELF1	ENSG00000120690	2.11505212	0.60684008	0.147027219	-0.157327212	midnightblue	0.89668476
COX5BP6	ENSG00000237082	0.4013562	0.8699771	0.526929968	-0.160273221	midnightblue	0.89485559
RBM25	ENSG00000119707	3.77434359	0.45030423	0.053091552	-0.16061189	midnightblue	0.89464555
ZRANB2	ENSG00000132485	3.87621413	0.4412679	0.050005181	-0.161230245	midnightblue	0.89426217

B3GLCT	ENSG00000187676	2.36817002	0.58348419	0.125011961	-0.164881704	midnightblue	0.89200166
LIFR-AS1	ENSG00000244968	0.49612054	0.84959223	0.481821296	-0.164976025	purple	0.89194334
MYCBP2	ENSG00000005810	2.57616017	0.56045496	0.109662231	-0.165494466	midnightblue	0.89162287
TMCO4	ENSG00000162542	4.61117352	0.3785491	0.032661342	-0.165523015	midnightblue	0.89160523
MAP4K5	ENSG00000012983	7.26184556	0.21406499	0.007488558	-0.166217571	midnightblue	0.89117609
SDR39U1	ENSG00000100445	4.68131517	0.37370286	0.031376177	-0.166803392	midnightblue	0.89081429
YLPM1	ENSG00000119596	6.3570199	0.25921982	0.012272234	-0.16828287	midnightblue	0.88990123
MAML2	ENSG00000184384	0.60984749	0.82994858	0.435534491	-0.168478767	midnightblue	0.88978041
ARGLU1	ENSG00000134884	2.16427904	0.60245366	0.142424032	-0.168642669	midnightblue	0.88967933
AGR2	ENSG00000106541	0.09925479	0.94627369	7.53E-01	-0.168794487	royalblue	0.88958571
CEACAM22P	ENSG00000230666	0.23393592	0.90579625	6.29E-01	-0.169381528	royalblue	0.8892238
SUSD6	ENSG00000100647	3.01271951	0.51332859	0.083762959	-0.170932708	midnightblue	0.88826823
NAXD	ENSG00000213995	1.5774171	0.67681134	0.210226933	-0.172080489	midnightblue	0.88756182
BRCA2	ENSG00000139618	2.28236876	0.59318007	0.13203004	-0.1728881	midnightblue	0.88706511
AHSA2P	ENSG00000173209	4.09279806	0.42192319	0.044058999	-0.173222746	midnightblue	0.88685937
NDFIP2	ENSG00000102471	2.98707412	0.51686616	0.085083492	-0.173926554	midnightblue	0.88642683
ISG15	ENSG00000187608	0.44718086	0.8610394	0.504252352	-0.174385561	midnightblue	0.88614485
PHF11	ENSG00000136147	3.13803321	0.50152637	0.077622389	-0.175720987	midnightblue	0.88532497
COG6	ENSG00000133103	3.53463607	0.4709717	0.061184146	-0.176310931	midnightblue	0.88496302
PAXBP1-AS1	ENSG00000238197	6.22735961	0.26789482	0.013180966	-0.177160593	midnightblue	0.88444198
PAN3	ENSG00000152520	3.28342536	0.4919307	0.071101873	-0.17717148	midnightblue	0.8844353
CLYBL	ENSG00000125246	2.07345693	0.61167066	0.15104855	-0.177394572	midnightblue	0.88429855
LINC00598	ENSG00000215483	1.10771808	0.74479893	0.293526544	-0.181355351	midnightblue	0.88187412
TMEM167B	ENSG00000215717	6.92730117	0.22892563	0.008981183	-0.182363792	midnightblue	0.88125791
UFM1	ENSG00000120686	4.40593508	0.39508432	0.036749659	-0.182644725	midnightblue	0.88108632
PSMA3-AS1	ENSG00000257621	6.49834946	0.25213718	0.011355155	-0.183605805	midnightblue	0.88049956
TGDS	ENSG00000088451	3.19809038	0.49718768	0.074853849	-0.185077936	midnightblue	0.87960156
PDLIM1	ENSG00000107438	0.62510336	0.82649764	0.429856219	-0.18547929	midnightblue	0.87935689

LINC01562	ENSG00000203356	0.6596816	0.82036148	0.417393222	-0.186670393	midnightblue	0.87863118
SEL1L3	ENSG00000091490	1.65839529	0.66501592	0.198931001	-0.186800416	midnightblue	0.878552
PNN	ENSG00000100941	5.89244006	0.28371926	0.015865209	-0.187588188	midnightblue	0.87807241
BBIP1	ENSG00000214413	7.94328459	0.18824639	0.005186342	-0.189589631	midnightblue	0.8768551
LINC01473	ENSG00000237877	2.70676726	0.54684345	0.101095613	-0.189802759	midnightblue	0.87672558
ERICH6B	ENSG00000165837	2.41216958	0.57734646	0.121575483	-0.190919653	midnightblue	0.8760471
SSBP2	ENSG00000145687	1.06513261	0.75153583	0.302977914	-0.191041584	midnightblue	0.87597307
DNAJC3-DT	ENSG00000247400	1.56061331	0.67980952	0.212665426	-0.19199655	midnightblue	0.87539342
HMGB1P10	ENSG00000213707	1.62147542	0.67077582	0.203989542	-0.192456262	midnightblue	0.87511453
LUC7L	ENSG00000007392	5.730924	0.29471444	0.01735654	-0.193550659	midnightblue	0.87445093
RPL7P19	ENSG00000241458	0.94174785	0.77188802	0.332703748	-0.194323698	midnightblue	0.8739825
GPR180	ENSG00000152749	1.86245449	0.63763839	0.173486488	-0.194863417	midnightblue	0.8736556
PAX5	ENSG00000196092	0.18503102	0.91892849	0.667430525	-0.195347354	midnightblue	0.87336259
MBNL2	ENSG00000139793	2.74644961	0.54391566	0.098640769	-0.195536764	midnightblue	0.87324794
ZNF74	ENSG00000185252	0.93709092	0.77269499	0.333899885	-0.196309841	purple	0.87278013
COQ2	ENSG00000173085	2.5745591	0.56045496	0.109772095	-0.200549112	salmon	0.87021928
KPNA5	ENSG00000196911	5.07992611	0.34474531	0.025012569	-0.202926949	midnightblue	0.86878618
SETDB2	ENSG00000136169	3.67816653	0.45910992	0.056192125	-0.203270896	midnightblue	0.86857908
ACACB	ENSG00000076555	1.03442786	0.75563744	0.31003824	-0.203810731	midnightblue	0.86825413
GCH1	ENSG00000131979	1.59047424	0.67398764	0.208355092	-0.204589263	midnightblue	0.86778571
ENTPD4	ENSG00000197217	3.84321304	0.44416187	0.050983568	-0.204620688	midnightblue	0.86776681
LINC01268	ENSG00000227502	0.48928153	0.85139639	0.484854636	-0.204901387	midnightblue	0.86759799
KIF13B	ENSG00000197892	4.98122659	0.35127496	0.026450667	-0.205595368	midnightblue	0.86718075
KCNRG	ENSG00000198553	2.13252109	0.60503463	0.145374688	-0.205784112	midnightblue	0.8670673
RFXAP	ENSG00000133111	3.37725582	0.48533523	0.067208923	-0.206811908	midnightblue	0.86644981
PNISR	ENSG00000132424	5.45785803	0.31723455	0.020218004	-0.207972495	midnightblue	0.86575307
LINC02516	ENSG00000261083	1.20198301	0.72967913	0.273909877	-0.211004328	purple	0.8639356
LINC00559	ENSG00000261446	0.87155157	0.78387706	0.351366462	-0.211291228	midnightblue	0.86376381

PROCA1	ENSG00000167525	3.49950107	0.47365559	0.062477089	-0.213309881	midnightblue	0.86255605
KCNJ3	ENSG00000162989	0.19547686	0.91649831	0.658753412	-0.213370241	midnightblue	0.86251997
ZNF600	ENSG00000189190	1.08933063	0.74610765	0.297560092	-0.213990405	midnightblue	0.86214928
TMC3-AS1	ENSG00000259343	4.02697113	0.42896614	0.045782603	-0.214000874	midnightblue	0.86214302
N4BP2L2	ENSG00000244754	5.35217937	0.32447293	0.021453452	-0.214465689	midnightblue	0.8618653
ANKRD10	ENSG00000088448	5.3379112	0.32478778	0.021626184	-0.214965217	midnightblue	0.86156693
HMGN2P10	ENSG00000231261	0.4768524	0.85372151	0.490449135	-0.216867908	midnightblue	0.86043141
ERCC5	ENSG00000134899	6.59003344	0.2474966	0.010798351	-0.216928453	midnightblue	0.8603953
EPS8L2	ENSG00000177106	1.11678221	0.74309815	0.291564041	-0.217109947	midnightblue	0.86028707
KRR1P1	ENSG00000237672	1.90589208	0.63109301	0.168570598	-0.217367046	midnightblue	0.86013377
MANBAL	ENSG00000101363	12.0139048	0.09087936	0.000614812	-0.217525582	midnightblue	0.86003926
MYCBP2-AS2	ENSG00000229521	0.84073459	0.78949154	0.360011131	-0.219409651	midnightblue	0.85891683
NPHP3	ENSG00000113971	6.74687388	0.23969773	0.009910282	-0.220139678	midnightblue	0.85848232
DUSP6	ENSG00000139318	0.77148513	0.80192961	0.380544787	-0.220536505	midnightblue	0.85824622
BFSP2	ENSG00000170819	1.57540552	0.67746915	0.210517083	-0.220605793	midnightblue	0.858205
AKAP11	ENSG00000023516	5.28591911	0.32888969	0.022267965	-0.222905382	midnightblue	0.85683815
DOCK9-DT	ENSG00000260992	1.48680495	0.69102543	0.22378492	-0.223751443	midnightblue	0.85633581
DLGAP4-AS1	ENSG00000232907	4.39847117	0.39564711	0.036908126	-0.224462903	midnightblue	0.85591361
UBAC2-AS1	ENSG00000228889	2.14993161	0.60401254	0.143748589	-0.225465482	midnightblue	0.85531902
NAA16	ENSG00000172766	5.34534565	0.32477012	0.021536001	-0.227237849	midnightblue	0.85426889
SERPINF2	ENSG00000167711	0.96170377	0.76808299	0.327642427	-0.227428707	purple	0.85415589
SP140	ENSG00000079263	4.74084435	0.3702335	0.030327107	-0.22770784	midnightblue	0.85399064
GAS6-AS1	ENSG00000233695	1.10289871	0.74520832	0.294576906	-0.228141201	midnightblue	0.85373415
PTPRK	ENSG00000152894	0.9302649	0.77388676	0.335663592	-0.228248682	midnightblue	0.85367055
ANKRD10-IT1	ENSG00000229152	3.3473119	0.48569623	0.068425747	-0.230905356	midnightblue	0.85209999
CASTOR1	ENSG00000239282	3.52171913	0.47283698	0.061656117	-0.232679788	midnightblue	0.8510526
LINC00571	ENSG00000223685	1.97446529	0.62250607	0.161132779	-0.233676941	midnightblue	0.85046458
BMI1	ENSG00000168283	4.79126912	0.3650362	0.029467266	-0.23416049	midnightblue	0.85017958

TTC28	ENSG00000100154	1.17730088	0.73282985	0.278881236	-0.236176948	midnightblue	0.84899211
SEC62	ENSG00000008952	8.01164664	0.18653743	0.004999687	-0.236582132	midnightblue	0.8487537
RBFOX2	ENSG00000100320	5.97003353	0.27860233	0.015196656	-0.24090439	midnightblue	0.84621468
SRSF5	ENSG00000100650	6.9195075	0.22892563	0.009019399	-0.243555856	midnightblue	0.84466088
MTCO3P44	ENSG00000248654	1.18143682	0.73219083	0.278040294	-0.243568385	midnightblue	0.84465355
MPHOSPH8	ENSG00000196199	5.92123478	0.28131258	0.015613621	-0.24501035	midnightblue	0.84380974
LINC01602	ENSG00000205293	0.32229185	0.88480943	5.71E-01	-0.245918477	royalblue	0.84327876
N4BP2L1	ENSG00000139597	3.75205974	0.45287731	0.053793343	-0.246111133	midnightblue	0.84316616
HNRNPA1P64	ENSG00000213559	0.9078235	0.7782466	0.341551108	-0.246948022	midnightblue	0.84267719
NHLRC3	ENSG00000188811	5.90754301	0.28283788	0.01573273	-0.247604216	midnightblue	0.84229399
KCNK10	ENSG00000100433	0.63059962	0.82570881	0.427838036	-0.248967688	midnightblue	0.84149833
COG3	ENSG00000136152	6.68310729	0.24209188	0.010261885	-0.249864362	midnightblue	0.84097548
ANKRD13A	ENSG00000076513	5.30124495	0.32775562	0.022076748	-0.250073731	midnightblue	0.84085344
IL17D	ENSG00000172458	2.19934167	0.59947152	0.139244682	-0.254854057	midnightblue	0.83807191
MRPS31	ENSG00000102738	5.37520392	0.32312977	0.02117774	-0.256258644	midnightblue	0.83725637
PCMTD1	ENSG00000168300	6.91937135	0.22892563	0.009020068	-0.259799949	midnightblue	0.83520373
FAM49A	ENSG00000197872	1.14138846	0.73799694	0.286320491	-0.259894581	midnightblue	0.83514894
PIGH	ENSG00000100564	8.37886791	0.17564517	0.004108528	-0.260251313	midnightblue	0.83494246
RSAD2	ENSG00000134321	0.48654884	0.85183547	0.486075522	-0.264832225	midnightblue	0.83229552
NEK8	ENSG00000160602	6.23105201	0.26761344	0.013154148	-0.264955979	midnightblue	0.83222413
RBM26-AS1	ENSG00000227354	7.33055113	0.21247929	0.007215046	-0.265061011	midnightblue	0.83216354
NHLRC4	ENSG00000257108	6.97007268	0.22751411	0.008774406	-0.266039489	midnightblue	0.83159934
ZAR1L	ENSG00000189167	2.34014369	0.5870864	0.127257374	-0.266087563	midnightblue	0.83157163
IFITM1	ENSG00000185885	1.25307259	0.72006009	0.263968691	-0.267000445	midnightblue	0.83104561
PLCG2	ENSG00000197943	6.12367556	0.27271702	0.013957607	-0.267567315	midnightblue	0.83071913
MIR186	ENSG00000207721	4.00504783	0.43035966	0.046372385	-0.271056716	midnightblue	0.82871233
LINC02728	ENSG00000251323	3.68630618	0.45810933	0.055922383	-0.272113118	midnightblue	0.82810573
CYCSP34	ENSG00000176268	1.54915484	0.6817679	0.214347578	-0.278471445	midnightblue	0.82446409

CIPC	ENSG00000198894	10.2281091	0.12940243	0.001549243	-0.2818146	midnightblue	0.82255577
BCL2L2	ENSG00000129473	11.5840795	0.09777496	0.000766913	-0.283911537	midnightblue	0.82136106
ITM2B	ENSG00000136156	5.10830346	0.34205549	0.024614399	-0.283987395	midnightblue	0.82131788
ACVR2A	ENSG00000121989	7.62696091	0.19926919	0.006147791	-0.287877559	midnightblue	0.81910621
UBAC2	ENSG00000134882	4.94077145	0.35359973	0.027064859	-0.289791197	midnightblue	0.81802044
NCOA7	ENSG00000111912	6.13764751	0.2720089	0.013850247	-0.291970927	midnightblue	0.81678545
FLT1	ENSG00000102755	0.88921261	0.78088863	0.346540049	-0.292392719	midnightblue	0.81654669
LMO7-AS1	ENSG00000261105	1.38070238	0.70306557	0.241024457	-0.2930633	midnightblue	0.81616724
LINC00562	ENSG00000260388	3.19410209	0.49737893	0.075034354	-0.294025717	midnightblue	0.81562295
LINC02576	ENSG00000232613	2.18789087	0.59947152	0.140274102	-0.297260729	midnightblue	0.8137961
BTN1A1	ENSG00000124557	0.92326956	0.7754465	0.337484058	-0.298144307	midnightblue	0.81329784
USPL1	ENSG00000132952	7.69005471	0.19635531	0.005942382	-0.298447677	midnightblue	0.81312684
C10orf143	ENSG00000237489	8.61109537	0.16403257	0.003630635	-0.299006175	midnightblue	0.81281212
PKN3	ENSG00000160447	2.60074761	0.55683938	0.107990223	-0.299805684	midnightblue	0.81236181
MIR8071-1	ENSG00000274172	0.24199684	0.90325978	0.623170291	-0.300701926	midnightblue	0.8118573
MTIF3	ENSG00000122033	10.70501	0.11799054	0.001208544	-0.301074376	midnightblue	0.81164774
CCDC18-AS1	ENSG00000223745	6.9666132	0.22751411	0.008790947	-0.301096467	midnightblue	0.81163531
USP18	ENSG00000184979	1.16013	0.73528713	0.282407162	-0.304910723	midnightblue	0.80949231
PDCD4-AS1	ENSG00000203497	5.71326678	0.29565993	0.017528175	-0.305829883	midnightblue	0.80897673
SCARNA9	ENSG00000254911	1.8635882	0.63744969	0.173356099	-0.306827886	midnightblue	0.80841731
PPM1K	ENSG00000163644	10.3351308	0.12808308	0.00146511	-0.310279001	midnightblue	0.80648578
ADGRL2	ENSG00000117114	0.3567163	0.88064044	0.550839865	-0.31463771	midnightblue	0.80405288
IGLV1-62	ENSG00000253823	0.61273879	0.82899614	0.434449592	-0.315581204	midnightblue	0.80352722
IL26	ENSG00000111536	2.26764618	0.59374223	0.133278107	-0.315635213	midnightblue	0.80349714
N4BP2L2-IT2	ENSG00000281026	8.65304842	0.16386161	0.003550567	-0.317101246	midnightblue	0.80268106
DUSP13	ENSG00000079393	0.59021867	0.83226388	4.43E-01	-0.318469469	royalblue	0.80192017
SLC15A2	ENSG00000163406	5.22608011	0.33294913	0.023031255	-0.320270686	midnightblue	0.80091959
SLC25A30	ENSG00000174032	9.09788221	0.15297416	0.002804881	-0.321405619	midnightblue	0.80028978

ZP1	ENSG00000149506	0.9759259	0.76615362	0.324097761	-0.32219539	purple	0.79985179
SPART	ENSG00000133104	2.12111772	0.60666155	0.146451025	-0.331428497	midnightblue	0.79474917
PRICKLE1	ENSG00000139174	5.97357719	0.27822222	0.01516683	-0.332588113	midnightblue	0.79411062
GSAP	ENSG00000186088	11.7553776	0.09517333	0.00070217	-0.332825649	midnightblue	0.79397988
TMEFF1	ENSG00000241697	0.77863504	0.80026604	3.78E-01	-0.333834974	royalblue	0.7934246
SEPTIN9	ENSG00000184640	1.94396248	0.62561419	0.164393726	-0.334585155	midnightblue	0.79301213
RB1	ENSG00000139687	3.90298154	0.43889084	0.049226265	-0.336353002	midnightblue	0.79204099
ZDHHC4P1	ENSG00000238025	2.29400066	0.59166578	0.13105325	-0.338842192	midnightblue	0.7906756
LINC01359	ENSG00000226891	3.33949037	0.48614174	0.068747477	-0.339342997	midnightblue	0.79040118
SNORA31	ENSG00000199477	5.12571278	0.3410048	0.024373407	-0.353703565	midnightblue	0.78257256
AGRN	ENSG00000188157	2.68033446	0.54973707	0.102768026	-0.355158755	midnightblue	0.78178361
TPT1-AS1	ENSG00000170919	9.44488123	0.14573404	0.002335731	-0.357370743	midnightblue	0.78058587
UBE2L5	ENSG00000236444	2.46858052	0.57017745	0.117322611	-0.357800947	midnightblue	0.78035314
TPTE2P5	ENSG00000168852	3.21404467	0.49604711	0.074136454	-0.358962493	midnightblue	0.77972511
STOX1	ENSG00000165730	1.91349594	0.63009219	0.167726677	-0.359518458	midnightblue	0.77942469
RPS16P5	ENSG00000217527	2.88674438	0.52618364	0.090471669	-0.361472087	midnightblue	0.77836995
PRELID3BP4	ENSG00000251163	2.79209433	0.53916876	0.09589764	-0.361988375	midnightblue	0.77809145
MIR3671	ENSG00000265996	1.44187006	0.69630917	0.230897185	-0.366634053	midnightblue	0.77558991
GPR62	ENSG00000180929	3.51800809	0.47294603	0.061792435	-0.369756944	midnightblue	0.77391287
LINC02102	ENSG00000248677	3.67059959	0.46019343	0.056444133	-0.371115815	midnightblue	0.77318427
SUGT1P3	ENSG00000239827	4.16441475	0.41537961	0.042261554	-0.375043223	midnightblue	0.77108231
NLGN4X	ENSG00000146938	0.50002863	0.84894587	0.480101944	-0.376957371	midnightblue	0.77005993
PPM1K-DT	ENSG00000246375	3.98604244	0.43183058	0.046890189	-0.382060869	midnightblue	0.76734067
ORAOV1P1	ENSG00000251008	3.28389409	0.4919307	0.071081832	-0.382759156	midnightblue	0.76696936
HERC5	ENSG00000138646	6.53731569	0.25007055	0.011114973	-0.384813223	midnightblue	0.76587815
ANKRD36BP2	ENSG00000230006	4.90409495	0.35587668	0.027634584	-0.386108252	midnightblue	0.76519097
PAN3-AS1	ENSG00000261485	4.51488394	0.38643674	0.034516361	-0.386494407	midnightblue	0.76498618
PIGL	ENSG00000108474	19.3749567	0.03447327	1.55E-05	-0.386642874	midnightblue	0.76490746

IL22	ENSG00000127318	3.37222594	0.48533523	0.067411684	-0.387268129	midnightblue	0.76457603
XAF1	ENSG00000132530	2.18497437	0.59964123	0.140537665	-0.391598805	midnightblue	0.76228437
IFI44	ENSG00000137965	1.3145009	0.71232465	0.252603964	-0.39300165	midnightblue	0.7615435
STARD13	ENSG00000133121	3.47766233	0.47659107	0.06329547	-0.395091276	midnightblue	0.76044126
ZDHHC14	ENSG00000175048	4.96853375	0.35197985	0.026641785	-0.4022673	midnightblue	0.75666819
MIR548AR	ENSG00000264539	1.75132063	0.65406274	0.186838655	-0.404112377	midnightblue	0.7557011
MTND4P9	ENSG00000250050	5.27477071	0.32921541	0.022408145	-0.405184171	midnightblue	0.75513989
ITGA6-AS1	ENSG00000232788	9.48494353	0.14527608	0.002286997	-0.410631911	midnightblue	0.75229379
IFIT1P1	ENSG00000215515	6.21481683	0.26841483	0.01327249	-0.410687532	midnightblue	0.75226479
SCD5	ENSG00000145284	1.36986135	0.70500225	0.24287541	-0.416649817	midnightblue	0.74916229
ECHDC3	ENSG00000134463	1.77673739	0.65019867	0.183682504	-0.416844404	midnightblue	0.74906125
DDX60L	ENSG00000181381	2.19388108	0.59947152	0.139734519	-0.417675179	midnightblue	0.74863003
DDX60	ENSG00000137628	1.93440656	0.62658835	0.165430851	-0.420714474	midnightblue	0.74705457
GPCPD1	ENSG00000125772	16.9964942	0.04498225	5.00E-05	-0.423968649	midnightblue	0.74537139
PALD1	ENSG00000107719	1.08034897	0.74780323	0.299556317	-0.426173491	midnightblue	0.74423313
IFIT1	ENSG00000185745	2.00723172	0.61978872	0.157712105	-0.427847071	midnightblue	0.74337029
L3HYPDH	ENSG00000126790	9.56872801	0.14338504	0.002188405	-0.4285375	midnightblue	0.74301462
MOXD1	ENSG00000079931	1.92884784	0.6272121	0.166037609	-0.436897499	midnightblue	0.73872152
RGS13	ENSG00000127074	0.64073221	0.82443704	0.424154779	-0.43897285	midnightblue	0.73765961
FMNL3	ENSG00000161791	7.58360898	0.20205415	0.006293143	-0.44246197	midnightblue	0.73587776
KLHDC8A	ENSG00000162873	1.1004925	0.74557421	0.295103138	-0.443857202	midnightblue	0.73516643
CIR1P2	ENSG00000250215	2.66465407	0.55116873	0.103774496	-0.447811169	midnightblue	0.73315433
PWAR1	ENSG00000279050	5.83997136	0.28804896	0.016334511	-0.448735418	midnightblue	0.7326848
EPSTI1	ENSG00000133106	5.11594741	0.34205549	0.024508281	-0.450382368	midnightblue	0.73184886
C16orf74	ENSG00000154102	1.80321882	0.64657626	0.180460024	-0.460869076	midnightblue	0.72654846
LINC01781	ENSG00000234184	1.10091667	0.74557421	0.295010284	-0.466764581	midnightblue	0.72358551
OR2A42	ENSG00000212807	4.77580482	0.36634065	0.029728212	-0.469519503	midnightblue	0.72220509
PIK3AP1	ENSG00000155629	5.61750524	0.30401069	0.018490224	-0.472774424	midnightblue	0.72057753

LINC02541	ENSG00000230943	2.04531311	0.61398122	0.153840362	-0.47557969	midnightblue	0.71917776
MTND3P5	ENSG00000251353	3.49644603	0.4742833	0.062590888	-0.47666474	midnightblue	0.71863707
PRAL	ENSG00000279296	3.86876865	0.44197273	0.05022416	-0.481544201	midnightblue	0.71621061
LINC01888	ENSG00000237576	5.59995303	0.30536556	0.018672429	-0.500089943	midnightblue	0.7070627
ZNF804A	ENSG00000170396	3.23028877	0.49466258	0.073413648	-0.506204984	midnightblue	0.70407207
CCDC146	ENSG00000135205	16.1442208	0.0530671	7.63E-05	-0.514538546	midnightblue	0.7000168
ANO9	ENSG00000185101	2.5705879	0.56100322	0.110045122	-0.518033358	midnightblue	0.69832312
RCBTB2	ENSG00000136161	7.6374252	0.19835893	0.006113226	-0.519802876	midnightblue	0.69746713
NPM1P25	ENSG00000229417	13.1923659	0.07513653	0.000336752	-0.523672654	midnightblue	0.6955988
LINC01310	ENSG00000205632	3.62818213	0.4624797	0.05787932	-0.537213657	midnightblue	0.68910052
CDH23	ENSG00000107736	6.40768142	0.25663182	0.011934998	-0.563188979	midnightblue	0.67680448
LINC00621	ENSG00000262619	3.21534383	0.49604711	0.074078364	-0.573711297	midnightblue	0.67188615
EID3	ENSG00000255150	4.37194546	0.39703159	0.037477147	-0.580781058	midnightblue	0.66860171
ABI3BP	ENSG00000154175	3.25096888	0.49315782	0.072504438	-0.594862627	midnightblue	0.6621075
SLC46A3	ENSG00000139508	6.86134362	0.23167073	0.009309946	-0.600218756	midnightblue	0.65965393
MYO5C	ENSG00000128833	7.94478644	0.18824639	0.005182166	-0.608202925	midnightblue	0.65601335
NCOA4P2	ENSG00000249934	7.85413593	0.19043982	0.005440548	-0.608767507	midnightblue	0.65575668
SAMD7	ENSG00000187033	4.68033778	0.37370286	0.031393716	-0.649198616	midnightblue	0.63763441
CCND1	ENSG00000110092	1.42928264	0.69840055	0.23293832	-0.660720406	midnightblue	0.63256235
IFIT3	ENSG00000119917	4.32194947	0.40133146	0.038574934	-0.692581677	midnightblue	0.61874562
IFI44L	ENSG00000137959	2.81226706	0.5362034	0.094712035	-0.696776693	midnightblue	0.61694907
SYCP2L	ENSG00000153157	10.0579424	0.1322138	0.001693291	-0.74005916	midnightblue	0.5987148
STAP1	ENSG00000035720	11.3193537	0.10132658	0.000879138	-0.758263654	midnightblue	0.59120745
MIR339	ENSG00000199023	7.07218782	0.22336871	0.008300331	-0.768425649	midnightblue	0.58705776
NUS1P2	ENSG00000234685	3.59039079	0.46552827	0.059190847	-0.777024355	midnightblue	0.5835692
TRIM22	ENSG00000132274	8.11376571	0.18450799	0.004733603	-0.783454737	midnightblue	0.5809739
MIR4420	ENSG00000264773	1.98523144	0.62135424	0.15999956	-0.80193743	midnightblue	0.57357839
ZC3H12D	ENSG00000178199	6.86092044	0.23167073	0.009312095	-0.81186318	midnightblue	0.56964571

MTMR9LP	ENSG00000220785	8.73414147	0.16304475	0.003400883	-0.839175268	midnightblue	0.55896302
FLT3	ENSG00000122025	5.14148566	0.33995758	0.024157194	-0.841820533	midnightblue	0.55793906
CHST3	ENSG00000122863	4.05967032	0.4259788	0.044917654	-0.865372819	midnightblue	0.54890454
MIR320D1	ENSG00000211491	4.53176288	0.38510388	0.03418335	-0.93799115	midnightblue	0.52195917
KCNMB2	ENSG00000197584	5.61303532	0.30428019	0.018536449	-0.955111483	midnightblue	0.51580173
LINC00996	ENSG00000242258	14.9556764	0.06518483	0.000138218	-1.038713665	midnightblue	0.48676129
XXYLT1-AS2	ENSG00000230266	7.54935941	0.20368389	0.006410471	-1.141307862	midnightblue	0.45334841
