# Supplementary material for: Multivariant Transcriptome Analysis Identifies Modules and Hub Genes Associated with Poor Outcomes in Newly Diagnosed Multiple Myeloma Patients
Source: Cancers (Basel). 2022 Apr 29;14(9):2228. doi: 10.3390/cancers14092228 (PMC9104534; doi:10.3390/cancers14092228)
Supplement: Supplementary file 1 [file cancers-14-02228-s001.zip › Table S3.pdf]

Table S3. Epigenomics Roadmap HM ChIP-seq table depicts number of genes from royalblue that overlap with genes reference in collection of human epigenomes for primary cells and tissues. The reference database was filtered by the fisher exact p-value.

Term	Overlap	*p*-value	Odds Ratio	Combined Score	Genes
H3K9me3 Adipose Derived Mesenchymal Stem Cell Cultured Cells	3/247	6.12×10^{-04}	22.0663189	163.247554	HTR2C; MAGEC2; GABRG2
H3K9me3 Neurosphere Cultured Cells Cortex Derived	2/340	2.29×10^{-2}	9.68836292	36.583811	HTR2C; SSX1
H3K9me3 H9	2/344	2.34×10^{-2}	9.57309942	35.9395531	GABRA3; SSX1
H3K27me3 Adipose Nuclei	4/1174	7.36×10^{-3}	6.43282051	31.5991908	GABRA3; MAGEC2; GABRG2; GABRG1
H3K27me3 Breast Myoepithelial Cells	4/1292	1.03×10^{-2}	5.8068323	26.5791401	GABRA3; HTR2C; MAGEC2; GABRG2
H3K9me3 IMR90	3/814	1.75×10^{-2}	6.44826813	26.097062	GABRB2; HTR2C; MAGEC2
H3K27me3 Mobilized CD34 Primary Cells	7/53	2.08×10^{-2}	3.49527665	13.5339638	GABRB2; SOHLH1; GABRA3; MAGEA1; HTR2C; GABRG2; GABRG1
