# Supplementary material for: Multivariant Transcriptome Analysis Identifies Modules and Hub Genes Associated with Poor Outcomes in Newly Diagnosed Multiple Myeloma Patients
Source: Cancers (Basel). 2022 Apr 29;14(9):2228. doi: 10.3390/cancers14092228 (PMC9104534; doi:10.3390/cancers14092228)
Supplement: Supplementary file 1 [file cancers-14-02228-s001.zip › Table S4.pdf]

Table S4. The table is a subset of genes with p-value less than 0.05 and kME greater than 0.7. The first column to the left depicts all the genes that are differentially significant (p<0.05) with the modules' colors as background. The last column (kME) with background rows depicts genes that satisfy both cutoff points. Nine genes from the three modules meet the cutoff points. Five genes (GABRA3, MAGEA6, MAGEA1, HTR2C and CTAG2) from royalblue; four genes (NEK2, KIF14, CENPF and RRM2) from salmon module, and zero gene (0) from purple module meet both cutoff points.

**UniqueID**	**F-Value**	**FDR (BH)**	**P-value**	**NETcolors**	**Fold Change**	**kME**
NEK2	14.8821512	0.06518483	1.4×10^{-4}	salmon	1.96988507	0.88964613
KIF14	17.1945878	0.04201811	4.53×10^{-5}	salmon	1.87196404	0.88515559
CENPF	15.7973229	0.05782571	9.07×10^{-5}	salmon	1.87982766	0.87578245
GABRA3	13.4449923	0.07513653	2.96×10^{-4}	royalblue	2.68333895	0.80449542
RRM2	15.3072578	0.06332009	1.16×10^{-4}	salmon	1.88772793	0.79906078
MAGEA6	13.5133196	0.07513653	2.86×10^{-4}	royalblue	3.68868921	0.77976914
MAGEA1	9.03799594	0.15379295	2.90×10^{-3}	royalblue	2.93020327	0.7200529
HTR2C	11.8591168	0.09386565	6.66×10^{-4}	royalblue	2.7365129	0.71580957
CTAG2	14.6939923	0.06518483	1.58×10^{-4}	royalblue	3.91527088	0.70009416
SOHLH1	15.3802021	0.06331129	1.12×10^{-4}	royalblue	3.34303231	0.6762739
FAM72C	13.782331	0.07410552	2.50×10^{-4}	salmon	1.85106907	0.65980026
CASC9	10.0888844	0.13216723	1.67×10^{-3}	royalblue	2.76883863	0.64651398
NES	4.91043512	0.35533606	2.75×10^{-2}	purple	2.60953545	0.61447718
AFAP1-AS1	8.61147695	0.16403257	3.63×10^{-3}	royalblue	3.06153157	0.60704947
CCND2	5.37464945	0.32312977	2.12×10^{-2}	purple	2.91696707	0.58697277
CD109	7.5785211	0.20228779	6.31×10^{-3}	purple	2.06580113	0.49279116
RNU6-583P	5.8954236	0.28371926	1.58×10^{-2}	salmon	1.87731759	0.48424885
KIF7	17.7334367	0.0392808	3.47×10^{-5}	salmon	2.16963657	0.46775778

NTRK1	29.2401024	0.00433237	$1.42x10^{-7}$	purple	3.10571181	0.46018116
PKP2	5.82230782	0.28915181	$1.65x10^{-2}$	purple	2.46721331	0.44459126
TMSB15A	7.086524	0.22320869	$8.24x10^{-3}$	salmon	2.0862709	0.3793585
CBX2	21.7114638	0.01529222	$5.00x10^{-6}$	salmon	2.52631878	0.37773077
LINC00484	16.2865821	0.05058776	$7.11x10^{-5}$	salmon	2.18544446	0.35595025
CRISPLD1	15.5875782	0.0616416	$1.01x10^{-4}$	purple	2.15163168	0.32354243
C1orf226	10.3334308	0.12808308	$1.47x10^{-3}$	purple	2.32446278	0.31465535
GABRB2	18.0265463	0.03832796	$3.01x10^{-5}$	royalblue	3.34865045	0.30714786
NCALD	8.87001082	0.1596802	$3.16x10^{-3}$	purple	1.99659787	0.30456606
DCDC1	18.9452947	0.03447327	$1.92x10^{-5}$	purple	2.26879342	0.30426801
GLDC	11.1151975	0.10359968	$9.77x10^{-4}$	royalblue	2.69406938	0.30404348
TGFB2	7.51592064	0.205261	$6.53x10^{-3}$	purple	2.20441988	0.29801443
