# Supplementary material for: Multivariant Transcriptome Analysis Identifies Modules and Hub Genes Associated with Poor Outcomes in Newly Diagnosed Multiple Myeloma Patients
Source: Cancers (Basel). 2022 Apr 29;14(9):2228. doi: 10.3390/cancers14092228 (PMC9104534; doi:10.3390/cancers14092228)
Supplement: Supplementary file 1 [file cancers-14-02228-s001.zip › Table S5.pdf]

Table S5A. Pearson correlation for hub and differentially significant genes in royalblue module (M20). The table depicts the liner correlation between the hub genes in the M20.

Affymetrix ID	CTAG2	GABRA3	MAGEA1	MAGEA6	HTR2C
**CTAG2**	1 ($p<1x10^{-04}$)				
**GABRA3**	0.6552 ($p<1x10^{-04}$)	1 ($p<1x10^{-04}$)			
**MAGEA1**	0.6456 ($p<1x10^{-04}$)	0.6289 ($p<1x10^{-04}$)	1 ($p<1x10^{-04}$)		
**MAGEA6**	0.6363 ($p<1x10^{-04}$)	0.9012 ($p<1x10^{-04}$)	0.5964 ($p<1x10^{-04}$)	1 ($p<1x10^{-04}$)	
**HTR2C**	0.5693 ($p<1x10^{-04}$)	0.5967 ($p<1x10^{-04}$)	0.5939 ($p<1x10^{-04}$)	0.5106 ($p<1x10^{-04}$)	1 ($p<1x10^{-04}$)

Table S5B. Pearson correlation for hub and differentially significant genes in salmon module (M13). The table depicts the liner correlation between the hub genes in the M13.

Affymetrix ID	NEK2	CENPF	KIF14	RRM2
**NEK2**	1 ($p<1x10^{-04}$)			
**CENPF**	0.9176 ($p<1x10^{-04}$)	1 ($p<1x10^{-04}$)		
**KIF14**	0.9145 ($p<1x10^{-04}$)	0.9352 ($p<1x10^{-04}$)	1 ($p<1x10^{-04}$)	
**RRM2**	0.7796 ($p<1x10^{-04}$)	0.8067 ($p<1x10^{-04}$)	0.8115 ($p<1x10^{-04}$)	1 ($p<1x10^{-04}$)
