# Supplementary material for: Multivariant Transcriptome Analysis Identifies Modules and Hub Genes Associated with Poor Outcomes in Newly Diagnosed Multiple Myeloma Patients
Source: Cancers (Basel). 2022 Apr 29;14(9):2228. doi: 10.3390/cancers14092228 (PMC9104534; doi:10.3390/cancers14092228)
Supplement: Supplementary file 1 [file cancers-14-02228-s001.zip › Table S6.pdf]

Tables 6a and 6b depict the AUC of the genes from the royalblue module. The AUC of table 6b GEO dataset validates the AUC values of table 6a MMRF dataset. The genes are not a promising Biomarker even though they are hub genes.

Table S6A. Receiver Operative Curve MMRF dataset

**MMRF Compass Dataset**						
**Marker**	**AUC**	**SE.AUC**	**Lower Limit**	**Upper Limit**	**z**	**p-value**
GABRA3	0.63484	0.04271	0.55112	0.71856	3.15689	1.60×10^{-3}
CTAG2	0.64854	0.04181	0.5666	0.73049	3.5529	3.8×10^{-4}
MAGEA1	0.62911	0.0411	0.54855	0.70966	3.14134	1.68×10^{-3}
GABRB2	0.66859	0.04022	0.58976	0.74742	4.19179	3.0×10^{-5}
GLDC	0.63476	0.04066	0.55507	0.71445	3.31424	9.2×10^{-4}

Table S6B. Receiver Operative Curve GSE83503 dataset

**GSE83503 Micro-Array Dataset**						
**Marker**	**AUC**	**SE.AUC**	**Lower Limit**	**Upper Limit**	**z**	**p-value**
GABRA3	0.61784	0.02629	0.56631	0.66937	4.48192	1.0×10^{-5}
CTAG2	0.52357	0.02805	0.4686	0.57854	0.84033	4.0×10^{-1}
MAGEA1	0.5294	0.02717	0.47614	0.58267	1.08208	2.79×10^{-1}
GABRB2	0.59936	0.02688	0.54668	0.65205	3.69628	2.2×10^{-4}
GLDC	0.5228	0.02749	0.46893	0.57667	0.82943	4.1×10^{-1}
